# Synergistic Interplay of Acceptor and Isovalent Co‐Doping on BaZrO_3_‐Based Proton Conducting Oxides: A First‐Principles Study

**DOI:** 10.1002/advs.202524188

**Published:** 2026-04-03

**Authors:** Yonghun Shin, Kyung‐Yeon Doh, June Ho Lee, Shin Hyun Kim, Donghwa Lee

**Affiliations:** ^1^ Department of Materials Science and Engineering Pohang University of Science and Technology Pohang South Korea; ^2^ Division of Advanced Materials Science Pohang University of Science and Technology Pohang South Korea

**Keywords:** dehydration at elevated temperatures, first‐principles density functional theory(DFT) calculations, perovskite oxide BaZrO_3_, proton‐conducting oxide(PCO), synergistic interaction of acceptor and isovalent Co‐doping

## Abstract

Acceptor‐doped BaZrO_3_ (BZO) with an ABO3‐type structure is a promising proton‐conducting oxide (PCO) for fuel cells and electrolyzers. However, dehydration at elevated temperatures significantly reduces proton concentration, hindering commercialization. While recent studies have shown that isovalent co‐doping in acceptor‐doped systems improves hydration performance, the dehydration remains unresolved due to a lack of fundamental understanding of these co‐doping effects. In this study, first‐principles density functional theory calculations were employed to systematically investigate how acceptor and isovalent co‐doping affects the hydration performance of BZO at the atomic scale. Analysis of trivalent acceptors at the **B‐**site ($M_{Zr}^{\prime }$) shows that acceptors suppress dehydration by inhibiting oxygen vacancy formation through reduced M─O bonding lengths, with $M_{Zr}^{\prime }$= Yb and Tm showing the most favorable hydration energy. Isovalent co‐dopings at either the **B‐**site ($N_{Zr}^{\times }$) or **A‐**site ($N_{Ba}^{\times }$) further enhance hydration by stabilizing protons via increased distance between A‐site cations and protons, with $N_{Zr}^{X}$= Th and $N_{Ba}^{X}$= Ca showing the most effective proton stabilization. The synergistic interaction of acceptors inhibiting oxygen vacancy formation and isovalent dopants stabilizing protons enables precise tailoring of hydration energy. Based on these complementary effects, we suggest four promising co‐doping combinations (Yb─Ca, Yb─Th, Tm─Ca, and Tm─Th) as superior alternatives to conventional Y─Ce pairs. Our findings provide co‐doping design guidelines for developing high‐performance PCOs.

## Graphical abstract

Acceptor and isovalent co‐doping effects on hydration in BaZrO_3_ proton conductors are systematically investigated using first‐principles calculations. Acceptors enhance hydration by modulating oxygen vacancy formation, whereas isovalent dopants enhance hydration by stabilizing protons via increased A‐site cation–proton distances. Their synergistic interaction enables tailoring hydration behavior, identifying Yb─Ca, Yb─Th, Tm─Ca, and Tm─Th as promising co‐doping pairs.

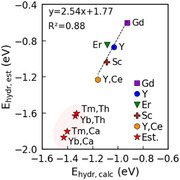

## 1 Introduction

Perovskite proton‐conducting oxides (PCOs) that exhibit proton conductivity under hydrogen‐containing atmosphere have received considerable attention for their potential applications, including an electrolyte in protonic ceramic fuel cell and electrolysis cell, and a membrane for gas separation [1, 2, 3, 4, 5, 6, 7, 8, 9, 10, 11, 12]. For these applications, BaCeO_3_‐based PCOs have been studied extensively. However, chemical instability originated from the high reactivity of cerium (Ce) with atmospheric H_2_O, and CO_2_ has limited their practical application [13, 14, 15, 16]. Instead, BaZrO_3_(BZO)‐based electrolytes, where Ce is switched by zirconium (Zr), have been reported to show superior chemical and environmental stability, so it receives attention as alternatives to the BaCeO_3_‐based PCO [17, 18, 19, 20].

These BZO‐based electrolytes with an ABO_3_‐type perovskite structure exhibit high proton conductivity through acceptor (M) doping (BaZr_1‐x_M_x_O_3‐δ_, M = Y, Yb, Gd, etc.) [21, 22, 23]. Partial substitution of **B‐**site with a trivalent acceptor ($M_{\mathrm{Zr}\prime }$) generates the oxygen vacancy ($V_{O}^{••}$) to maintain charge neutrality, which introduces the proton (${\mathrm{OH}}_{O}^{•}$) into the lattice via hydration reaction:

(1) $${2M}_{\mathrm{Zr}}^{\prime }{\;+\;O}_{O}^{X}{\;+\;V}_{O}^{••}+H_{2}O(g)\;\to {\;2M}_{\mathrm{Zr}}^{\prime }{\;+\;2\mathrm{OH}}_{O}^{•}$$

However, BZO‐based electrolytes suffer from several challenges, such as poor sinterability requiring prolonged high‐temperature sintering [24, 25], low grain boundary conductivity [26, 27], barium (Ba) evaporation [25, 28, 29], and dehydration at the elevated temperature [21, 22, 30, 31, 32]. While most of these issues have been improved through sintering aids such as NiO and ZnO, dehydration still causes a significant reduction in proton concentration at operating temperatures (500‐700°C) [24, 33, 34, 35]. For examples, in BaZr_0.8_M_0.2_O_3‐δ_ (M = Y), the proton concentration remains at a maximum of 0.2 per unit cell up to 375°C but declines to 0.14 at 500°C and 0.05 at 700°C [31]. This reduction in proton concentration decreases the number of charge carriers, thereby degrading proton conductivity [30, 31, 36, 37]. Thus, improving hydration properties is essential to further enhance proton conductivity.

Various studies have been conducted to improve hydration performance through acceptor doping. Jin et al. [38], revealed that as dopants make the hydration reaction more exothermic, higher proton concentration can be maintained by suppressing dehydration at elevated temperatures. Han et al. [36], showed that trivalent acceptors such as Y, Yb, and Tm predominantly occupy **B‐**sites over **A‐**sites and exhibit high proton concentration. In addition, Takahashi et al. [39], suggested that acceptors with strong interactions with oxygen prevent the formation of $V_{O}^{••}$ in the hydrated state, effectively suppressing dehydration. Furthermore, Draber et al. [5], identified that acceptors can establish percolation pathways, enabling protons to migrate easily without being trapped at acceptor sites, thereby enhancing hydration performance. Despite these advancements in hydration properties, considerable dehydration still persists at elevated temperatures.

Recently, further enhancement in hydration has been achieved through additional isovalent co‐doping in acceptor‐doped BZO.Zvonareva et al. [40], suggested that additional isovalent Sn co‐doping in Sc‐doped BZO can promote hydration reaction by enhancing $V_{O}^{••}$ mobility through the weaker Sn─O bonding relative to Zr─O bonding. Han et al. [31], showed that isovalent Ce co‐doping in Y‐doped BZO increases dehydration temperature, leading to enhanced proton conductivity. However, these improvements with additional isovalent (N) co‐doping are insufficient to completely mitigate dehydration: the proton concentration decreases to 0.16 at 500°C and further drops to 0.07 at 700°C in BaZr_0.7_M_0.2_N_0.2_O_3‐δ_ (M = Y, N = Ce) [31]. Overcoming this dehydration challenge requires a fundamental understanding of how acceptors affect hydration energetics and how additional isovalent co‐dopants modulate the energetics to improve hydration properties. Thus, a systematic investigation at the atomic scale is necessary to understand the effect of acceptor and isovalent co‐doping on hydration and to identify optimal co‐doping combinations for further enhanced hydration properties.

In this study, first‐principles density functional theory (DFT) calculations are employed to systematically optimize acceptor and isovalent co‐doping for enhancing proton concentration of BZO at elevated temperatures. We first investigate nine trivalent acceptors at the **B‐**site ($M_{Zr}^{\prime }$ = Nd, Pm, Sm, Gd, Y, Ho, Er, Tm, Yb), since their lower valence state facilitates $V_{O}^{••}$ and ${\mathrm{OH}}_{O}^{•}$ formation for hydration. Results suggest that acceptors enhance hydration properties by modifying $V_{O}^{••}$ formation, with $M_{Zr}^{\prime }$ = Yb, Tm showing the lowest *E_hydr_*. Next, we investigate additional isovalent co‐doping at **B‐**site ($N_{Zr}^{\times }$ = Sn, Hf, Pb, Ce, Th) or **A‐**site ($N_{Ba}^{\times }$ = Ca, Sr, Ra) in Yb‐doped BZO to achieve further improvement in hydration properties. Results suggest that $N_{Zr}^{\times }$ = Th and $N_{Ba}^{\times }$ = Ca stabilize ${\mathrm{OH}}_{O}^{•}$ by reducing repulsion with A‐site cations, and this stabilization effect is consistent across different acceptor‐doped BZO system. Based on these results, we suggest promising co‐doping pairs of $M_{Zr}^{\prime }$ = Yb, Tm and $N_{Zr}^{\times }$ = Th or $N_{Ba}^{\times }$ = Ca, which can effectively enhance hydration properties by optimizing $V_{O}^{••}$ formation and ${\mathrm{OH}}_{O}^{•}$ stabilization. We believe that this study will serve as a cornerstone for designing and optimizing the hydration properties of BZO‐based PCOs.

## 2 Results and Discussion

Perovskite BZO doped with trivalent acceptors (M‐BZO) or co‐doped with acceptors and isovalent elements (M,N‐BZO) maintains a cubic ABO_3_ structure under humid conditions at 500–700°C (Table S1) [22, 31, 36, 41, 42, 43, 44]. Therefore, this study considered a $2\sqrt{2}\times 2\sqrt{2}\times 4$ cubic perovskite BZO supercell containing 32 f.u. (160 atoms) (Figure S1a). In the simulation cell, we substituted two acceptors for Zr at **B**‐sites ($M_{Zr}^{\prime }$) and one isovalent dopant for either Zr at **B‐**site ($N_{Zr}^{\times }$) or Ba at **A‐**site ($N_{Ba}^{\times }$). The number for $M_{Zr}^{\prime }$ was set to two for charge neutrality with single $V_{O}^{••}$ or two ${\mathrm{OH}}_{O}^{•}$ participating in the hydration reaction. The cell contains 32 **B‐**sites (**A‐**sites), so the concentration of acceptor yields x = 0.0625 (2/32), and that of isovalent dopant yields y = 0.03125 (1/32), corresponding to BaZr_1‐x_M_x_O_3_ (x = 0.0625) for M‐BZO and BaZr_1‐x‐y_M_x_N_y_O_3_ or Ba_1‐y_N_y_Zr_1‐x_M_x_O_3_ (x = 0.0625, y = 0.03125) for M,N‐BZO. The B‐site preference of trivalent acceptors (M = Yb, Tm, Y, Ho, Gd, Sm, Pm, and Nd) was confirmed by comparing the formation energies of the A‐site substituted and B‐site substituted BZO (Table S2). DFT calculations were performed using the Vienna Ab‐initio Simulation Package (VASP) [45]. The generalized gradient approximation (GGA) based Perdew–Burke–Ernzerhof (PBE) functional was used for the exchange‐correlation, and the projector‐augmented wave (PAW) method was used to describe the electron–ion interaction [46]. The dispersion correction method of Grimme et al. was used to describe properly van der Waals interactions resulting from dynamical correlations between fluctuating charge distributions [47].

**FIGURE 1:**
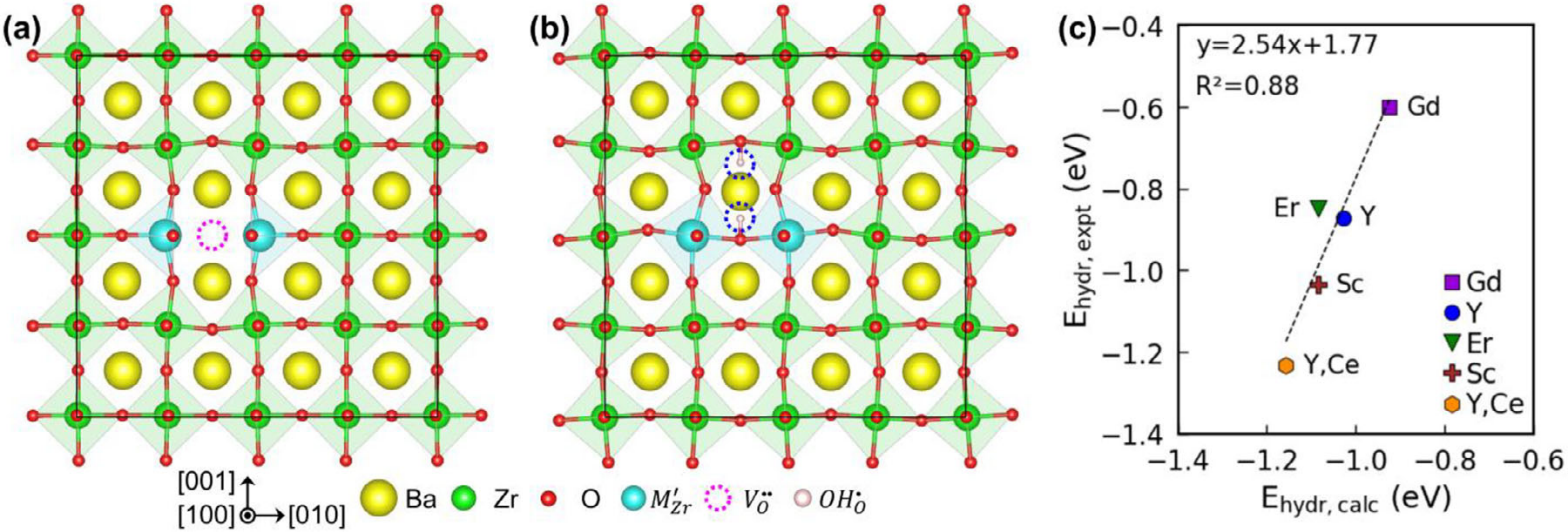
For perovskite BaZrO_3_ with two **B‐**sites substituted by trivalent acceptors ($M_{Zr}^{\prime }$), (a) oxygen‐deficient structure containing one $V_{O}^{••}$ (pink dashed circle) between two $M_{Zr}^{\prime }$ and (b) hydrated structure with two $OH_{O}^{•}$ (blue dashed circle), where one is positioned at the M─O─M site and the other at the Zr─O─Zr site, viewed along the [100] direction. (c) Comparison between calculated (*E*_*hydr*,*calc*_) and experimental hydration energies (*E*_*hydr*, *expt*_) for BaZr_1‐x‐y_M_x_N_y_O_3_ (R^2^ = 0.88). $M_{Zr}^{\prime }$ = Gd, Er, Y, Sc with $N_{Zr}^{\times }$=Zr, and $M_{Zr}^{\prime }$=Y with $N_{Zr}^{\times }$=Ce; x=0.0625, y=0.03125 for *E*_*hydr*,*calc*_ and x=0.2, y=0.2 for *E*_*hydr*, *expt*_ [49, 50].

In the hydration process, a water molecule (H_2_O) reacts with one $V_{O}^{••}$ to form two ${\mathrm{OH}}_{O}^{•}$. Initially, one $V_{O}^{••}$ forms to maintain charge neutrality with two $M_{Zr}^{\prime }$ (Figure 1a). Then, H_2_O reacts with the $V_{O}^{••}$ to dissociate into OH and H. The OH occupies the $V_{O}^{••}$ site, while the H bonds with a neighboring lattice oxygen, finally introducing two ${\mathrm{OH}}_{O}^{•}$ into the lattice (Figure 1b). The hydration reaction heavily depends on the formation of $V_{O}^{••}$ and ${\mathrm{OH}}_{O}^{•}$. Thus, our study focuses on how doping affects the formation of $V_{O}^{••}$ and ${\mathrm{OH}}_{O}^{•}$ to understand the effect of doping on hydration performance.

We first studied energetically favorable configurations of the $V_{O}^{••}$ in M‐BZO and M,N‐BZO. The $V_{O}^{••}$ is positioned closest to two $M_{Zr}^{\prime }$ to maximize the electrostatic interaction between $V_{O}^{••}$ and $M_{Zr}^{\prime }$, resulting in a M‐V_*O*_‐M configuration (Figure 1a; Figure S1b). Experimental measurements have observed the prevalence of first nearest neighbor M‐M pairs [48]. Moreover, it has been reported that the M‐V_*O*_‐M configuration can serve as preferential sites for dehydration at high temperatures [49]. In this study, thus we focused on M‐V_*O*_‐M configuration. In M,N‐BZO, the M‐V_*O*_‐M configuration is also energetically favored, while the $N_{Zr}^{\times }$($N_{Ba}^{\times }$) preferentially occupies the second‐nearest **B‐**site (nearest **A‐**site) from the $V_{O}^{••}$ (Figure S1c,d). This arrangement can be easily understood by the stronger electrostatic attraction between $V_{O}^{••}$ and $M_{Zr}^{\prime }$ than that with $N_{Zr}^{\times }$($N_{Ba}^{\times }$) (Figures S2–S5).

Next, we investigated energetically preferred configurations of 2$OH_{O}^{•}$ in M‐BZO and M,N‐BZO. In M‐BZO, we considered three types of oxygen sites as proton binding sites: M─O─M, M─O─Zr, and Zr─O─Zr. Our results indicate that M─O─M is the most energetically stable site for $OH_{O}^{•}$ formation, followed by the Zr─O─Zr site (Figure S6). Then, we further identified that the energetically favored configuration for 2$OH_{O}^{•}$ consists of one $OH_{O}^{•}$ bonded to the M─O─M site and the other $OH_{O}^{•}$ bonded to the Zr─O─Zr site (Figure 1b; Figure S7). In M,N‐BZO, the proton binding sites in the $N_{Ba}^{\times }$‐doped system are similar to those in M‐BZO, but the $N_{Zr}^{\times }$‐doped system exhibits four different proton binding sites: M─O─M, M─O─Zr, Zr─O─N, and M─O─N. The M─O─M is consistently the most stable site for proton binding, but the relative stability of the second proton binding site varies depending on the ionic radius of the dopant (Figures S8 and S9). Thus, we identified the 2$OH_{O}^{•}$ configuration in which one $OH_{O}^{•}$ is located at the M─O─M site and the other at either the M─O─Zr or Zr─O─Zr site in the $N_{Ba}^{\times }$‐doped system, and at either the M─O─Zr or Zr─O─N site in the $N_{Zr}^{\times }$‐doped system (Figures S10 and S11).

To evaluate the reliability of our hydration energy (*E_hydr_*) calculations, we compared the calculated values (*E*_*hydr*,*calc*_) with experimental data (*E*_*hydr*,*expt*_) for five doping pairs: BaZr_0.6_M_0.2_N_0.2_O_3_ ($M_{Zr}^{\prime }$ = Gd, Er, Y, Sc with $N_{Zr}^{\times }$= Zr, and $M_{Zr}^{\prime }$= Y with $N_{Zr}^{\times }$= Ce) (Figure 1c) [49, 50]. *E_hydr_* was calculated using the following equation:

(2) $$E_{hydr}=-E\left(V_{O}^{••}\right)-E_{H_{2}O\left(g\right)}+E\left(2OH_{O}^{•}\right)$$

where $E(V_{O}^{••})$ and $E(2{\mathrm{OH}}_{O}^{•})$ represent the total energies of the system with one $V_{O}^{••}$ and two ${\mathrm{OH}}_{O}^{•}$, respectively, and $E_{H_{2}O\left(g\right)}$ is the total energy of an isolated water molecule in the gas phase. Although our system considered different doping concentrations from those in experimental studies, *E*_*hydr*,*calc*_ captured the trend of *E*_*hydr*,*expt*_ well (R^2^ = 0.88). The HSE06‐calculated *E*_*hydr*,*calc*_ also reproduce the trend of *E*_*hydr*,*expt*_ well (R^2^ = 0.93, Figure S12) [51]. Based on this validation, we further investigated the effects of nine trivalent acceptors ($M_{Zr}^{\prime }$= Nd, Pm, Sm, Gd, Y, Ho, Er, Tm, Yb) and eight isovalent dopants ($N_{Zr}^{X}$ = Sn, Hf, Pb, Ce, Th, and $N_{Ba}^{X}$ = Ca, Sr, Ra) on hydration behavior.

To understand how trivalent acceptors affect hydration behavior, we calculated defect formation energies (DFE) of $V_{O}^{••}$ and 2$OH_{O}^{•}$ in M‐BZO ($M_{Zr}^{\prime }$= Nd, Pm, Sm, Gd, Y, Ho, Er, Tm, Yb). These two DFE are related to *E_hydr_* through the following equation (details in ESI):

(3) $$E_{hydr}=-DFE\left[V_{O}^{••}]+DFE[2OH_{O}^{•}\right]$$

**FIGURE 2:**
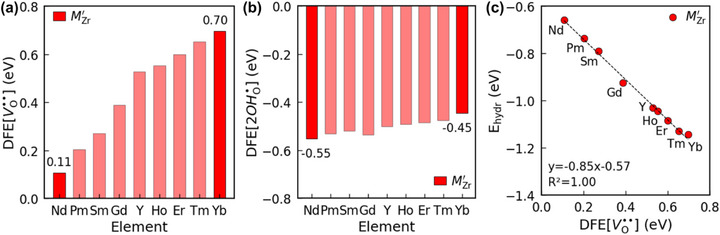
For M‐doped BZO ($M_{Zr}^{\prime }$= Nd, Pm, Sm, Gd, Y, Ho, Er, Tm, Yb), defect formation energy (DFE) of (a) an oxygen vacancy ($V_{O}^{••}$) (DFE[$V_{O}^{••}$]) and (b) two protons ($2OH_{O}^{•}$) (DFE[$2OH_{O}^{•}$]), and (c) correlation between *E_hydr_* and DFE[$V_{O}^{••}$] (R^2^ = 1.00).

This relationship reveals that a higher DFE of $V_{O}^{••}$ and a lower DFE of 2$OH_{O}^{•}$ lead to more negative *E_hydr_*, corresponding to an energetically more favorable hydration reaction. As the acceptor changes from Nd to Yb, DFE of $V_{O}^{••}$ significantly increases from 0.11 to 0.70 eV (Figure 2a), while DFE of 2$OH_{O}^{•}$ shows a relatively smaller increase from ‐0.55 to ‐0.45 eV (Figure 2b). The comparison between DFE of $V_{O}^{••}$ and 2$OH_{O}^{•}$ clearly shows that changes in DFE of $V_{O}^{••}$ (Δ = 0.59 eV) are much more pronounced than those in DFE of 2$OH_{O}^{•}$ (Δ = 0.10 eV). As a result, *E_hydr_* shows a strong negative correlation with DFE of $V_{O}^{••}$ (R^2^ = 1.00): As the acceptors make the formation of $V_{O}^{••}$ less favorable, *E_hydr_* becomes more negative (Figure 2c). Among the acceptors, Yb and Tm exhibit the highest DFE of $V_{O}^{••}$ values (0.70 and 0.66 eV, respectively) and the most negative *E_hydr_* (‐1.14 and ‐1.13 eV, respectively). The decreased *E_hydr_* with the increased DFE of $V_{O}^{••}$ can be understood as a reduction in dehydration. As DFE of $V_{O}^{••}$ increases, the probability of O desorption decreases. As a result, dehydration is reduced, and hydration is more stabilized.

**FIGURE 3:**
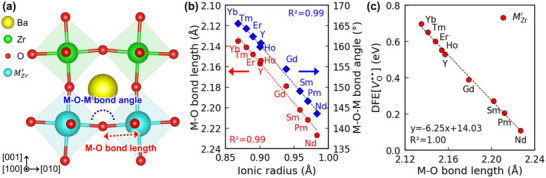
(a) Local structure around two adjacent $M_{Zr}^{\prime }$ in stoichiometric M‐doped BZO, viewed along the [100] direction. For stoichiometric M‐doped BZO ($M_{Zr}^{\prime }$= Nd, Pm, Sm, Gd, Y, Ho, Er, Tm, Yb), (b) M─O bond length (red circles, left axis) (R^2^ = 0.99) and M─O─M bond angle (blue diamonds, right axis) (R^2^ = 0.99) as a function of ionic radius of acceptors, and (c) correlation between M─O bond length and DFE[$V_{O}^{••}$] (R^2^ = 1.00).

To understand how acceptors influence $V_{O}^{••}$ formation, we investigated the M─O─M local structure in stoichiometric M‐BZO (Figure 3a). Our results show that as the ionic radius decreases from 0.983 Å (Nd) to 0.868 Å (Yb), the M─O bond length decreases from 2.227 Å (Nd) to 2.135 Å (Yb), accompanied by an increase in the M─O─M bond angle from 143.6° (Nd) to 165.5° (Yb) (Figure 3b). This observed trend is reasonable, because as the ionic radius mismatch between acceptors and Zr (0.72 Å) reduces, the local structure approaches the cubic perovskite of BZO (Zr─O bond length: 2.11 Å, Zr─O─Zr bond angle: 180°). As the M─O bond length decreases, the DFE of $V_{O}^{••}$ increases from 0.10 eV (Nd) to 0.69 eV (Yb) (Figure 3c). The shorter M─O bond length increases bond strength between the acceptor and oxygen atom, making it energetically less favorable to form $V_{O}^{••}$ by removing O. Thus, acceptors can change $V_{O}^{••}$ formation by modulating M─O bond strength, depending on ionic size.

**FIGURE 4:**
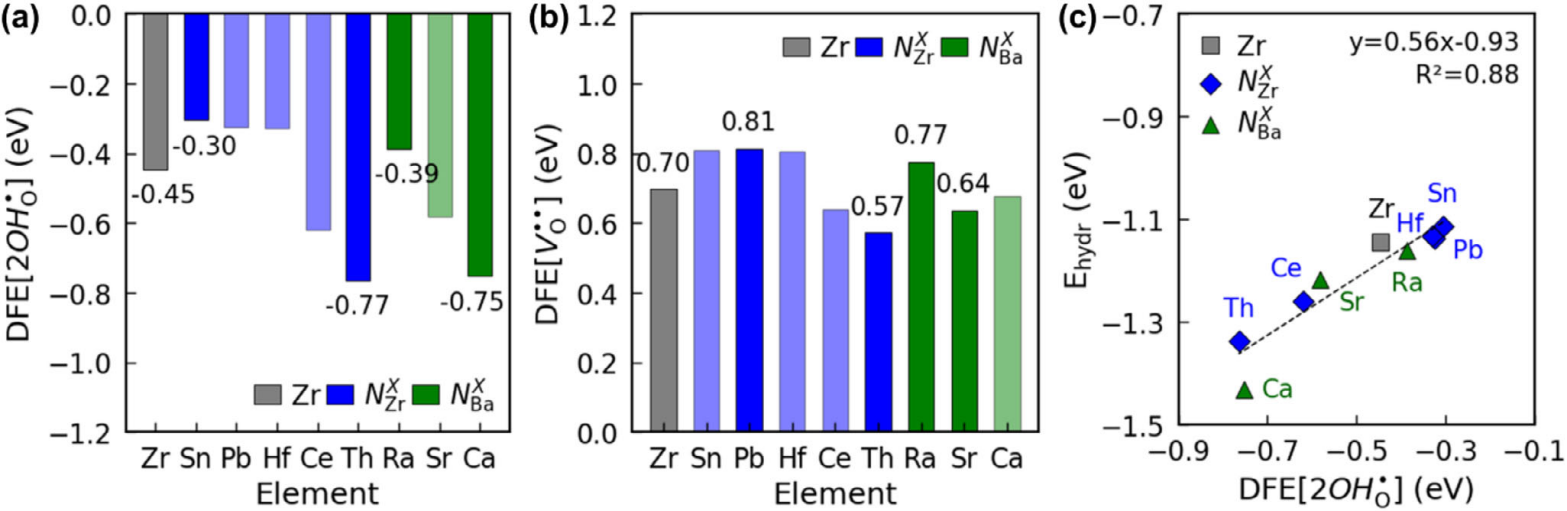
For Yb, N‐BZO ($N_{Zr}^{\times }$= Zr, Sn, Pb, Hf, Ce, Th, and $N_{Ba}^{\times }$= Ra, Sr, Ca), (a) DFE[$2OH_{O}^{•}$], (b) DFE[$V_{O}^{••}$], and (c) correlation between *E_hydr_* and DFE[$2OH_{O}^{•}$] (R^2^ = 0.88).

Next, we focused on the effect of isovalent dopants to understand how isovalent co‐doping affects the hydration behavior. For this study, Yb was selected as the fixed acceptor dopant because it exhibits the most negative *E_hydr_*, and we considered five isovalent dopants for Zr‐site substitution ($N_{Zr}^{X}$ = Sn, Hf, Pb, Ce, Th) and three isovalent dopants for Ba‐site substitution ($N_{Ba}^{X}$ = Ca, Sr, Ra). To assess the effect of isovalent co‐doping on *E_hydr_*, we calculated the DFE of $V_{O}^{••}$ and 2$OH_{O}^{•}$ in Yb,N‐BZO. Our results show that isovalent doping significantly influences 2$OH_{O}^{•}$ formation: DFE of 2$OH_{O}^{•}$ varies from ‐0.30 eV (Sn) to ‐0.77 eV (Th) for $N_{Zr}^{\times }$ and from ‐0.39 eV (Ra) to ‐0.75 eV (Ca) for $N_{Ba}^{\times }$ (Figure 4a). In contrast, DFE of $V_{O}^{••}$ shows relatively moderate variation, decreasing from 0.81 eV (Pb) to 0.57 eV (Th) for $N_{Zr}^{\times }$ and from 0.77 eV (Ra) to 0.64 eV (Sr) for $N_{Ba}^{\times }$ (Figure 4b). Correlation analysis indicates that while DFE of both $V_{O}^{••}$ and 2$OH_{O}^{•}$ decrease with isovalent doping, the change in DFE of 2$OH_{O}^{•}$ (Δ  =  ‐0.47 eV) is nearly twice that of $V_{O}^{••}$ (Δ  =  ‐0.24 eV). This result suggests that, unlike acceptors, isovalent doping enhances *E_hydr_* primarily by stabilizing 2$OH_{O}^{•}$ formation (Figure 4c, R^2^ = 0.88). Especially, $N_{Zr}^{X}$= Th and $N_{Ba}^{X}$= Ca, which stabilize 2$OH_{O}^{•}$ with the lowest DFE of 2$OH_{O}^{•}$ (‐0.77 and ‐0.75 eV, respectively), exhibit the most negative hydration energies (‐1.34 eV and ‐1.43 eV, respectively).

**FIGURE 5:**
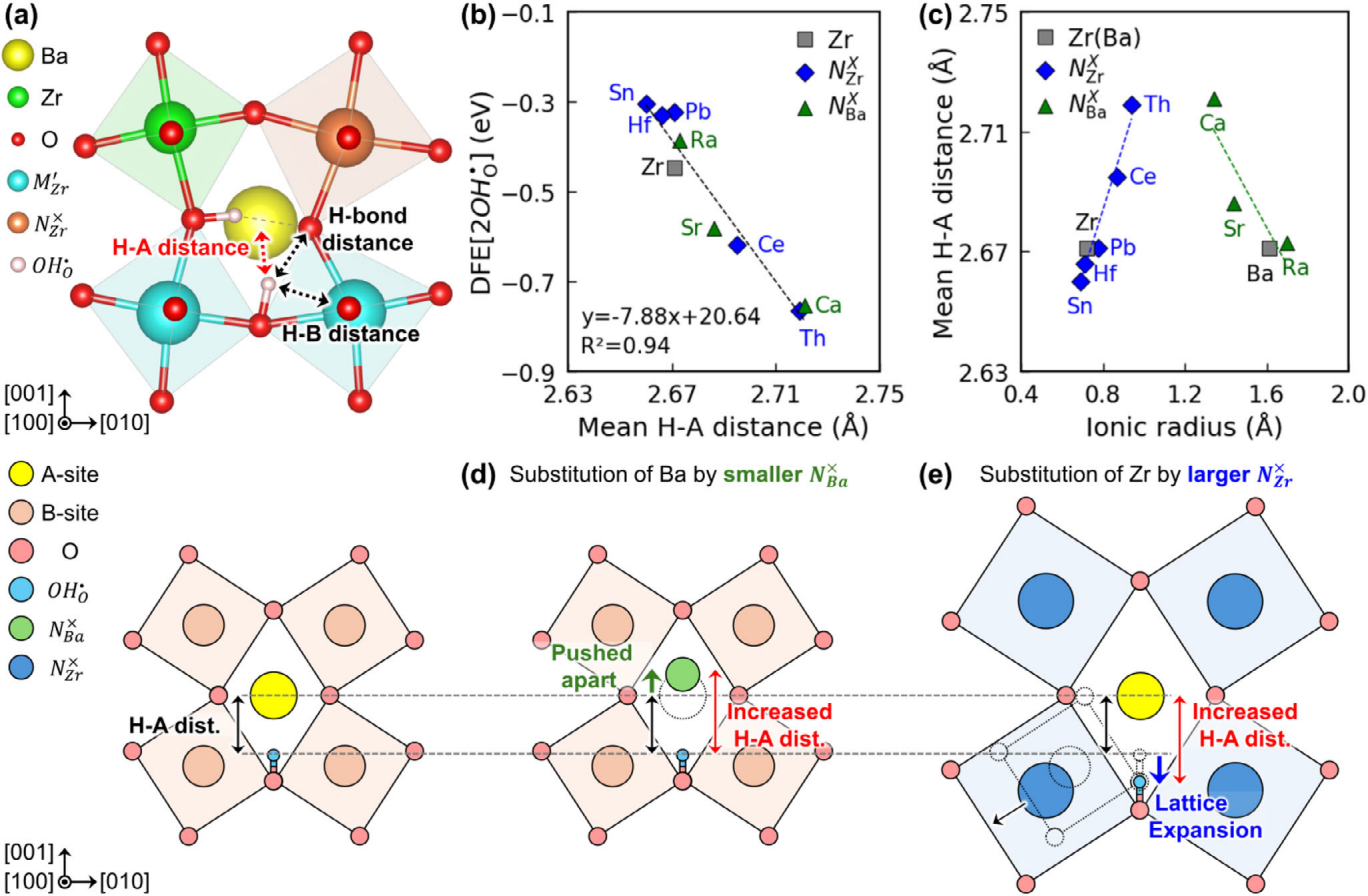
(a) Local structure of $M_{Zr}^{\prime }$ and $N_{Zr}^{\times }$ co‐doped BZO, viewed along the [100] direction. Three structural parameters for one $OH_{O}^{•}$ were defined: hydrogen bond distance of $OH_{O}^{•}$ (H‐bond distance), mean distance between $OH_{O}^{•}$ and its two nearest **A‐** or **B‐**site cations (H‐A distance or H‐B distance). Since two $OH_{O}^{•}$ exist in the hydrated structure, the mean values over both defects were used. For Yb, N‐BZO ($N_{Zr}^{\times }$= Sn, Hf, Zr, Pb, Ce, Th, and $N_{Ba}^{\times }$= Ra, Sr, Ca), (b) correlation between DFE[$2OH_{O}^{•}$] and mean H‐A distance (R^2^ = 0.94), and (c) mean H‐A distance as a function of ionic radius of isovalent dopant. Schematic illustrations of increased H‐A distance (d) by A‐site substitution with smaller cations and (e) by B‐site substitution with larger cations, respectively.

To understand how different isovalent doping affects 2$OH_{O}^{•}$ formation, we investigated three different structural parameters: the mean hydrogen bond distance of two $OH_{O}^{•}$ (mean H‐bond distance), and the mean distance from two $OH_{O}^{•}$ to their respective two nearest **A‐** or **B‐**site cations (mean H‐A distance or mea n H‐B distance) in ABO_3_‐type perovskite structure (Figure 5a). Our calculations revealed that the relationship of H─bond distance or mean H‐B distance with DFE of 2$OH_{O}^{•}$ is unclear (Figure S13). In contrast, mean H‐A distance shows a strong correlation with DFE of 2$OH_{O}^{•}$ (R^2^ = 0.94) (Figure 5b); as mean H‐A distance increases, DFE of 2$OH_{O}^{•}$ decreases. This indicates that isovalent dopants can stabilize $OH_{O}^{•}$ formation by reducing the electrostatic repulsion between $OH_{O}^{•}$ and A‐site cations.

We further investigated the relationship between dopant ionic radius and H‐A distance to understand how this structural change occurs (Figure 5c). For **A‐**site substitution (green triangle), replacing Ba with smaller cations creates more free space at the **A‐**site. This expanded free space allows the proton and A‐site cation to be pushed apart by electrostatic repulsion, thereby increasing the H‐A distance (Figure 5d). For **B‐**site substitution (blue diamond), replacing Zr with larger cations increases the system volume, expanding the free space at the A‐site. Similar to the case for **A‐**site substitution, this expanded free space increases the distance between $OH_{O}^{•}$ and **A‐**site cation through electrostatic repulsion. As the dopant size increases, **A‐**site cation is further pushed out, leading to larger H‐A distances (Figure 5e; Figure S14). Through these different mechanisms, the H‐A distance can be increased by both **A‐**site and **B‐**site isovalent dopants, and thereby the stability of 2$OH_{O}^{•}$ is enhanced. These results suggest that additional isovalent co‐doping can modulate 2$OH_{O}^{•}$ formation through the ionic size effect, thus complementing acceptor doping for enhanced hydration behavior. In summary, while acceptors can effectively control $V_{O}^{••}$ formation by changing the M─O bonds length, isovalent co‐doping can stabilize 2$OH_{O}^{•}$ by modulating the electrostatic repulsion through the H‐A distances. Additional calculations with clustered dopant configurations, finite‐temperature effects, and the HSE06 method verified that the synergistic effects of co‐doping on hydration behavior are robust regardless of increased dopant‐dopant interactions, elevated operating temperature, and the choice of exchange‐correlation functional (Figures S15–S18).

**FIGURE 6:**
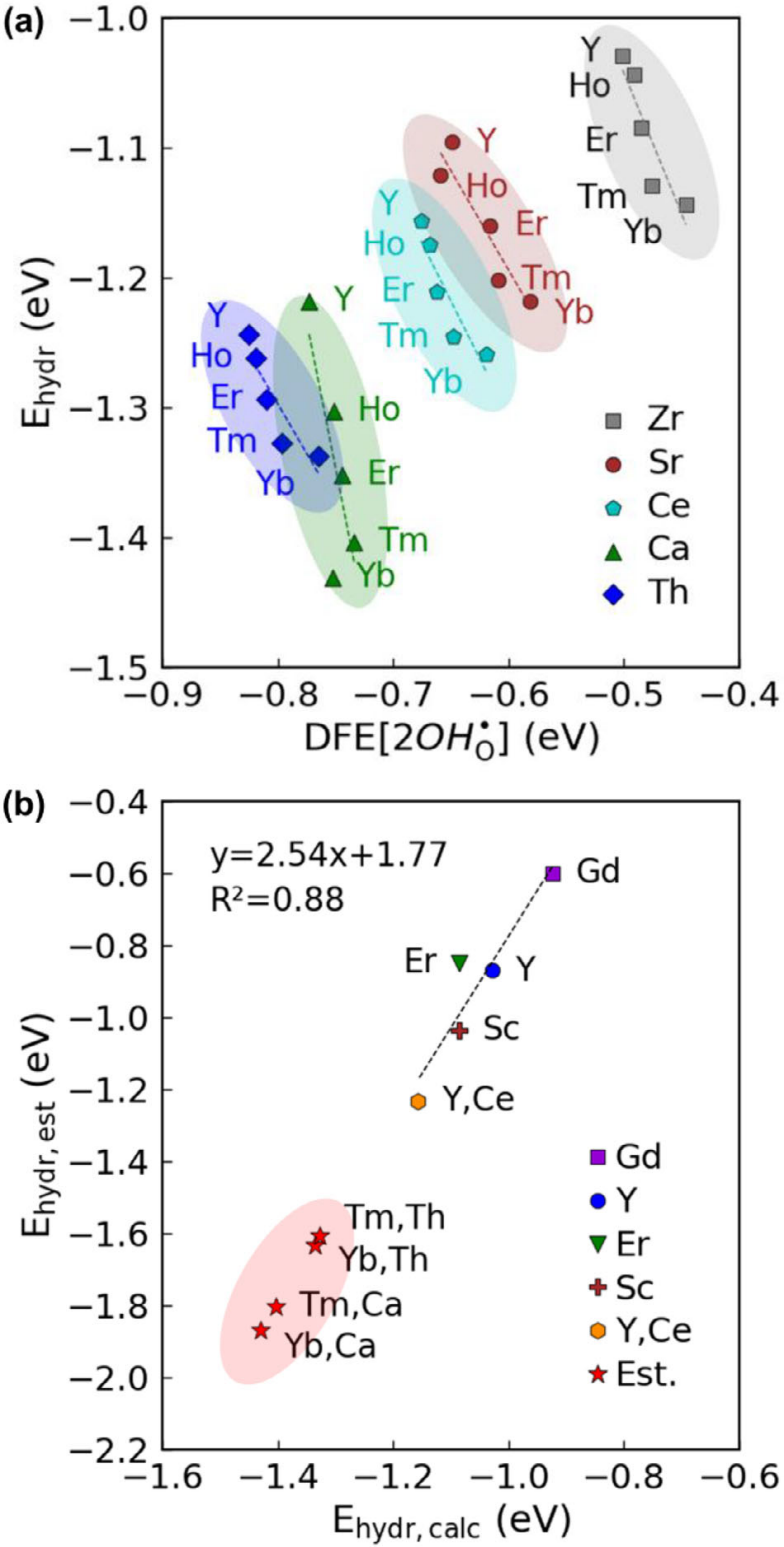
(a) Correlation between *E_hydr_* and DFE[$2OH_{O}^{•}$] for M,N‐BZO ($M_{Zr}^{\prime }$= Y, Ho, Er, Tm, Yb and $N_{Zr}^{\times }$= Ce, Th or $N_{Ba}^{\times }$= Sr, Ca), and (b) estimated *E_hydr_* (red stars) for M,N‐BZO ($M_{Zr}^{\prime }$= Tm, Yb and $N_{Zr}^{\times }$= Th or $N_{Ba}^{\times }$= Ca) based on the correlation between calculated and experimental *E_hydr_* established in Figure 1c.

Based on our calculations results, combining acceptor ($M_{Zr}^{\prime }$= Yb) with isovalent dopants with low DFE of 2$OH_{O}^{•}$ ($N_{Zr}^{X}$= Th and $N_{Ba}^{X}$= Ca) can enhance hydration properties effectively. To identify whether this isovalent co‐doping effect is generally applicable beyond the specific case of acceptor Yb, we extended our study to other trivalent acceptors ($M_{Zr}^{\prime }$ = Tm, Er, Ho, Y), and further investigate the isovalent co‐doping effect ($N_{Zr}^{\times }$ = Ce, Th and $N_{Ba}^{\times }$ = Ca, Sr). The results show that isovalent co‐doping consistently reduces *E_hydr_* by stabilizing $OH_{O}^{•}$ across different M‐BZO (Figure 6a; Figure S19). Notably, $N_{Ba}^{\times }$= Ca and $N_{Zr}^{\times }$= Th were the most effective in stabilizing $OH_{O}^{•}$. This confirmed that the effect of isovalent co‐doping, previously observed with Yb, is robust across different trivalent acceptors.

Furthermore, using the correlation between the calculated and experimental *E_hydr_*, established in Figure 1c, we estimated the experimental *E_hydr_* for four promising co‐doping pairs: (i) $M_{Zr}^{\prime }$= Tm and $N_{Zr}^{\times }$= Th (‐152.6 kJ/mol), (ii) $M_{Zr}^{\prime }$= Yb and $N_{Zr}^{\times }$= Th (‐155 kJ/mol), (iii) $M_{Zr}^{\prime }$= Tm and $N_{Ba}^{\times }$= Ca (‐169.1 kJ/mol), and (iv) $M_{Zr}^{\prime }$= Yb and $N_{Ba}^{\times }$= Ca (‐176.2 kJ/mol). These predicted values indicate that the proposed co‐doping pairs (red stars in Figure 6b) can enhance hydration performance compared to a conventional pair of $M_{Zr}^{\prime }$= Y and $N_{Zr}^{\times }$= Ce (‐119 kJ/mol). These results suggest that modulating $V_{O}^{••}$ formation through appropriate acceptor doping ($M_{Zr}^{\prime }$= Tm and Yb) and stabilizing 2$OH_{O}^{•}$ formation via isovalent co‐doping ($N_{Zr}^{X}$= Th and $N_{Ba}^{X}$= Ca) can be an effective way to improve hydration performance in BZO systems (Table S4). As a result, our study identified the effectiveness and the general applicability of acceptor and isovalent co‐doping in tailoring hydration behavior. This fundamental understanding of co‐doping effects can provide practical guidelines for improving hydration behavior in BZO‐based PCOs.

To further investigate the practical viability of our proposed co‐doping pairs (Tm‐Th, Yb‐Th, Tm‐Ca, and Yb‐Ca) as protonconducting electrolytes, we evaluated their chemical stability and proton migration barrier. For chemical stability against CO_2_ and H_2_O, we calculated the carbonation (Δ*E_carbonation_*) and hydroxylation reaction energies (Δ*E_hydroxylation_*) (Table S5). BaCeO_3_ (BCO) exhibits Δ *E_carbonation_* = ‐2.24 eV and Δ *E_hydroxylation_* = ‐1.21 eV, whereas the proposed pairs show Δ *E_carbonation_* = ‐1.71, ‐1.70, ‐1.67, and ‐1.66 eV, and Δ *E_hydroxylation_* = ‐0.68, ‐0.68, ‐0.66, and ‐0.65 eV for Tm‐Th, Yb‐Th, Tm‐Ca, and Yb‐Ca, respectively. The differences relative to BCO are 0.53 to 0.58 eV for carbonation and 0.53 to 0.56 eV for hydroxylation, confirming that the co‐doping pairs preserve the inherent chemical stability of BZO well. For proton migration, we calculated the energy barrier (*E_migr_*) for proton transfer from one oxygen site in one octahedron to other oxygen site in the neighboring octahedron along the inter‐octahedral diffusion pathway [52, 53, 54, 55]. The Y‐Ce pair exhibits *E_migr_* = 0.15 eV, whereas the proposed pairs show *E_migr_* = 0.16, 0.17, 0.24, and 0.26 eV for Tm‐Th, Yb‐Th, Tm‐Ca, and Yb‐Ca, respectively (Figures S20 and S21). The increases relative to Y‐Ce are modest (Δ*E_migr_* = 0.01, 0.02, 0.09, and 0.11 eV), whereas the hydration energy gains are substantially larger: the Y‐Ce pair exhibits *E_hydr_* = ‐1.16 eV, whereas the proposed pairs show *E_hydr_* = ‐1.33, ‐1.34, ‐1.40, and ‐1.43 eV for Tm‐Th, Yb‐Th, Tm‐Ca, and Yb‐Ca, respectively, corresponding to Δ *E_hydr_* = ‐0.17, ‐0.18, ‐0.25, and ‐0.27 eV relative to Y‐Ce. Thus, the Δ*E_migr_* of 0.01 to 0.11 eV is marginal, whereas the Δ*E_hydr_* of ‐0.17 to ‐0.27 eV is significantly larger. Moreover, enhancing hydration performance becomes more critical at practical operating temperatures (500–700°C). At elevated temperatures, proton diffusion becomes more facile as the increased thermal energy enables protons to readily overcome migration barriers. In contrast, dehydration becomes increasingly severe because the entropic contribution (TΔS) of the H_2_O vapor grows with temperature, making dehydration the dominant performance‐limiting factor. Therefore, the modest migration penalty is far outweighed by the substantial hydration energy gains. Combined with the excellent chemical stability, the proposed co‐doping pairs can be promising candidates for high‐performance proton‐conducting electrolytes at elevated operating temperatures.

In practice, dopant selection may further depend on application‐specific constraints. While Th exhibits the largest improvement in *E_hydr_* among the investigated B‐site isovalent dopants, its radioactivity may impose practical limitations under certain operational conditions. Nevertheless, the underlying design principle—enhancing hydration by increasing the H–A distance through larger B‐site dopants—remains broadly applicable; for example, Ce may serve as a viable non‐radioactive alternative with a larger ionic radius (0.87 Å) than Zr (0.72 Å). This enables the co‐doping pair to be readily tailored to practical requirements while preserving the key design principle.

## 3 Conclusion

In this study, we used DFT calculations to understand the effects of acceptor and isovalent co‐doping on the hydration performance of BZO. Our results show that acceptor dopants, particularly Yb and Tm, can enhance hydration behavior by reducing $V_{O}^{••}$ formation through M─O bond length. In addition, we found that isovalent co‐doping can further enhance hydration by stabilizing 2$OH_{O}^{•}$, which is associated with an increased distance between the **A‐**site cation and the $OH_{O}^{•}$. The effect of isovalent co‐doping was consistent across various acceptor‐doped BZO systems, and Th and Ca exhibited the strongest stabilization effect on 2$OH_{O}^{•}$. As a result, we suggest that co‐doping is effective to enhance hydration behavior by combining acceptor doping to modulate $V_{O}^{••}$ formation with additional isovalent doping to stabilize 2$OH_{O}^{•}$. Especially, four co‐doping pairs (Yb–Ca, Yb–Th, Tm–Ca, and Tm–Th) were estimated as promising candidates, exhibiting significantly more negative *E_hydr_* than the conventional Y–Ce pair.

The temperature dependence of proton concentration in PCOs could impede their application as electrolytes in protonic ceramic fuel cell and electrolysis cell. Through quantitative evaluation of defect stability, we show that optimizing acceptor and isovalent co‐doping can lead to further improvement in hydration properties. Especially, the synergistic interactions between acceptor Yb, Tm, and isovalent dopant Th,Ca can effectively address the temperature sensitivity of proton concentration. Our calculation results provide co‐doping combination guidelines for designing highly effective proton‐conducting electrolytes at elevated temperatures.

## Conflicts of Interest

The authors declare no conflicts of interest.

## Supporting information




**Supporting File**: advs74833‐sup‐0001‐SuppMat.docx.

## Data Availability

The data that support the findings of this study are available in the supplementary material of this article.
