# Supplementary material for: Synergistic Interplay of Acceptor and Isovalent Co‐Doping on BaZrO3‐Based Proton Conducting Oxides: A First‐Principles Study
Source: Adv Sci (Weinh). 2026 Apr 3;13(30):e24188. doi: 10.1002/advs.202524188 (PMC13248769; doi:10.1002/advs.202524188)
Supplement: Supplementary file 1 — Supporting File: advs74833‐sup‐0001‐SuppMat.docx. [file ADVS-13-e24188-s001.docx]

Synergistic Interplay of Acceptor and Isovalent Co-doping on BaZrO_3_-based Proton Conducting Oxides: A First-Principles Study

Yonghun Shin,^a^ Kyung-Yeon Doh,^a^ June Ho Lee,^a^ Shin Hyun Kim,^a^ and Donghwa Lee^*a,b,c^

*^a^ Department of Materials Science and Engineering, Pohang University of Science and Technology, Pohang 37673, South Korea*

*^b^ Division of Advanced Materials Science, Pohang University of Science and Technology, Pohang 37673, South Korea*

*^c^Institute for Convergence Research and Education in Advanced Technology (I_CREATE), Yonsei University, Incheon 21983, Republic of Korea*

*Corresponding author: donghwa96@postech.ac.kr

**A. Computational method**

DFT calculations were performed to understand how acceptor doping and additional isovalent doping affect the hydration performance of BaZrO_3_ (BZO) at high temperature using the Vienna Ab-initio Simulation Package (VASP).[1] The generalized gradient approximation (GGA) based Perdew–Burke–Ernzerhof (PBE) functional for the exchange-correlation and the projector-augmented wave (PAW) method were used to describe the electron–ion interaction.[2] The PAW potentials with valence configurations 5s^2^5p^6^6s^2^ for Ba, 4s^2^4p^6^5s^1^4d^3^ for Zr, 2s^2^2p^4^ for O, 1s^1^ for H, 4s^2^4p^6^5s^1^4d^2^ for Y, 5p^6^6s^2^5d^1^ for Yb, Tm, Er, Ho, Gd, 5s^2^5p^6^6s^1^5d^2^ for Sm, Pm, Nd, 5s^2^5p^2^ for Sn, 5s^2^5p^6^6s^0^5d^4^ for Hf, 6s^2^6p^2^ for Pb, 5s^2^5p^6^6s^2^5d^1^4f^1^ for Ce, 6s^2^6p^6^7s^2^6d^1^5f^1^ for Th, 3s^2^3p^6^4s^2^ for Ca, 4s^2^4p^6^5s^2^ for Sr were used to describe the valence electrons. The plane wave was expanded to a cut-off energy of 520 eV. The structures were fully relaxed until the Hellmann–Feynman force on each atom was below 0.01 eV/Å with an energy convergence criterion of 10^-6^ eV. The dispersion correction method of Grimme et al.[3] was used to describe properly van der Waals interactions resulting from dynamical correlations between fluctuating charge distributions.

Calculations were performed using a $2\sqrt{2}\times2\sqrt{2}\times4$ cubic perovskite BZO supercell containing 32 f.u. (160 atoms), since the cubic ABO₃ structure is preserved in both acceptor-doped (M-BZO) and acceptor-isovalent co-doped (M, N-BZO) systems under various temperature and atmosphere conditions (Table S1).[4-10] 9 different trivalent acceptors at Zr-site ($M_{Zr}^{'}$=Yb, Tm, Er, Y, Ho, Gd, Sm, Pm, Nd) and 6 different isovalent dopants at Zr site ($N_{Zr}^{\times}$=Sn, Hf, Pb, Ce, Th) and 3 different isovalent dopants at Ba site ($N_{Ba}^{\times}$=Ca, Sr, Ra) were considered to evaluate the effect of the dopants on hydration behavior of BZO.

For five different doping pairs ($M_{Zr}^{'}$ = Gd, Er, Y, Sc with $N_{Zr}^{\times}$=Zr, and $M_{Zr}^{'}$=Y with $N_{Zr}^{\times}$=Ce), calculated hydration energies ($E_{hydr}$) with full relaxation showed better correlation with experimental values (R^2^=0.87) compared to those with fixed cell parameters (R^2^ = 0.48) (Fig. S22a,b).[11-12] In addition, our test calculations revealed negligible differences in $E_{hydr}$ between spin-polarized and non-spin-polarized calculations for both M-BZO and M,N-BZO systems (Fig. S23a, b). Therefore, in this study, $E_{hydr}$ and defect formation energies (DFE) of one oxygen vacancy ($\text{V}_{\text{O}}^{\text{}\text{}}$) and two interstitial protons (2${OH}_{O}$) were calculated under full relaxation and non-spin-polarized condition.

**B. Oxygen deficient and hydrated structures of M-BZO and M, N-BZO**

In our simulation cell, we considered two $M_{Zr}^{'}$ and one $N_{Zr}^{\times}$ ($N_{Ba}^{\times}$) to investigate their effects on hydration properties. The number of $M_{Zr}^{'}$ was set to two for charge neutrality with $\text{V}_{\text{O}}^{\text{}\text{}}$ and 2${OH}_{O}$ involved in hydration reaction.

To determine the spatial configuration of dopants and $\text{V}_{\text{O}}^{\text{}\text{}}$, we first compared $\text{V}_{\text{O}}^{\text{}\text{}}$ binding energies with single $M_{Zr}^{'}$ (Fig. S2a), and single $N_{Zr}^{\times}$ ($N_{Ba}^{\times}$) (Fig. S2b, c). These binding energies were calculated using the Equation S1 and S2, respectively:

for BZO with $M_{Zr}^{'}$, $BE\left[ \text{V}_{\text{O}}^{\text{}\text{}} \right]=E\left( M_{Zr}^{'}+V_{O} \right)+E\left( BZO \right)-E\left( M_{Zr}^{'} \right)-E\left( V_{O} \right)+E_{corr}$ (Eq. S1)

for BZO with $N_{Zr}^{\times}$ ($N_{Ba}^{\times}$), $BE\left[ \text{V}_{\text{O}}^{\text{}\text{}} \right]=E\left( N^{\times}+V_{O} \right)+E\left( BZO \right)-E\left( N^{\times} \right)-E\left( V_{O} \right)+E_{corr}$ (Eq. S2)

where $BE\left[ \text{V}_{\text{O}}^{\text{}\text{}} \right]$ for BZO with $M_{Zr}^{'}$ and BZO with $N_{Zr}^{\times}$ ($N_{Ba}^{\times}$) are $\text{V}_{\text{O}}^{\text{}\text{}}$ binding energies with $M_{Zr}^{'}$ and $N_{Zr}^{\times}$ ($N_{Ba}^{\times}$), respectively; $E\left( M_{Zr}^{'}+V_{O} \right)$, $E\left( N^{\times}+V_{O} \right)$, and $E\left( V_{O} \right)$ are total energies of oxygen deficient structures for $M_{Zr}^{'}$-doped, $N_{Zr}^{\times}$ ($N_{Ba}^{\times}$)-doped, and pristine BZO, respectively; $E\left( M_{Zr}^{'} \right)$, $E\left( N^{\times} \right)$, and $E\left( BZO \right)$ are total energies of stoichiometric structures for $M_{Zr}^{'}$-doped, $N_{Zr}^{\times}$ ($N_{Ba}^{\times}$)-doped, and pristine BZO, respectively; and $E_{corr}$ is the energy correction term for electrostatic interactions between periodic images of the charged defects. $M_{Zr}^{'}$ showed stronger binding (-0.60 to -0.25 eV for Nd to Yb) compared to $N_{Zr}^{\times}$ (-0.20 to 0.14 eV for Ce to Hf) and $N_{Ba}^{\times}$ (-0.13 to -0.03 eV for Ca to Ra) (Fig. S2d). Based on these stronger binding tendencies, we first positioned two $M_{Zr}^{'}$ around $\text{V}_{\text{O}}^{\text{}\text{}}$, leading to M-$\text{V}_{\text{O}}^{\text{}\text{}}$-M cluster. Further calculations showed that binding energy becomes more negative as M-$\text{V}_{\text{O}}^{\text{}\text{}}$ distance decreases (Fig. S3a, b), supporting the stability of M-$\text{V}_{\text{O}}^{\text{}\text{}}$-M cluster in M-BZO. Subsequently, we positioned one $N_{Zr}^{\times}$ ($N_{Ba}^{\times}$) at the second-nearest Zr-site (the nearest Ba-site) to $\text{V}_{\text{O}}^{\text{}\text{}}$, completing the oxygen deficient structures of M, N-BZO.

To consider the energetically preferred configurations of 2${OH}_{O}$, we first investigated single ${OH}_{O}$ binding energies at different oxygen sites. ${OH}_{O}$ binding energies in M-BZO and M,N-BZO were calculated using the Equation S3 and S4, respectively:

for M-BZO, $BE\left[ {OH}_{O} \right]=E\left( 2M_{Zr}^{'}+{OH}_{O} \right)+E\left( BZO \right)-E\left( 2M_{Zr}^{'} \right)-E\left( {OH}_{O} \right)+E_{corr}$ (Eq. S3)

for M,N-BZO,$BE\left[ {OH}_{O} \right]=E\left( 2M_{Zr}^{'}+N^{\times}+{OH}_{O} \right)+E\left( BZO \right)-E\left( 2M_{Zr}^{'}+N^{\times} \right)-E\left( {OH}_{O} \right)+E_{corr}$ (Eq. S4)

where $BE\left[ {OH}_{O} \right]$ for M-BZO and M,N-BZO are ${OH}_{O}$ binding energies with $2M_{Zr}^{'}$ and $2M_{Zr}^{'}+N_{Zr}^{\times}$ ($N_{Ba}^{\times}$), respectively; $E\left( 2M_{Zr}^{'}+{OH}_{O} \right)$, $E\left( 2M_{Zr}^{'}+N^{\times}+{OH}_{O} \right)$, and $E\left( {OH}_{O} \right)$ are total energies of M-BZO, M,N-BZO, and pristine BZO containing one ${OH}_{O}$, respectively; $E\left( 2M_{Zr}^{'} \right)$, $E\left( 2M_{Zr}^{'}+N^{\times} \right)$, and $E\left( BZO \right)$ are total energies of M-BZO, M,N-BZO, and pristine BZO, respectively; and $E_{corr}$ is the energy correction term for electrostatic interactions between periodic images of the charged defects. For M-BZO, we calculated ${OH}_{O}$ binding energies at three oxygen sites: within M-O-M (Fig. S6a), M-O-Zr (Fig. S6b), and Zr-O-Zr (Fig. S6c). The M-O-M site showed the strongest binding with ${OH}_{O}$ (Fig. S6d). Based on this result, we considered two different configurations of 2${OH}_{O}$ by placing one ${OH}_{O}$ at M-O-M site and the other at either M-O-Zr or Zr-O-Zr sites (Fig. S7). For M, N-BZO systems, the isovalent dopant can introduce additional binding sites. With $N_{Ba}^{\times}$, we considered three sites: M-O-M (Fig. S8a), M-O-Zr (Fig. S8b), and Zr-O-Zr (Fig. S8c). With $N_{Zr}^{\times}$, we examined ${OH}_{O}$ binding at four sites: M-O-M (Fig. S9a), M-O-Zr (Fig. S9b), Zr-O-N (Fig. S9c), and M-O-N (Fig. S9d). Our results revealed that M-O-M remains the most favorable site regardless of isovalent dopant type (Fig. S8d, S9e). Therefore, we placed one ${OH}_{O}$ at M-O-M and considered different sites for the second ${OH}_{O}$: either M-O-Zr, Zr-O-N, or M-O-N in $N_{Zr}^{\times}$-doped systems, and either M-O-Zr or Zr-O-Zr in $N_{Ba}^{\times}$-doped systems. As a result, we investigated two different hydrated structures for M-BZO, three for M, N-BZO with $N_{Zr}^{\times}$, and two for M, N-BZO with $N_{Ba}^{\times}$.

**C. Defect formation energies (DFE) of** $\text{V}_{\text{O}}^{\text{}\text{}}$ **and 2**$\boldsymbol{OH}_{\boldsymbol{O}}$ **in M-BZO and M, N-BZO**

$E_{hydr}$ for M-BZO and M,N-BZO were calculated as the energy difference between oxygen-deficient and hydrated structures, considering the energy of water molecule, using Equation S5 and S6, respectively:

for M-BZO, $E_{hydr}=-E\left( 2M_{Zr}^{'}+\text{V}_{\text{O}}^{\text{}\text{}} \right)-E\left( H_{2}O\left( g \right) \right)+E(2M_{Zr}^{'}+$2${OH}_{O})$ (Eq. S5)

for M,N-BZO, $E_{hydr}=-E\left( 2M_{Zr}^{'}+N^{\times}+\text{V}_{\text{O}}^{\text{}\text{}} \right)-E\left( H_{2}O\left( g \right) \right)+E(2M_{Zr}^{'}+N^{\times}+2{OH}_{O})$ (Eq. S6)

where $E\left( 2M_{Zr}^{'}+\text{V}_{\text{O}}^{\text{}\text{}} \right)$ and $E\left( 2M_{Zr}^{'}+N^{\times}+\text{V}_{\text{O}}^{\text{}\text{}} \right)$ are total energies of M-BZO and M,N-BZO with one $\text{V}_{\text{O}}^{\text{}\text{}}$, respectively; $E(2M_{Zr}^{'}+$2${OH}_{O})$ and $E(2M_{Zr}^{'}+N^{\times}+2{OH}_{O})$ are total energies of M-BZO and M,N-BZO with two ${OH}_{O}$, respectively; and $E\left( H_{2}O\left( g \right) \right)$ is total energy of a water molecule. $E_{hydr}$ becomes more negative when $\text{V}_{\text{O}}^{\text{}\text{}}$ are less stable or when ${OH}_{O}$ are more stable, thereby suppressing dehydration. To understand the effect of dopants on $E_{hydr}$, we analyzed their influence on the stability of $\text{V}_{\text{O}}^{\text{}\text{}}$ and ${OH}_{O}$ through defect formation energies (DFE). The DFE of $\text{V}_{\text{O}}^{\text{}\text{}}$ for M-BZO and M,N-BZO were calculated using Equation S7 and S8, respectively:

for M-BZO, $DFE[V_{O}]=\left\{ E\left( 2M+V_{O} \right)+\mu_{O} \right\}-\left\{ E\left( 2M \right)-2\mu_{e^{-}}+E_{corr} \right\}$ (Eq. S7)

for M,N-BZO, $DFE[V_{O}]=\left\{ E\left( 2M+N^{\times}+V_{O} \right)+\mu_{O} \right\}-\left\{ E\left( 2M+N^{\times} \right)-2\mu_{e^{-}}+E_{corr} \right\}$ (Eq. S8)

where $E\left( 2M \right)$ and $E\left( 2M+N^{\times} \right)$ are total energies of M-BZO and M,N-BZO, respectively; $\mu_{O}$ and $\mu_{e^{-}}$ are the chemical potential of an oxygen and an electron, respectively; and $E_{corr}$ is the energy correction term for electrostatic interactions between periodic images of the charged defects. Here, $\mu_{e^{-}}$ was set to valance band maximum of pristine BZO and $E_{corr}$ was calculated using the formula $\frac{\alpha q^{2}}{2\epsilon L}$, where α is the Madelung constant, q is the charge of the defect, L is the average distance between defects under 3D periodic boundary conditions, and ϵ is the dielectric constant of pristine BZO. The value of ϵ, calculated as the sum of ionic and electronic contributions, was found to be 60.88. The DFE of 2${OH}_{O}$ for M-BZO and M,N-BZO were calculated using Equation S9 and S10, respectively:

for M-BZO, $DFE[2{OH}_{O}]=\left\{ E\left( 2M+2{OH}_{O} \right)-2\mu_{H} \right\}-\{E\left( 2M \right)-2\mu_{e^{-}}+E_{corr}\}$ (Eq. S9)

for M,N-BZO,$DFE[2{OH}_{O}]=\left\{ E\left( 2M+N^{\times}+2{OH}_{O} \right)-2\mu_{H} \right\}-\{E\left( 2M+N^{\times} \right)-2\mu_{e^{-}}+E_{corr}\}$ (Eq. S10)

where $\mu_{H}$ is the chemical potential of a hydrogen. By substituting Equation S7 (S8) and S9 (S10) into Equation S5 (S6), $E_{hydr}$ for M-BZO (M,N-BZO) can be defined as the difference between the DFE of $V_{O}$ and $2{OH}_{O}$:

$E_{hydr}=-\{DFE\left[ V_{O} \right]-\mu_{O}\}-E\left( H_{2}O\left( g \right) \right)+\{DFE\left[ 2{OH}_{O} \right]+2\mu_{H}\}$

$E_{hydr}=-DFE\left[ V_{O} \right]+DFE\left[ 2{OH}_{O} \right]$ (Eq. S11)

where $\mu_{O}$($\mu_{H}$) is chemical potential of an oxygen (hydrogen). Here, $\mu_{O}$ was set to $\frac{1}{2}E\left( O_{2}\left( g \right) \right)$, where $E\left( O_{2}\left( g \right) \right)$ is total energy of oxygen gas, and $\mu_{H}$ was set to $\frac{1}{2}\{E\left( H_{2}O\left( g \right) \right)-\frac{1}{2}E\left( O_{2}\left( g \right) \right)\}$. The sum $\mu_{O}+2\mu_{H}$ canceled with $E\left( H_{2}O\left( g \right) \right)$. Therefore, in this study, we analyzed the dopants effects on $E_{hydr}$ by evaluating DFE of $V_{O}$ and $2{OH}_{O}$ for M-BZO and M,N-BZO.

For $E_{hydr}$, we used the most energetically favorable configurations of $2{OH}_{O}$. In M-BZO, among two different configurations of $2{OH}_{O}$ (Fig. S7a, b), configuration 2 was selected due to its lower energy (Fig. S7c and Table S6). In M,N-BZO with $M_{Zr}^{'}$=Yb and $N_{Ba}^{\times}$, two configurations (Fig. S10a,b) was considered: configuration 2 was favorable for Sr and Ra, and configuration 1 for Ca (Fig. S10c and Table S7). In M,N-BZO with $M_{Zr}^{'}$=Yb and $N_{Zr}^{\times}$, we considered three configurations (Fig. S11a-c): configuration 2 was preferred for $N_{Zr}^{\times}$=Sn, Pb, and Zr, while configuration 1 for $N_{Zr}^{\times}$=Ce, and Th (Fig. S11d and Table S8). In M,N-BZO systems with $M_{Zr}^{'}$=Yb, Tm, Er, Y, Ho and $N_{Zr}^{\times}$=Ce, Th, configuration 1 was most stable among three different arrangements (Fig. S19a,b). In M,N-BZO systems with $M_{Zr}^{'}$=Yb, Tm, Er, Y, Ho and $N_{Ba}^{\times}$=Sr, Ca, configuration 2 was stable with $N_{Ba}^{\times}$=Sr, while configuration 1 was generally preferred with $N_{Ba}^{\times}$=Ca (Fig. S19c,d and Table S9).

**D. Cubic phase stability for the proposed co-doping pairs (Yb-Th, Tm-Th, Yb-Ca, and Tm-Ca)**

Extensive experimental studies have demonstrated that both M-BZO and M,N-BZO maintain a single cubic perovskite structure without any phase transition, even when the total dopant concentration significantly exceeds the typical range of 0.1–0.2 (Table S1). Specifically, for M-BZO, cubic symmetry is maintained up to x=0.30 for M=Yb, Tm, Er, and Ho [4], and up to x=0.20 for M=In, Pr, Gd, Sc, Eu, Sm, and Dy [5,6]. For B-site isovalent ($N_{Zr}^{X}$) co-doped BZO, the cubic phase persists up to x+y=0.60 for M=Y and N=Ce [7], up to x+y=0.30 for M=Y and N=Sn [8], and up to x+y=0.45 for M=Y and N=Hf [9]. Similarly, for A-site isovalent ($N_{Ba}^{X}$) co-doped BZO, cubic symmetry is maintained up to x+y =0.60 for M=Y and N=Sr [10], and up to x+y=0.25 for M=Y and N=Ca [10]. Although direct experimental data for our proposed co-doping pairs are not yet available, the consistent retention of cubic symmetry across a wide variety of dopant species and concentrations strongly suggests that the cubic cell employed in our DFT calculations is well justified and that our proposed co-doping pairs (Yb-Th, Tm-Th, Yb-Ca, and Tm-Ca) would also maintain the cubic phase.

**E. B-site preference of acceptor dopants (M=Yb, Tm, Er, Y, Ho, Gd, Sm, Pm, and Nd)**

Previous studies demonstrate that the large rare-earth dopants considered in this work (Yb, Tm, Er, Y, Ho, Gd, Sm, Pm, and Nd; ionic radii ranging from 0.868 to 0.983 Å) predominantly occupy the B-site. Han et al.[5] investigated the site occupancy of various dopants in BaZr_0.8_M_0.2_O_3-δ_ (M=Yb, Tm, Er, Y, Ho, Gd, and Sm) using Rietveld refinement of X-ray diffraction patterns. They confirmed that the rare-earth dopants occupy the B-site, and even for significantly large lanthanide dopants such as Gd and Sm, B-site occupancy is dominant, while A-site occupation is negligible. In addition, Shuhaib et al.[13] observed lattice expansion in Nd-doped BZO through Rietveld refinement, indicating that Nd (0.983 Å) substitutes at the smaller Zr-site (0.72 Å) rather than the larger Ba-site (1.42 Å).Consistent with these observations, Sundell et al.[14] evaluated defect formation energies through density functional theory (DFT) calculations and showed that Gd preferentially occupies the B-site under most chemical-potential conditions.

To further verify the site preference of rare-earth dopants in BZO, we calculated the formation energies of A-site doped ($E_{form}^{A-site}$) and B-site doped BZO ($E_{form}^{B-site}$) for all rare-earth dopants (M=Yb, Tm, Y, Ho, Gd, Sm, Pm, and Nd) considered in this work. The $E_{form}^{B-site, M}$ and $E_{form}^{A-site, M}$ were calculated using the following equation (1) and (2):

$E_{form}^{B-site}=E\left( Ba{Zr}_{1-x}M_{x}O_{3} \right)-E\left( BaO \right)-\frac{x}{2}E\left( M_{2}O_{3} \right)-\left( 1-x \right)E\left( ZrO_{2} \right)-\frac{x}{4}E\left( O_{2} \right)$ (1)

$E_{form}^{A-site}=E\left( {Ba}_{1-x}M_{x}ZrO_{3} \right)-\left( 1-x \right)E\left( BaO \right)-\frac{x}{2}E\left( M_{2}O_{3} \right)-E\left( ZrO_{2} \right)+\frac{x}{4}E\left( O_{2} \right)$ (2)

where $E\left( {Ba}_{1-x}M_{x}ZrO_{3} \right)$ and $E\left( Ba{Zr}_{1-x}M_{x}O_{3} \right)$ are total energies of the A-site doped and B-site doped BZO, respectively, $E\left( BaO \right)$, $E\left( M_{2}O_{3} \right)$, $E\left( ZrO_{2} \right)$, and $E\left( O_{2} \right)$ are total energies of crystalline BaO($\mathrm{Fm}\bar{3}m$), $M_{2}O_{3}(IA\bar{3})$, $ZrO_{2}\left( P2_{1}/c \right),$ and $O_{2}$ molecule, respectively, and x=0.03125. As shown in Table S2, $E_{form}^{B-site, M}$ are consistently lower than $E_{form}^{A-site, M}$ across all dopants, which indicate that B-site occupancy is thermodynamically favorable regardless of the dopant ionic radius. Thus, based on these previous studies and our computational results, we have confirmed that the large rare-earth acceptors (Yb, Tm, Er, Y, Ho, Gd, Sm, Pm, and Nd) preferentially substitute on the B-site in BZO.

**F. Energy differences among various configurations of oxide-ion vacancies (**$\boldsymbol{V}_{\boldsymbol{O}}$**) and protons (**$\boldsymbol{OH}_{\boldsymbol{O}}$**) and their effects on hydration behavior**

We calculated the binding energies between oxide-ion vacancies ($V_{O}$) and dopants to predict the energetically preferred $V_{O}$ position in acceptor and isovalent co-doped BaZrO_3_ (M,N-BZO). $V_{O}$ was placed adjacent to the B-site acceptor ($M_{Zr}^{'}$= Nd, Pm, Sm, Gd, Y, Ho, Er, Tm, and Yb; Fig. S2a), the B-site isovalent dopant ($N_{Zr}^{\times}$= Sn, Hf, Pb, Ce, and Th; Fig. S2b), and the A-site isovalent dopant ($N_{Ba}^{\times}$= Ca, Sr, and Ra; Fig. S2c). The calculated binding energies show that $V_{O}$ is more strongly stabilized near $M_{Zr}^{'}$ (-0.60 to -0.25 eV for Nd to Yb) compared to $N_{Zr}^{\times}$ (-0.20 to 0.14 eV for Ce to Hf) or $N_{Ba}^{\times}$ (-0.13 to -0.03 eV for Ca to Ra). Based on these results, $V_{O}$ is predicted to preferentially adopt the M-V_O_-M configuration, as this configuration maximizes favorable binding interactions with acceptors by forming two M–V_O_ bindings.

To verify this prediction, we calculated the energy differences among various $V_{O}$ configurations in M,N-BZO. For $N_{Ba}^{\times}$ co-doped BZO ($M_{Zr}^{'}$= Yb, $N_{Ba}^{\times}$= Ca), four representative configurations were considered: configuration 1 with two M-V_O_ bindings forming the M-V_O_-M (Fig. S4a), configuration 2 with one M-V_O_ binding (Fig. S4b), configuration 3 with one $N_{Ba}^{\times}$-V_O_ binding (Fig. S4c), and configuration 4 with no dopant-V_O_ binding (Fig. S4d). Energy comparisons relative to configuration 1 show that configuration 2 is 0.27 eV higher, configuration 3 is 0.38 eV higher, and configuration 4 is 0.54 eV higher (Fig. S4e). This energetic ordering follows the trend predicted by the binding energy calculations: configurations with two M-V_O_ bindings are most stable, followed by those with one M-V_O_ binding, then one $N_{Ba}^{\times}$-V_O_ binding, and finally configurations with no dopant-V_O_ binding. A similar trend is also observed for $N_{Zr}^{\times}$ co-doped BZO ($M_{Zr}^{'}$= Yb, $N_{Zr}^{\times}$= Th). For five representative configurations (Fig. S5a-e), configuration 1 with two M-V_O_ bindings is most stable (ΔE= 0 eV), followed by configuration 2 and 3 with one M-V_O_ binding (ΔE= 0.29 and 0.39 eV, respectively), then configuration 4 with one $N_{Zr}^{\times}$-V_O_ binding (ΔE= 0.69 eV), and finally configuration 5 with no dopant-V_O_ binding (ΔE= 0.83 eV) (Fig. S5f). Since the same trend holds across both systems, we confirmed that $V_{O}$ preferentially adopts the M-V_O_-M configuration to maximize favorable M-V_O_ binding interactions. This M-V_O_-M preference is further supported by atom probe tomography (APT) observation showing a prevalence of M–M pairs [15]

Since hydration proceeds through incorporation of H_2_O into pre-existing $V_{O}$, the energetically favorable M-V_O_-M configuration becomes the primary site for hydration behavior in M,N-BZO. While other $V_{O}$ configurations - such as $V_{O}$ adjacent to isovalent dopants or $V_{O}$ not bound to any dopant - may also exist, their weaker binding energies lead to a lower population of $V_{O}$ at these sites, and thus a smaller contribution to the overall hydration. Therefore, our study focused on M-V_O_-M configuration to analyze the hydration energetics in the co-doped systems.

Additionally, we analyzed different configurations of protons (${OH}_{O}$) in M,N-BZO to examine how co-doping affects proton stability after hydration. . The $N_{Ba}^{\times}$ co-doped system has three types of proton binding sites: M–O–M, M–O–Zr, and Zr–O–Zr (Fig. S6a-c), whereas the $N_{Zr}^{\times}$ co-doped system exhibits four distinct proton binding sites: M-O-M, M-O-Zr, M-O-N , and Zr-O-N (Fig. S7a-d). In all cases, M-O-M is the most stable proton binding site, while the second most stable binding site depends on the ionic radius of $N_{Ba}^{\times}$ ($N_{Zr}^{\times}$). In the $N_{Ba}^{\times}$ co-doped BZO, the preferred secondary binding site shifts from Zr–O–Zr to M–O–Zr as the ionic radius decreases from Ra to Ca (Fig. S6d). Similarly, in the $N_{Zr}^{\times}$ co-doped BZO, the secondary binding preference changes from Zr–O–N to M–O–Zr as the dopant ionic radius increases from Sn to Th (Fig. S7e). This indicates that co-doping with $N_{Ba}^{\times}$ ($N_{Zr}^{\times}$) further stabilizes the 2${OH}_{O}$ configuration by providing a more stable secondary binding site for ${OH}_{O}$, thereby enhancing hydration.

In summary, our analysis of various defect configurations reveals that $V_{O}$ is the most stable near $M_{Zr}^{'}$, making the M-M pair the primary site for hydration. In addition, the second stable binding site of ${OH}_{O}$ varies with the ionic radius of $N_{Ba}^{\times}$ ($N_{Zr}^{\times}$), which further enhances hydration by providing a more stable 2${OH}_{O}$ configuration.

**G. Defect formation energy (DFE) differences among various configurations of 2**$\boldsymbol{OH}_{\boldsymbol{O}}$ **in M,N-BZO and their effects on proton movement**

The M-V_O_-M configuration is energetically favorable due to the strong M-V_O_ binding and serves as the primary site for hydration. Upon hydration, an H_2_O molecule dissociates into OH and H at the $\text{V}_{\text{O}}^{\text{}\text{}}$ of M-V_O_-M, forming two ${OH}_{O}$. Specifically, the OH fills the $\text{V}_{\text{O}}^{\text{}\text{}}$ to form the first ${OH}_{O}$ at M-O-M, while the H bonds with a neighboring lattice oxygen to form the second ${OH}_{O}$. Since the first ${OH}_{O}$ at M-O-M is strongly bound, proton movement after hydration is expected to occur preferentially from the second ${OH}_{O}$. Therefore, to analyze proton movement, we calculated the DFE for two configurations that differ in the position of the second ${OH}_{O}$ : (ⅰ) the as-hydrated configuration, where the second ${OH}_{O}$ remains near the hydration site, and (ⅱ) the migrated configuration, where the second ${OH}_{O}$ has moved farther away while the first ${OH}_{O}$ remains at M-O-M.

In the $N_{Ba}^{\times}$ co-doped system, the second ${OH}_{O}$ can occupy two sites: M-O-Zr or Zr-O-Zr (Fig. S10a, b). M-O-Zr is adjacent to M-O-M, and corresponds to the as-hydrated configuration, whereas Zr-O-Zr is located farther from M-O-M and corresponds to the migrated configuration. Our calculations show that as the ionic radius of $N_{Ba}^{\times}$ increases from Ca to Ra, the DFE of the migrated configuration becomes lower than that of the as-hydrated configuration (Fig. S10c). This indicates that depending on the choice of $N_{Ba}^{\times}$, protons may preferentially stabilize at sites farther from the initial hydration position rather than remaining at the as-hydrated site. Such variation in the preferred proton position suggests that proton mobility could also be influenced by co-doping.

In contrast, in the $N_{Zr}^{\times}$ co-doped system, the second ${OH}_{O}$ can occupy three sites: M-O-Zr, M-O-N, or Zr-O-N (Fig. S11a-c). M-O-Zr and M-O-N are adjacent to M-O-M and correspond to the as-hydrated configurations 1 and 2, respectively, whereas Zr-O-N is located farther from M-O-M and corresponds to the migrated configuration. Our calculations show that as the ionic radius of $N_{Zr}^{\times}$ decreases from Th to Sn, the DFE of the migrated configuration becomes lower than that of the as-hydrated configurations, indicating that the preferred second ${OH}_{O}$ site shifts from M–O–Zr to Zr–O–N (Fig. S11d). This suggests that the stable proton position after hydration is also sensitive to the choice of $N_{Zr}^{\times}$, further supporting that proton mobility could be influenced by co-doping.

In summary, the type of isovalent co-dopant alters the DFE of different 2${OH}_{O}$• configurations by changing the energetically preferred proton position after hydration. Specifically, larger ionic radii in the $N_{Ba}^{\times}$ co-doped system and smaller ionic radii in the $N_{Zr}^{\times}$ co-doped system favor proton formation at sites away from the initial hydration position. These results suggest that proton mobility could be influenced by co-doping through modification of the preferred proton site. However, quantitative prediction of proton diffusion kinetics would require migration energy barrier calculations

**H. Comparison of PBE and HSE06-calculated hydration energies (**$\mathbf{E}_{\boldsymbol{hydr,calc}}$**) with experimental hydration energies (**$\mathbf{E}_{\boldsymbol{hydr,expt}}$**) and evaluation of the predicted superiority of the four proposed co-doping pairs using the HSE06 functional.**

We performed additional HSE06 calculations to re-examine the correlation between calculated hydration energies ($E_{hydr, calc}$) and $E_{hydr, expt}$. HSE06 hybrid functionals provide a more accurate description of defect energetics at a higher computational cost [16,17]. Our results show that the HSE06-calculated $E_{hydr, calc}$ also exhibits a strong correlation with the experimental data (R² = 0.93), comparable to the PBE calculations reported in the manuscript (R² = 0.88) (Fig. S12). The consistency across two different functionals supports the robustness of the experiment–calculation correlation despite the limited experimental sampling.

Furthermore, we re-evaluated the predicted superiority of our four proposed co-doping pairs (Yb-Th, Tm-Th, Yb-Ca, and Tm-Ca) using the HSE06 functional. Consistent with the PBE calculations in our manuscript, the HSE06 results show that these four pairs exhibit more exothermic hydration energies than the conventional Y-Ce pair, with $\Delta E_{hydr, calc}$ of -0.19 eV for Yb-Th, -0.17 eV for Tm-Th, -0.25 eV for Yb-Ca, and -0.14 eV for Tm-Ca relative to Y-Ce. Therefore, we believe that the strong experiment-calculation correlation comparison validates our computational approach and that our proposed co-doping pairs can exhibit excellent hydration behavior.

We conducted an extensive literature survey, including studies published up to 2026. However, experimental reports providing quantitative $E_{hydr, expt}$ (e.g., via van't Hoff-type analysis) for acceptor-doped or acceptor and isovalent co-doped BaZrO_3_ remain limited. Although many studies report proton conductivity or qualitative hydration trends, only a few extract $E_{hydr, expt}$ suitable for direct comparison with our DFT calculations. Consequently, we were unable to identify additional experimental datasets beyond the five co-doping pairs already included in our study.

**I. Structural descriptors governing the H–A distance upon isovalent co-doping**

Local lattice strain due to smaller ionic radius of A-site isovalent dopant ($N_{Ba}^{X}$) than Ba may affect proton binding. To identify how such strain affects proton binding, we analyzed the correlation between defect formation energy (DFE) of $2{OH}_{O}$ and three structural parameters in the vicinity of protons: the mean hydrogen-bond length of two ${OH}_{O}$ (mean H-bond length), and the mean distance from two ${OH}_{O}$ to their respective two nearest A- or B-site cations (mean H-A distance or mean H-B distance). The results show that only the mean H-A distance exhibits a strong correlation with DFE of $2{OH}_{O}$ (R² = 0.94, Fig. S13a), while the mean H-B distance (R² = 0.31, Fig. S13b) and mean H-bond length (R² = 0.30, Fig. S13c) show negligible correlations. An increased H-A distance reduces electrostatic repulsion between protons and A-site cations, thereby stabilizing proton defects. This suggests that the various local lattice strain effects induced by $N_{Ba}^{X}$ substitution are collectively manifested through changes in the H-A distance, making it the dominant descriptor governing proton stability.

**J. Validation of synergistic effects of co-doping under clustered dopant configurations**

We performed DFT calculations to examine whether the synergistic effects are maintained under enhanced dopant-dopant interactions at higher concentrations.

We investigated four acceptor-doped BZO (BaZr_1-x_M_x_O_3_; $M_{Zr}^{'}$=Nd, Y, Tm, Yb; x=0.125) and three isovalent co-doped BZO: two B-site isovalent co-doped BZO (BaZr_1-x-y_M_x_N_y_O_3_; $M_{Zr}^{'}$=Yb; $N_{Zr}^{\times}$= Ce, Th; x=0.125; y=0.0625) and one A-site isovalent co-doped BZO (Ba_1-y_N_y_Zr_1-x_M_x_O_3_; $M_{Zr}^{'}$=Yb; $N_{Ba}^{\times}$=Ca; x=0.125; y=0.0625). To increase dopant–dopant interactions, we doubled the dopant concentrations in the supercell with 32 B-sites (32 A-sites): the acceptor concentration was increased from x = 0.0625 (2/32 B-sites) to x = 0.1250 (4/32), and the isovalent dopant concentration from y = 0.03125 (1/32) to y = 0.06250 (2/32). At these elevated concentrations, each supercell contains two M-V_O_-M defect units. We first evaluated the clustering tendency of these defect units by calculating the binding energy between them. A negative binding energy indicates that clustering is thermodynamically favorable, whereas a positive value indicates a preference for spatial separation. The calculated binding energies are negative for all systems investigated, confirming that the two M-V_O_-M units preferentially cluster together at increased concentrations. For M-BZO, the binding energies are -0.59 eV, -0.37 eV, -0.30 eV, and -0.28 eV for M = Nd, Y, Tm, and Yb, respectively. For M,N-BZO, the binding energies are -0.46 eV, -0.54 eV, and -0.26 eV for Yb-Ce, Yb-Th, and Yb-Ca, respectively. Based on these results, we adopted the clustered configurations (Fig. S15a for M-BZO; Fig. S16a for $N_{Zr}^{\times}$ co-doped BZO; Fig. S16b for $N_{Ba}^{\times}$ co-doped BZO) to evaluate the synergistic co-doping effects under high-concentration conditions.

For M-BZO, we investigated whether the effects of acceptor doping on hydration are maintained at the increased concentration. Using the clustered configurations, we calculated defect formation energies (DFE) of $V_{O}$ (Fig. S15a) and hydration energy ($E_{hydr}$) (Fig. S15b). As the acceptor changes from Nd to Yb, the DFE of $V_{O}$ increases from 0.18 eV (Nd) to 0.70 eV (Tm) and 0.76 eV (Yb), while $E_{hydr}$ decreases from −0.50 eV (Nd) to -0.99 eV (Tm) and -1.01 eV (Yb) (R² = 0.97, Fig. S15c). This linear correlation between DFE of $V_{O}$ and $E_{hydr}$ is consistent with that observed at x=0.0625. Although the absolute values differ slightly, the underlying trend remains unchanged: acceptor doping suppresses dehydration by increasing DFE of $V_{O}$, which reduces the probability of oxygen desorption, and Yb and Tm remain the most effective acceptors.

Next, M,N-BZO systems are investigated. We calculated DFE of 2${OH}_{O}$ (Fig. S16a,b) and hydration energy ($E_{hydr}$) (Fig. S16c,d) using the clustered configurations. As the DFE of 2${OH}_{O}$ decreases from -0.25 eV (Zr) to -0.38 eV (Ce) and -0.54 eV (Th) for $N_{Zr}^{\times}$ and to -0.51 eV (Ca) for $N_{Ba}^{\times}$, $E_{hydr}$ correspondingly decreases from -0.01 eV (Zr) to -1.14 eV (Ce) and -1.23 eV (Th) for $N_{Zr}^{\times}$, and to −1.27 eV (Ca) for $N_{Ba}^{\times}$ (R^2^=0.94, Fig. S16d). This linear correlation between DFE of 2${OH}_{O}$ and $E_{hydr}$ is consistent with that observed at x=0.0625: isovalent co-doping enhances hydration by stabilizing 2${OH}_{O}$, with Th and Ca remaining the most effective isovalent dopants.

In summary, these results confirm that the increased dopant interactions at higher dopant concentration are not crucial to the effect of acceptor doping on formation of $V_{O}$ or the effect of isovalent co-doping on formation of 2${OH}_{O}$. Therefore, the synergistic effects of acceptor–isovalent co-doping on hydration behavior remains robust and persist even under practically relevant concentration.

**K. Validation of synergistic effects of co-doping at finite temperatures**

We further investigated how finite-temperature effects influence hydration behavior in the operating temperature range of 500–700 °C (773-973 K) for both acceptor-doped BaZrO_3_ (M-BZO), and acceptor and isovalent co-doped BaZrO_3_ (M,N-BZO). As described in our ESI, the hydration energy ($E_{hydr}$) can be decomposed in terms of the defect formation energies (DFE) of one oxygen vacancy ($\text{V}_{\text{O}}^{\text{}\text{}}$) and two protons (2${OH}_{O}$):

$E_{hydr}=-DFE[V_{O}]+DFE[2{OH}_{O}]$ (Eq. 1)

This decomposition allows us to systematically evaluate how dopants modulate hydration by altering the relative stabilities of $\text{V}_{\text{O}}^{\text{}\text{}}$ and 2${OH}_{O}$. To incorporate temperature dependence, we reformulated Eq. 1 in terms of Gibbs free energies:

$\Delta G_{hydr}\left( T, P \right)=\Delta G_{form, 2{OH}_{O}}\left( T, P \right)-\Delta G_{form, V_{O}}\left( T, P \right)$ (Eq. 2)

where $\Delta G_{hydr}$, $\Delta G_{form, V_{O}}$, and $\Delta G_{form, 2{OH}_{O}}$ represent the Gibbs free energies of the hydration reaction, the $\text{V}_{\text{O}}^{\text{}\text{}}$ formation reaction, and the 2${OH}_{O}$ formation reaction, respectively. $\Delta G_{form, V_{O}}$ and $\Delta G_{form, 2{OH}_{O}}$ are defined as:

$\Delta G_{form, V_{O}}\left( T, P \right)=G_{defective, V_{O}}\left( T, P \right)-G_{perfect}\left( T, P \right)+\frac{1}{2}G_{O_{2}}\left( T, P \right)$ (Eq. 3)

$\Delta G_{form, 2{OH}_{O}}\left( T, P \right)=G_{defective, 2{OH}_{O}}\left( T, P \right)-G_{perfect}\left( T, P \right)-(G_{H_{2}O}\left( T, P \right)-\frac{1}{2}G_{O_{2}}\left( T, P \right))$ (Eq. 4)

where $G_{perfect}$, $G_{defective, V_{O}}$, and $G_{defective, 2{OH}_{O}}$, represent the Gibbs free energies of the perfect structure, oxygen deficient structure, and hydrated structure for M-BZO or M,N-BZO, respectively, while $G_{O_{2}}$ and $G_{H_{2}O}$ represent the Gibbs free energies of O_2_ and H_2_O gas, respectively. In Eq. 3–4, we compare solid-phase Gibbs free energies within the same host lattice, thus contributions from thermal expansion and vibrational entropy largely cancel in the energy differences. Therefore, the solid-phase Gibbs free energy can be approximated by the DFT-calculated energy (E):

$G_{solid}\left( T,P \right)= E_{DFT}+PV-TS\approx E_{DFT}$ (Eq. 5)

Applying this approximation to Eqs. 3–4 yields:

$\Delta G_{form, V_{O}}\left( T, P \right)=E_{defective, V_{O}}-E_{perfect}+\frac{1}{2}G_{O_{2}}\left( T, P \right)$ (Eq. 6)

$\Delta G_{form, 2{OH}_{O}}\left( T, P \right)=E_{defective, 2{OH}_{O}}-E_{perfect}-(G_{H_{2}O}\left( T, P \right)-\frac{1}{2}G_{O_{2}}\left( T, P \right))$ (Eq. 7)

In contrast to the solid phase, the temperature and pressure dependence of gas-phase Gibbs free energies must be explicitly included. We calculated $G_{O_{2}}$ and $G_{H_{2}O}$ using standard thermochemical data from the NIST Chemistry WebBook:

$G_{O_{2}\left( g \right)}\left( T, P \right)=E_{O_{2}\left( g \right)}+(H_{O_{2}\left( g \right)}\left( T, P_{0} \right)-H_{O_{2}\left( g \right)}\left( 0, P_{0} \right))-TS_{O_{2}\left( g \right)}\left( T, P_{0} \right)+k_{B}T\ln\left( \frac{P_{O_{2}}}{P_{0}} \right)$ (Eq. 8)

$G_{H_{2}O\left( g \right)}\left( T, P \right)=E_{H_{2}O\left( g \right)}+(H_{H_{2}O\left( g \right)}\left( T, P_{0} \right)-H_{H_{2}O\left( g \right)}\left( 0, P_{0} \right))-TS_{H_{2}O\left( g \right)}\left( T, P_{0} \right)+k_{B}Tln(\frac{P_{H_{2}O}}{P_{0}})$ (Eq. 9)

where H(T,P_0_) and S(T,P_0_) are the standard enthalpy and entropy at temperature (T) and reference pressure (P_0_=1 atm), respectively, and $P_{O_{2}}$ and $P_{H_{2}O}$ are the partial pressures of O_2_ and H_2_O gas, respectively. Using this framework, we investigated $\Delta G_{hydr}$ for M-BZO and M,N-BZO under the typical operating conditions: temperatures ranging from 800 K to 1000 K under wet O_2_ atmosphere ($P_{O_{2}}$=0.203 atm, $P_{H_{2}O\left( g \right)}$=0.03 atm).

We first examined how finite-temperature effects changes $\Delta G_{form, V_{O}}$ and $\Delta G_{form, 2{OH}_{O}}$ for both M-BZO and M,N-BZO. As temperature increases from 0 K to 800 K and 1000 K, the entropic contribution to Gibbs free energy favors gaseous O_2_ release, stabilizing $\text{V}_{\text{O}}^{\text{}\text{}}$ formation and decreasing $\Delta G_{form, V_{O}}$ by −0.91 eV at 800 K and −1.17 eV at 1000 K relative to 0 K. In contrast, the entropic contribution disfavor H_2_O incorporation into the lattice, destabilizing 2${OH}_{O}$ formation and increasing $\Delta G_{form, 2{OH}_{O}}$ by +0.90 eV at 800 K and +1.17 eV at 1000 K relative to 0 K. These opposing shifts in $\Delta G_{form, V_{O}}$ and $\Delta G_{form, 2{OH}_{O}}$ - $\text{V}_{\text{O}}^{\text{}\text{}}$ stabilization and 2${OH}_{O}$ destabilization - act in concert to destabilize hydration. As a result, $\Delta G_{hydr}$ increases by +1.81 eV at 800 K and +2.34 eV at 1000 K relative to 0 K for both M-BZO and M,N-BZO, indicating that dehydration becomes thermodynamically more favorable under operating conditions. This temperature-induced upshift in $\Delta G_{hydr}$is consistent with the well-known experimental observation that proton conducting oxide (PCO) lose water content at elevated temperatures.

Next, we investigated whether the synergistic effects of co-doping on hydration remain robust under the operating condition.

For M-BZO ($M_{Zr}^{'}$= Nd, Pm, Sm, Gd, Y, Ho, Er, Tm, Yb), we compared $\Delta G_{form, V_{O}}$ and $\Delta G_{hydr}$ at 0K, 300K, 800K, and 1000K (Fig. S17a). While $\Delta G_{form, V_{O}}$ shift leftward and $\Delta G_{hydr}$ shift upward with increasing temperature, the negative correlation between$\Delta G_{form, V_{O}}$ and $\Delta G_{hydr}$ is preserved at all temperatures: systems with more unstable $\text{V}_{\text{O}}^{\text{}\text{}}$ consistently exhibit more favorable hydration. This indicates that acceptor doping still enhance hydration by modulating $\text{V}_{\text{O}}^{\text{}\text{}}$ stability under the operating conditions. For M,N-BZO ($M_{Zr}^{'}$= Yb; $N_{Zr}^{X}$ = Sn, Hf, Pb, Ce, Th or $N_{Ba}^{X}$ = Ca, Sr, Ra), we compared$\Delta G_{form, 2{OH}_{O}}$ and $\Delta G_{hydr}$ at 0K, 300K, 800K, and 1000K (Fig. S17b). While $\Delta G_{form, 2{OH}_{O}}$ shift rightward and $\Delta G_{hydr}$ shift upward with increasing temperature, the positive correlation between$\Delta G_{form, 2{OH}_{O}}$ and $\Delta G_{hydr}$ is maintained at all temperatures: systems with more stable 2$\text{OH}_{\text{O}}^{\text{}}$ consistently exhibit more favorable hydration. Isovalent co-doping still enhance hydration by stabilizing 2$\text{OH}_{\text{O}}^{\text{}}$ under operating conditions. These finite-temperature analyses demonstrate that the synergistic effects of co-doping are preserved across the entire operating temperature range. Therefore, we believe that the co-doping design rules derived from DFT calculations remain valid under realistic operating condition.

We note that in the manuscript, DFT energies are used rather than temperature-dependent free energies to enable direct comparison with experimental hydration enthalpies reported in the literature. Since the enthalpies exclude entropic contributions, DFT energies provide a more appropriate benchmark for validation (Fig. 1c).

**L. Validation of synergistic effects of co-doping using the HSE06 functional**

We have performed additional HSE06 benchmark calculations for five acceptor-doped systems ( BaZr_1-x_M_x_O_3_; $M_{Zr}^{'}$=Yb, Er, Y, Gd, Nd; x=0.0625 ) and three acceptor and isovalent co-doped systems (BaZr_1-x-y_M_x_N_y_O_3_ ($M_{Zr}^{'}$=Yb;$N_{Zr}^{\times}$= Ce, Th; x=0.0625; y=0.03125); BaZr_1-x-y_M_x_N_y_O_3_ ($M_{Zr}^{'}$=Yb; $N_{Ba}^{\times}$=Ca; x=0.0625; y=0.03125)) to verify our PBE calculation results. As shown in Table S3, the mean absolute deviation (MAD) between PBE and HSE06 is 0.28 eV for oxygen vacancy defect formation energy (DFE[$V_{O}$]) and 0.36 eV for proton defect formation energy (DFE[$2{OH}_{O}$]). HSE06 tends to yield slightly lower values for both DFEs compared to PBE. Although the offset exists between PBE and HSE06 calculations, the physical trends identified in our PBE calculations are well reproduced at the HSE06 level. For the five acceptor-doped systems, increased DFE[$V_{O}$] leads to more favorable hydration energy ($E_{hydr}$) (Fig. S18a, R² = 0.90). Similarly, for the three co-doped systems, proton stabilization contributes to lowering $E_{hydr}$ (Fig. S18b, R² = 0.90). These results demonstrate that the key trends and design principles proposed in this work are robust with respect to the functional choice.

**M. Quantitative decomposition of synergistic effects for the proposed co-doping pairs (Yb-Th, Tm-Th, Yb-Ca, and Tm-Ca)**

We quantified each contribution of acceptor and isovalent co-doping to hydration energy ($E_{hydr}$) for our proposed co-doping pairs (Yb-Th, Tm-Th, Yb-Ca, and Tm-Ca). The contribution of acceptor Yb (Tm) is defined as the hydration energy difference between conventional Y-doped BaZrO_3_ (Y-BZO) and Yb(Tm)-BZO. The contribution of isovalent dopant Th(Ca) is defined as the hydration energy difference between M-BZO and M,Th(Ca)-BZO (M=Yb,Tm). The results are summarized in Table S4. Replacing Y with Yb and Tm improves $E_{hydr}$ by -0.11 eV and -0.10 eV, respectively. Co-doping with Th provides an additional improvement of -0.20 eV for both Yb-BZO and Tm-BZO, while co-doping with Ca provides an additional improvement of -0.29 eV and -0.27 eV for Yb-BZO and Tm-BZO, respectively. The relative contributions of acceptor and isovalent dopants to the total improvement are 35% and 65% for Yb-Th, 33% and 67% for Tm-Th, 28% and 72% for Yb-Ca, and 27% and 73% for Tm-Ca. These results demonstrate that isovalent dopants contribute approximately twice as much as acceptor optimization to the enhancement in $E_{hydr}$.

**N. Chemical stability against CO_2_ and H_2_O for the proposed co-doping pairs (Yb-Th, Yb-Ca, Tm-Th, Tm-Ca)**

We performed additional calculations to investigate how our proposed co-doping pairs (Yb-Th, Yb-Ca, Tm-Th, Tm-Ca) affect the chemical stability of BaZrO_3_(BZO)-based proton conducting oxides (PCO). Under CO_2_ and H_2_O-containing atmospheres, BZO-based and BaCeO_3_(BCO)-based PCO can decompose via carbonation and hydroxylation reaction [1,2]. For undoped systems, these reactions proceed as:

Carbonation: BaZr(Ce)O_3_ (s) + CO_2_ (g) → BaCO_3_ (s) + Zr(Ce)O_2_ (s) (Reaction 1)

Hydroxylation: BaZr(Ce)O_3_ (s) + H_2_O (g) → Ba(OH)_2_ (s) + Zr(Ce)O_2_ (s) (Reaction 2)

For co-doped BZO systems, dopants form additional decomposition products: M_2_O_3_ ($\mathrm{Ia}\bar{3}$) for acceptors (M=Yb, Th), NO_2_ ($\mathrm{Fm}\bar{3}m$) for B-site isovalent dopant ($N_{Zr}^{X}$=Th), and for A-site isovalent dopant ($N_{Ba}^{X}$=Ca), NCO_3_ ($R\bar{3}c$) in carbonation or N(OH)_2_ ($P\bar{3}m1$) in hydroxylation. Accordingly, Reaction 1 and Reaction 2 are modified as follows:

Carbonation for $N_{Zr}^{X}$ co-doped BZO:

BaZr_1-x-y_M_x_N_y_O_3_ (s) + CO_2_ (g) → BaCO_3_ (s) + (1-x-y)ZrO_2_ (s) + (x/2)M_2_O_3_ (s) + yNO_2_ (s) + (x/4)O_2_ (g) (Reaction 3)

Carbonation for $N_{Ba}^{X}$ co-doped BZO:

Ba_1-y_N_y_Zr_1-x_M_x_O_3_ (s) + CO_2_ (g) → (1-y)BaCO_3_ (s) + yNCO_3_ (s) + (1-x)ZrO_2_ (s) + (x/2)M_2_O_3_ (s) + (x/4)O_2_ (g) (Reaction 4)

Hydroxylation for $N_{Zr}^{X}$ co-doped BZO:

BaZr_1-x-y_M_x_N_y_O_3_ (s) + H_2_O (g) → Ba(OH)_2_ (s) + (1-x-y)ZrO_2_ (s) + (x/2)M_2_O_3_ (s) + yNO_2_ (s) + (x/4)O_2_ (g) (Reaction 5)

Hydroxylation for $N_{Ba}^{X}$ co-doped BZO:

Ba_1-y_N_y_Zr_1-x_M_x_O_3_ (s) + H_2_O (g) → (1-y)Ba(OH)_2_ (s) + yN(OH)₂ (s) + (1-x)ZrO_2_ (s) + (x/2)M_2_O_3_ (s) +(x/4)O_2_ (g) (Reaction 6)

Based on these reactions, we calculated the reaction energies of carbonation ($\Delta E_{carbonation}$) and hydroxylation ($\Delta E_{hydroxylation}$) for BCO, BZO, and the co-doped BZO to evaluate the effects of our proposed co-doping pairs on the chemical stability. The calculated reaction energies are summarized in Table S5. BCO exhibits poor chemical stability with $\Delta E_{carbonation}$= -2.24 eV and $\Delta E_{hydroxylation}$= -1.21 eV. In contrast, undoped BZO shows significantly improved stability with $\Delta E_{carbonation}$= -1.57 eV and $\Delta E_{hydroxylation}$= -0.55 eV, which are less negative than those of BCO by 0.67 eV and 0.66 eV, respectively. This substantial enhancement is consistent with the experimentally established superior chemical stability of BZO-based PCOs over BCO-based PCOs. For our proposed co-doping pairs, $\Delta E_{carbonation}$ is -1.70 eV for Yb-Th, -1.66 eV for Yb-Ca, -1.71 eV for Tm-Th, and -1.67 eV for Tm-Ca, and $\Delta E_{hydroxylation}$ is -0.68 eV for Yb-Th, -0.65 eV for Yb-Ca, -0.68 eV for Tm-Th, and -0.66 eV for Tm-Ca. Compared to BCO, these co-doped systems exhibit substantial improvements of 0.53–0.58 eV for carbonation and 0.53–0.56 eV for hydroxylation, indicating excellent chemical stability. While co-doping introduces a slight reduction in stability relative to undoped BZO, the proposed co-doped systems remain far more stable than BCO-based PCOs. We confimed that the inherent chemical stability advantage of BZO-based PCOs is well preserved in our proposed co-doped systems.

**O. Migration barrier energies for the proposed co-doping pairs (Tm-Th, Yb-Th, Tm-Ca, and Yb-Ca)**

We performed additional calculations to compare the proton diffusion barrier of our proposed co-doping pairs (Tm-Th, Yb-Th, Tm-Ca, and Yb-Ca) with the conventional Y-Ce co-doping pair.

In Y-Ce co-doped systems, inter-octahedral proton diffusion is activated, where protons migrate between oxygen sites on different octahedra [18-21]. For direct comparison, we investigated the inter-octahedral diffusion pathway for both Y-Ce and our proposed co-doping pairs. For the B-site isovalent ($N_{Zr}^{X}$) co-doping pairs (Y-Ce, Tm-Th, and Yb-Th), we calculated the migration barrier energy ($E_{migr}$) for proton diffusion from the M-O-Zr in one octahedron (initial state) to the M-O-N in the neighboring octahedron (final state) (Fig. S20a). Since $N_{Zr}^{X}$ occupies the B-site, it directly changes the local environment of final state from M-O-Zr to M–O–N, resulting in distinct initial (M–O–Zr) and final state (M–O–N). Accordingly, these $N_{Zr}^{X}$ co-doping pairs exhibit asymmetric paths with energy differences between initial and final states of 0.05 eV for Y-Ce and 0.07 eV for Yb-Th and Tm-Th (Fig. S20b). For the A-site isovalent ($N_{Ba}^{X}$) co-doping pairs (Yb-Ca and Tm-Ca), we calculated $E_{migr}$ for proton diffusion from the M-O-Zr in one octahedron (initial state) to the M-O-Zr in the neighboring octahedron (final state) (Fig. S21a). Since $N_{Ba}^{X}$ resides on the A-site, it does not alter the B–O–B framework, resulting in identical local environments at the initial and final states. Accordingly, these$N_{Ba}^{X}$ co-doping pairs exhibit symmetric migration paths from M-O-Zr to M-O-Zr with identical initial and final state energies (Fig. S21b).

Next, we compared $E_{migr}$ for our proposed co-doping pairs with the Y-Ce pair. Our results show that $E_{migr}$ for Y-Ce pair is 0.15 eV, while $E_{migr}$ for the proposed pairs are 0.16 eV for Tm-Th, 0.17 eV for Yb-Th (Fig. S20b), 0.24 eV for Tm-Ca, and 0.26 eV for Yb-Ca (Fig. S21b). Our proposed pairs show modest increases of $E_{migr}$: $\Delta E_{migr}$= 0.01 eV for Tm-Th, 0.02 eV for Yb-Th, 0.09 eV for Tm-Ca, and 0.11 eV for Yb-Ca relative to Y-Ce. However, this modest penalty in migration kinetics is compensated by significant improvements in hydration thermodynamics. Hydration energy ($E_{hydr}$) for Y-Ce pair is -1.16 eV, while $E_{hydr}$ for the proposed pairs are -1.33 eV for Tm-Th, -1.34 eV for Yb-Th (Fig. S20c), -1.40 eV for Tm-Ca, and -1.43 eV for Yb-Ca (Fig. S21c). Our proposed pairs exhibit significantly more favorable hydration compared to Y-Ce: $\Delta E_{hydr}$= -0.17 eV for Tm-Th, -0.18 eV for Yb-Th, -0.25 eV for Tm-Ca, and -0.27 eV for Yb-Ca. Notably, the $E_{hydr}$ gains of -0.17 to -0.27 eV significantly outweigh the $E_{migr}$ increases of 0.01 to 0.11 eV. The enhancement in hydration performance substantially outweighs the modest increase in migration kinetics.

Moreover, the gains in $E_{hydr}$ becomes even more pronounced at practical operating temperatures (500–700°C). At elevated temperatures, proton diffusion becomes more facile as the increased thermal energy enables protons to readily overcome migration barriers. In contrast, dehydration becomes increasingly severe because the entropic contribution (TΔS) of the released H_2_O vapor increases with temperature, driving a substantial loss of proton concentration. For example, in BaZr_0.8_Y_0.2_O_3-δ_, the proton concentration remains at a maximum of 0.20 per unit cell up to 375°C but declines to 0.14 at 500°C (30% reduction) and to 0.05 at 700°C (75% reduction) [22]. Such a severe decrease in proton concentration directly reduces the number of charge carriers, making dehydration the dominant performance-limiting factor at operating temperatures [23,24]. Therefore, the $E_{hydr}$ gains of -0.17 to -0.27 eV substantially outweigh the modest $E_{migr}$ increases of 0.01 to 0.11 eV, as the enhanced hydration serves to suppress dehydration and maintain higher proton concentrations at elevated temperatures.

Furthermore, even in cases where protons interact strongly with dopant sites, Draber et al. demonstrated that at sufficient dopant concentrations, dopants can establish percolation pathways that enable protons to migrate between neighboring dopant sites without being trapped [25]. This suggests that proton trapping can be effectively mitigated through appropriate dopant concentrations, even when the local proton-dopant binding is strong.

Based on these considerations, we believe that the proposed co-doping pairs can exhibit higher proton conductivity than the conventional Y–Ce pair at elevated temperatures by maintaining sufficient proton concentration through enhanced hydration performance.

**P. Practical constraint of Thorium-containing materials due to its radioactivity**

The radioactivity of thorium (Th) raises challenges for practical PCO applications, including international regulations, radiation safety, and radioactive waste management. To address this concern, we evaluated non-radioactive alternatives with similar structural effects. Among the B-site isovalent dopants ($N_{Zr}^{X}$) examined, cerium (Ce) exhibits the second-highest improvement in hydration energy ($E_{hydr}$) after Th. Ce also has a larger ionic radius (0.87 Å) than Zr (0.72 Å), so it expands the free space at the A-site and reduces electrostatic repulsion between proton and A-site cation, thereby stabilizing protons. Our calculations show that for Yb-doped BaZrO_3_ (Yb-BZO), the $E_{hydr}$ decreases from -1.14 eV to -1.26 eV with Ce co-doping. Similarly, for Tm-BZO, the $E_{hydr}$ decreases from -1.13 eV to -1.25 eV with Ce co-doping. Although Ce provides a smaller enhancement compared to Th, it still offers a substantial improvement of 0.12 eV over acceptor-only doping while avoiding radioactivity concerns. Based on these results, we propose Th as a theoretical benchmark demonstrating the upper limit of $N_{Zr}^{X}$ co-doping effects, while recommending Ce as a practical, non-radioactive alternative for practical applications.


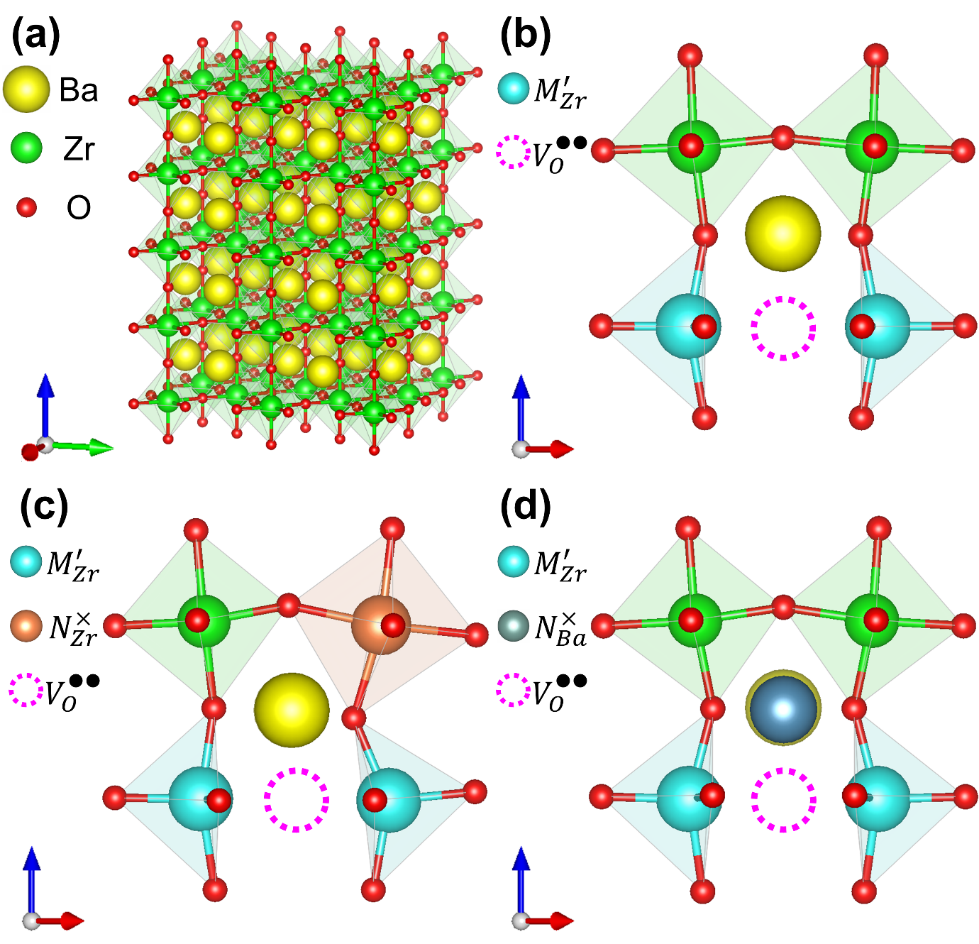


Figure S1. DFT-calculated structure of $2\sqrt{2}\times2\sqrt{2}\times4$ cubic pristine BaZrO_3_ (BZO) supercell containing 32 f.u. (160 atoms). Oxygen-deficient structures of (b) BZO with Zr sites substituted by two acceptors (M-BZO, $M_{Zr}^{'}$=Yb), (c) BZO with Zr sites substituted by two acceptors and one isovalent dopant (M,N-BZO, $M_{Zr}^{'}$=Yb, $N_{Zr}^{\times}$=Th), and (d) BZO with Zr sites substituted by two acceptors and Ba site substituted by one isovalent dopant (M,N-BZO, $M_{Zr}^{'}$=Yb, $N_{Ba}^{\times}$=Ca).


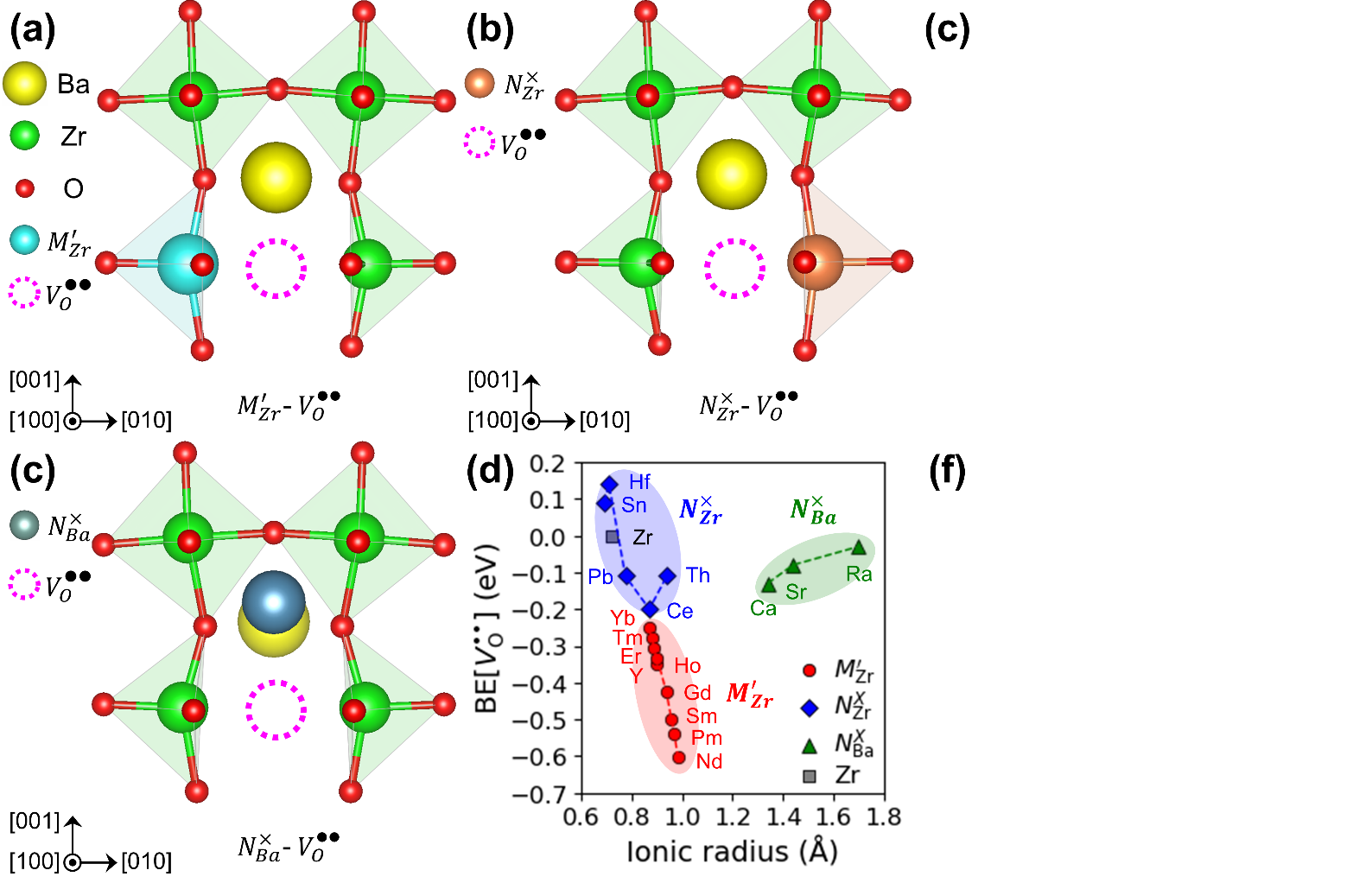


Figure S2. Oxygen-deficient structures of BaZrO_3_ (BZO) with (a) one acceptor at Zr site ($M_{Zr}^{'}$), (b) one isovalent dopant at Zr-site ($N_{Zr}^{\times}$), and (c) one isovalent dopant at Ba-site ($N_{Ba}^{\times}$). (d) Comparison of oxygen vacancy ($\text{V}_{\text{O}}$) binding energies (BE) with acceptor ($M_{Zr}^{'}$) and isovalent dopant ($N_{Zr}^{\times}$ or $N_{Ba}^{\times}$).


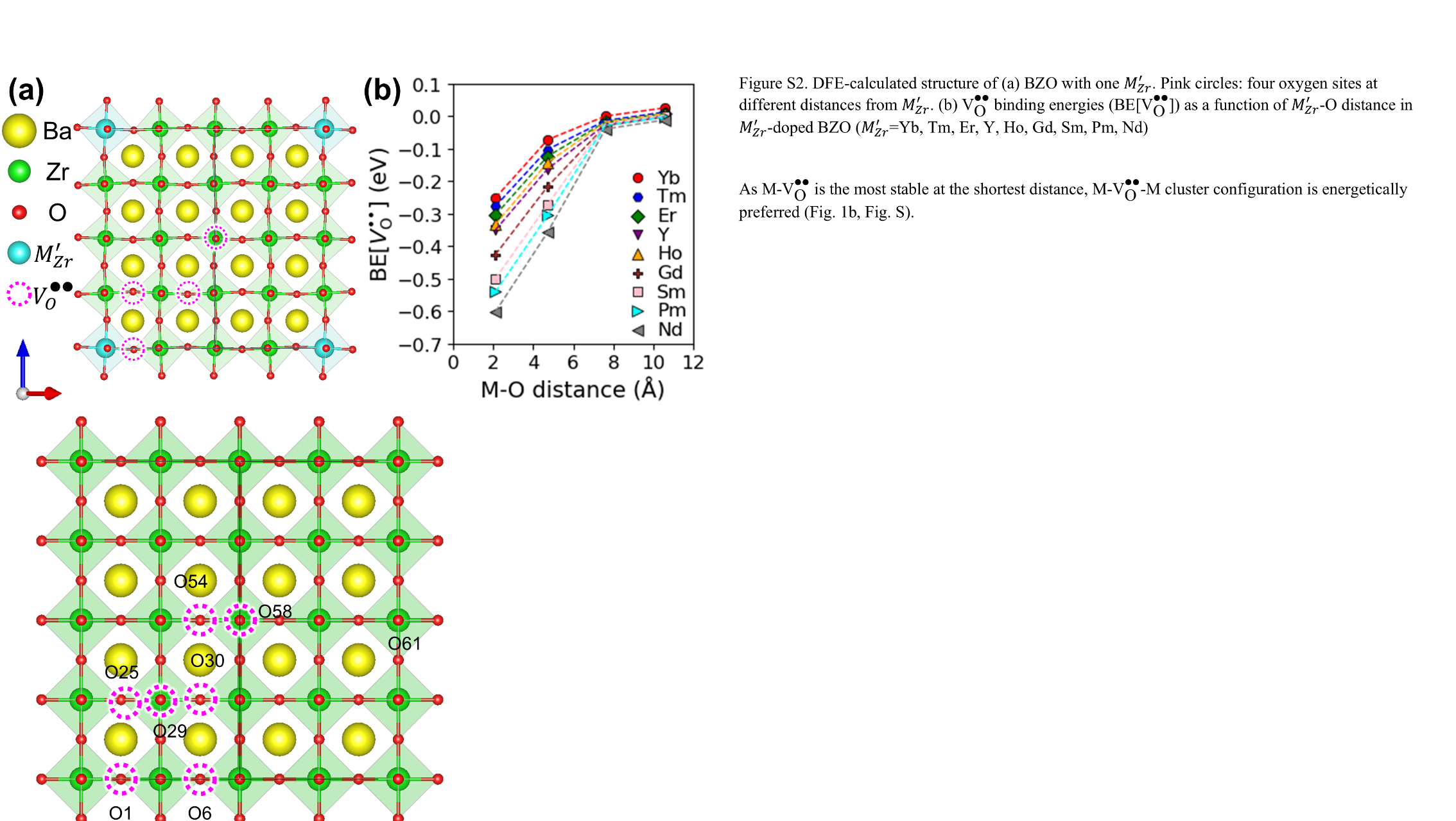


Figure S3. DFE-calculated structure of (a) BZO with one $M_{Zr}^{'}$. Pink circles: four oxygen sites at different distances from $M_{Zr}^{'}$. (b) $\text{V}_{\text{O}}$ binding energies (BE[$\text{V}_{\text{O}}$]) as a function of $M_{Zr}^{'}$-O distance in $M_{Zr}^{'}$-doped BZO ($M_{Zr}^{'}$=Yb, Tm, Er, Y, Ho, Gd, Sm, Pm, Nd)


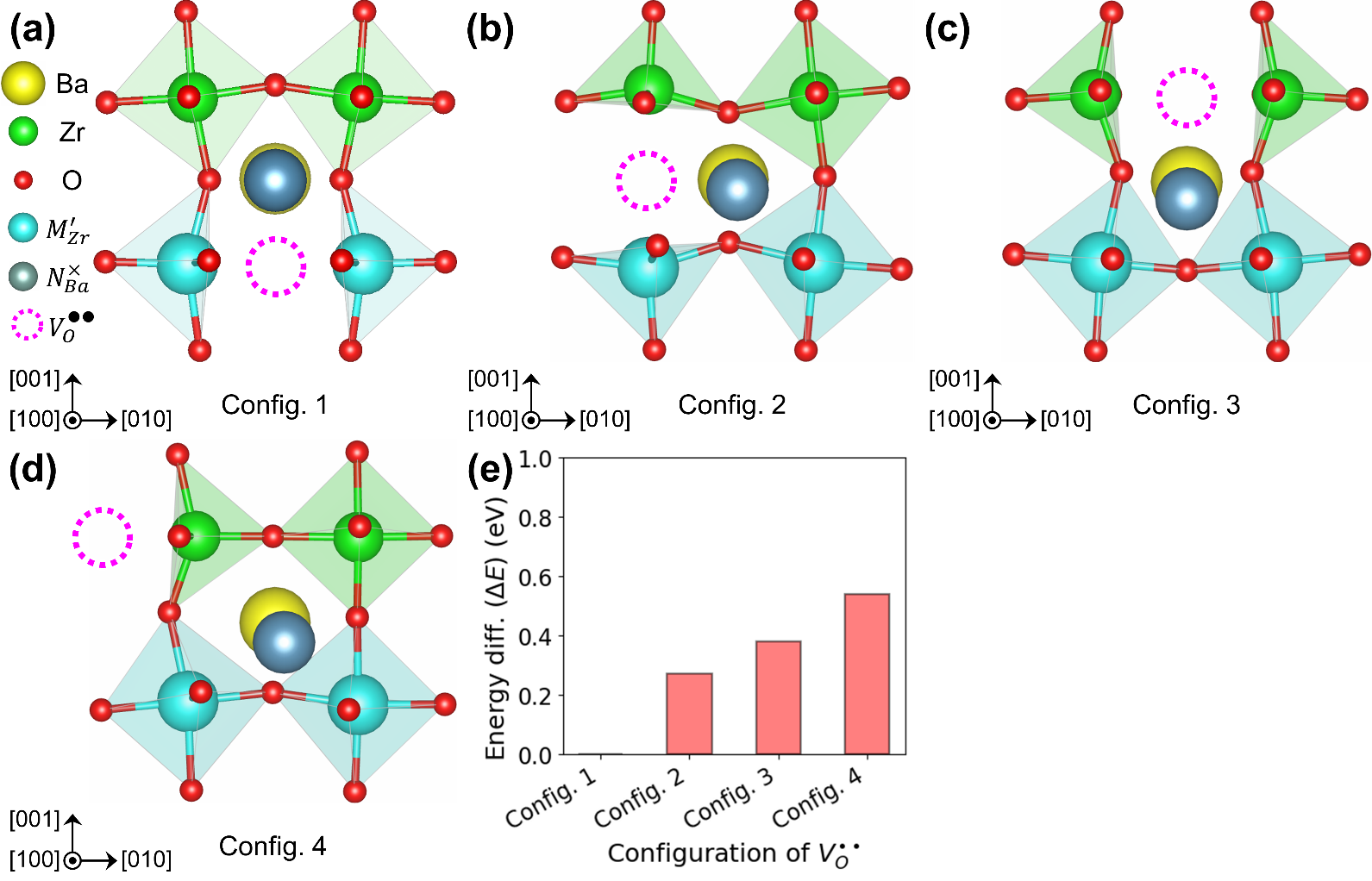


Figure S4. For A-site isovalent co-doped BZO (Ba_1-y_N_y_Zr_1-x_M_x_O_3_; $M_{Zr}^{'}$=Yb; $N_{Ba}^{\times}$=Ca; x=0.0625; y=0.03125), four configurations of $V_{O}$ (pink dashed circles) differing in the position of $V_{O}$: (a) configuration 1, (b) configuration 2, (c) configuration 3, (d) configuration 4. (e) Energy differences of configurations 2-4 relative to configuration 1.


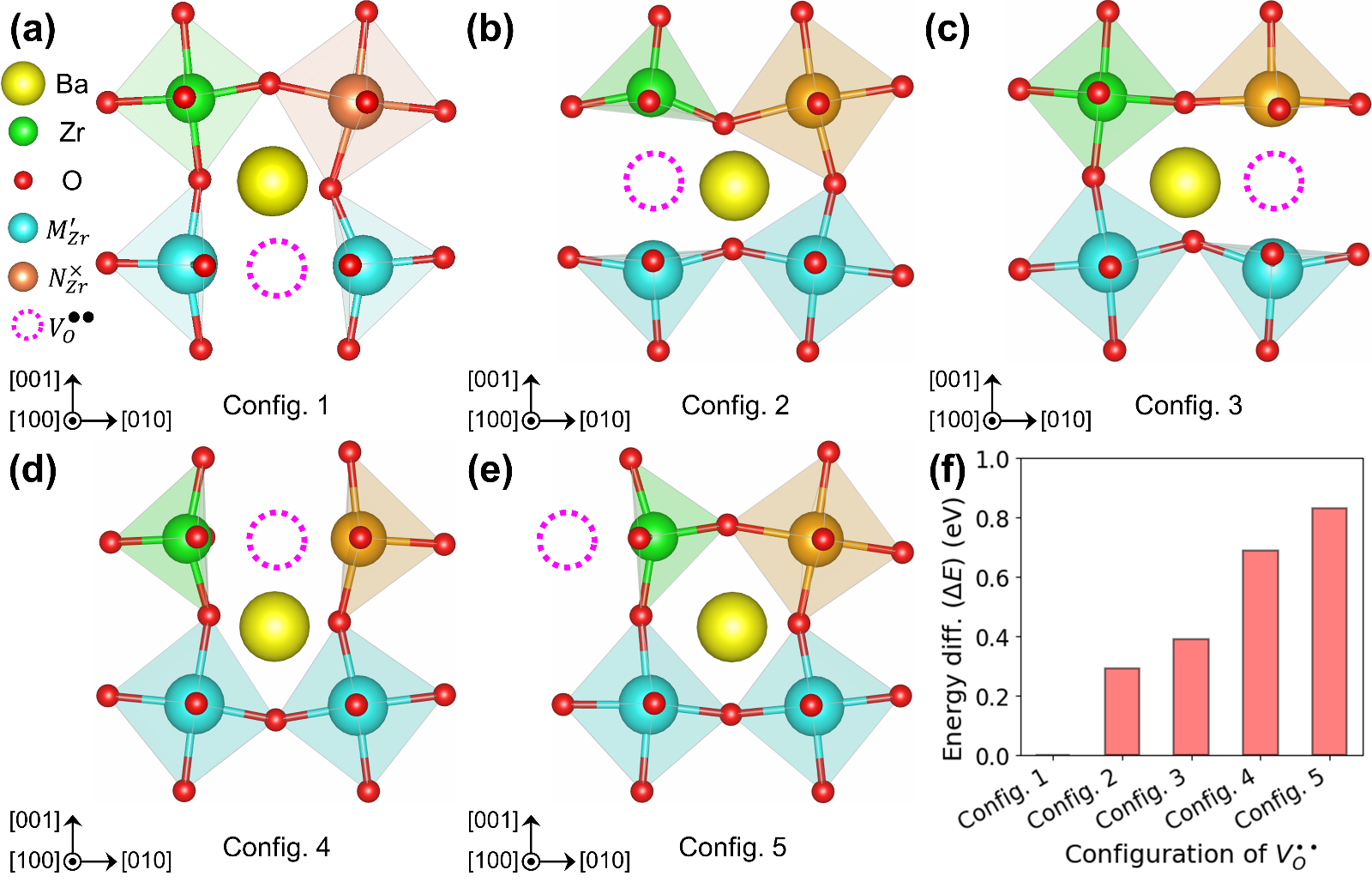


Figure S5. For B-site isovalent co-doped BZO (BaZr_1-x-y_-N_y_M_x_O_3_; $M_{Zr}^{'}$=Yb; $N_{Zr}^{\times}$=Th; x=0.0625; y=0.03125), five configurations of $V_{O}$ (pink dashed circles) differing in the position of $V_{O}$: (a) configuration 1, (b) configuration 2, (c) configuration 3, (d) configuration 4, (e) configuration 5. (f) Energy differences of configurations 2-5 relative to configuration 1.


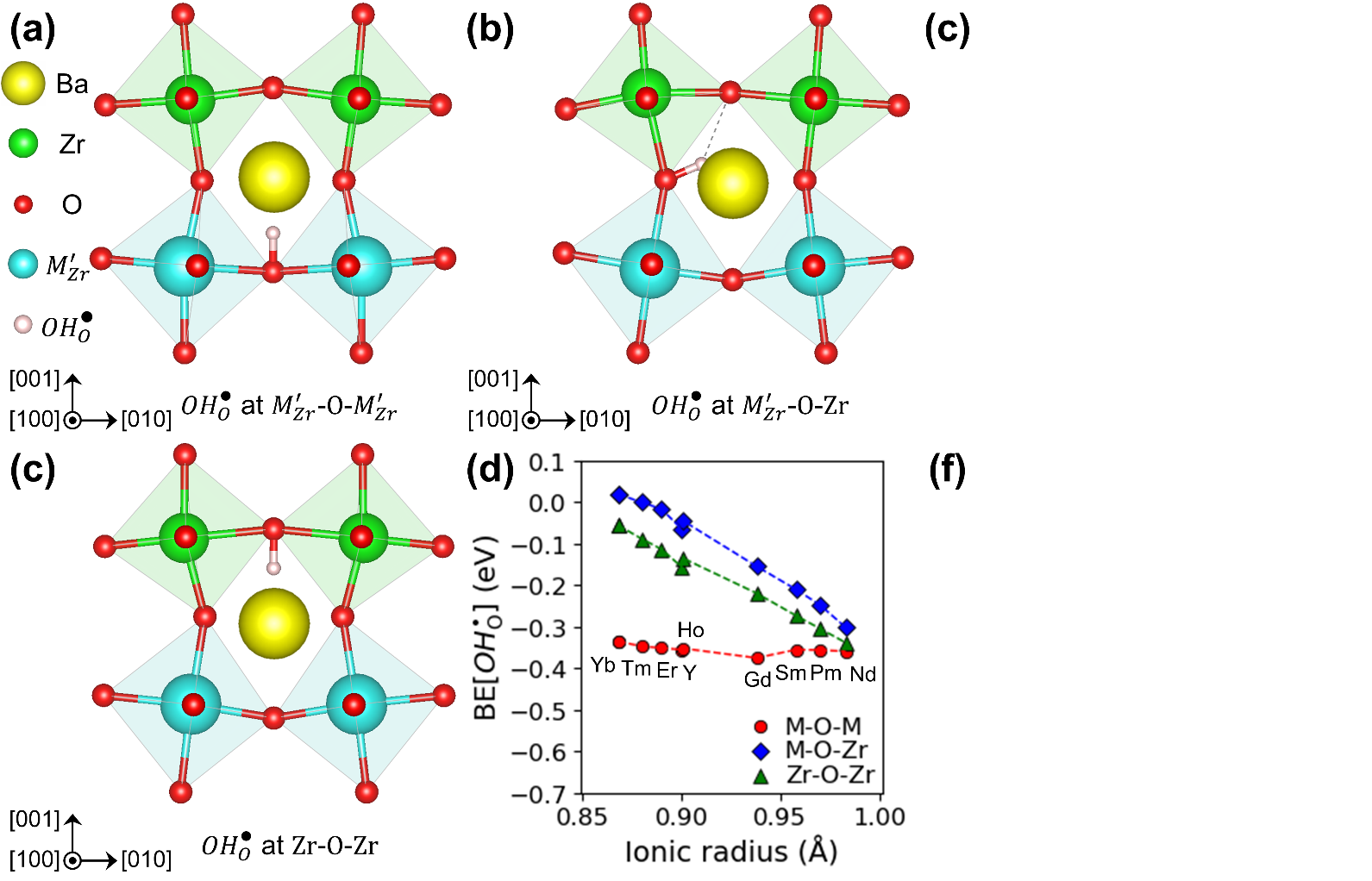


Figure S6. DFE-calculated structures of BZO with two $M_{Zr}^{'}$ (M-BZO) showing three different binding sites of single ${OH}_{O}$: one ${OH}_{O}$ at (a) M-O-M, (b) M-O-Zr, and (c) Zr-O-Zr. A-site cations omitted for clarity. (d) ${OH}_{O}$ binding energies (BE[${OH}_{O}$ ]) as a function of $M_{Zr}^{'}$ ionic radius in M-BZO ($M_{Zr}^{'}$=Yb, Tm, Er, Y, Ho, Gd, Sm, Pm, Nd)


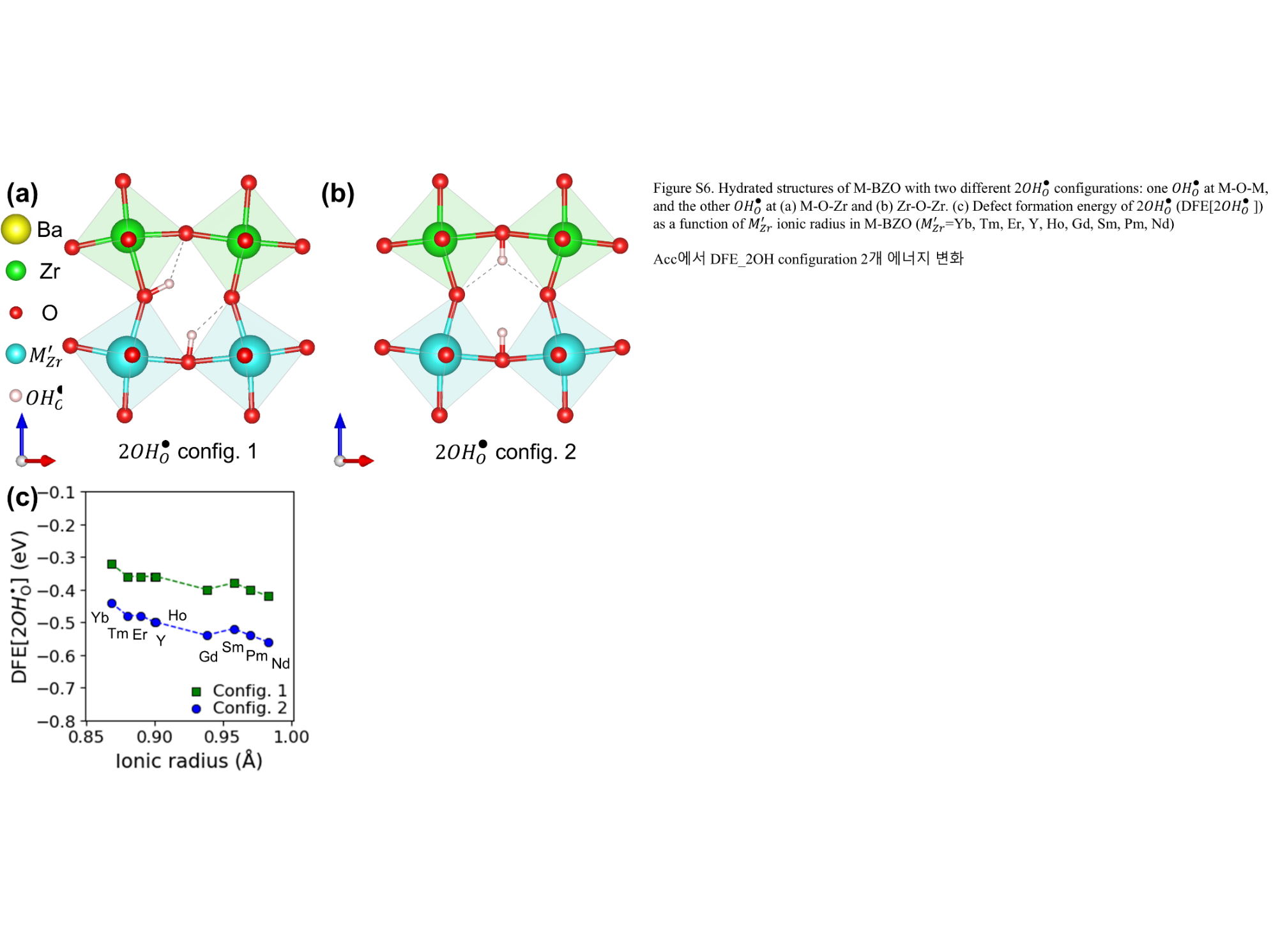


Figure S7. For M-BZO ($M_{Zr}^{'}$=Yb, Tm, Er, Y, Ho, Gd, Sm, Pm, Nd), hydrated structures with two different configurations of 2${OH}_{O}$: one ${OH}_{O}$ at M-O-M, and the other ${OH}_{O}$ at (a) M-O-Zr and (b) Zr-O-Zr. A-site cations omitted for clarity. (c) Defect formation energy of 2${OH}_{O}$ (DFE[2${OH}_{O}$ ]) as a function of $M_{Zr}^{'}$ ionic radius in M-BZO


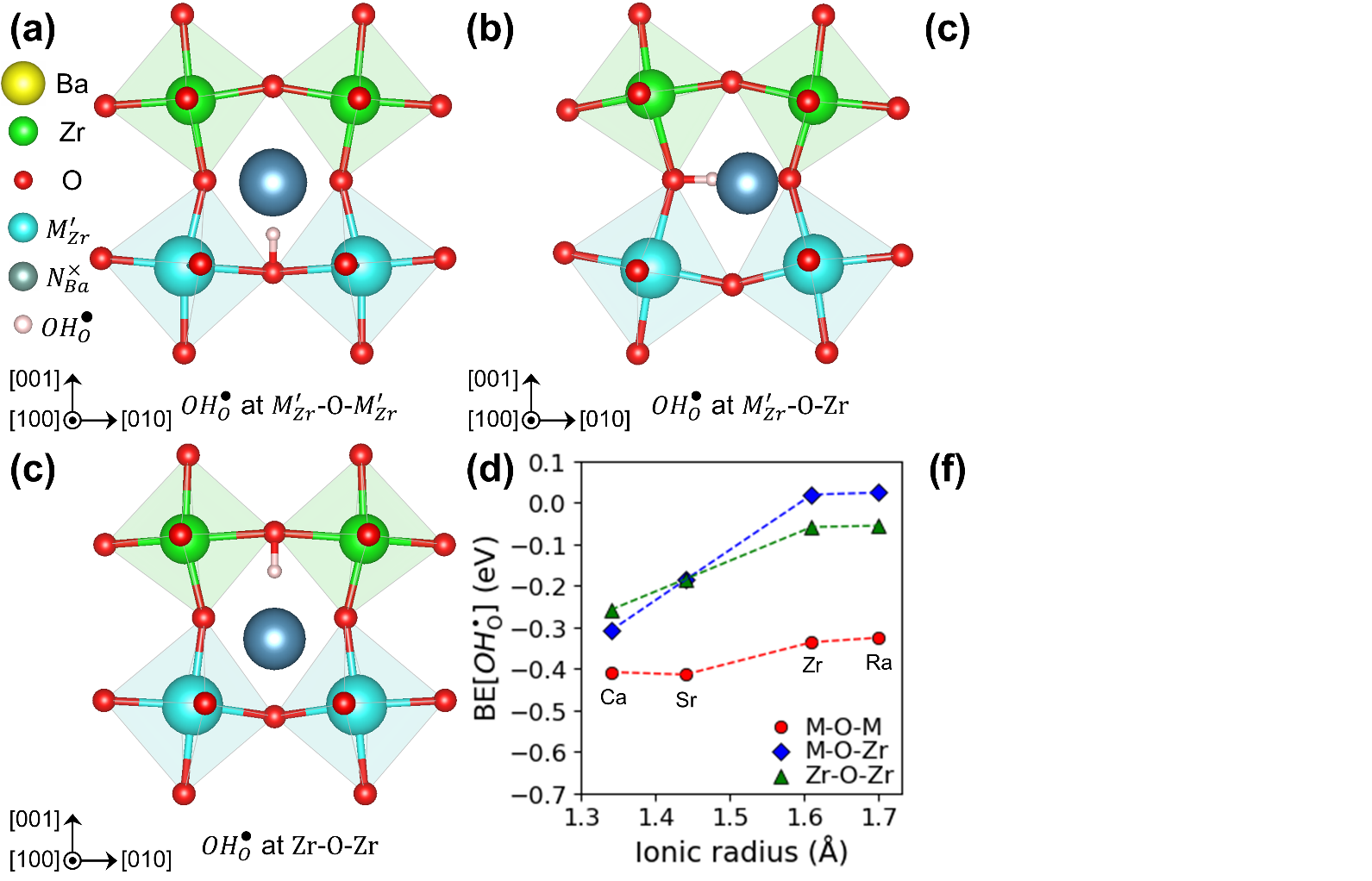


Figure S8. DFT-calculated structures of two $M_{Zr}^{'}$ and one $N_{Ba}^{\times}$ co-doped BZO (M,N-BZO) with three different binding sites of single ${OH}_{O}$: one ${OH}_{O}$ at (a) M-O-M, (b) M-O-Zr, and (c) Zr-O-Zr. (d) BE[${OH}_{O}$ ] as a function of $N_{Ba}^{\times}$ ionic radius in M,N-BZO ($M_{Zr}^{'}$=Yb and $N_{Ba}^{\times}$= Ca, Sr, Zr, Ra)


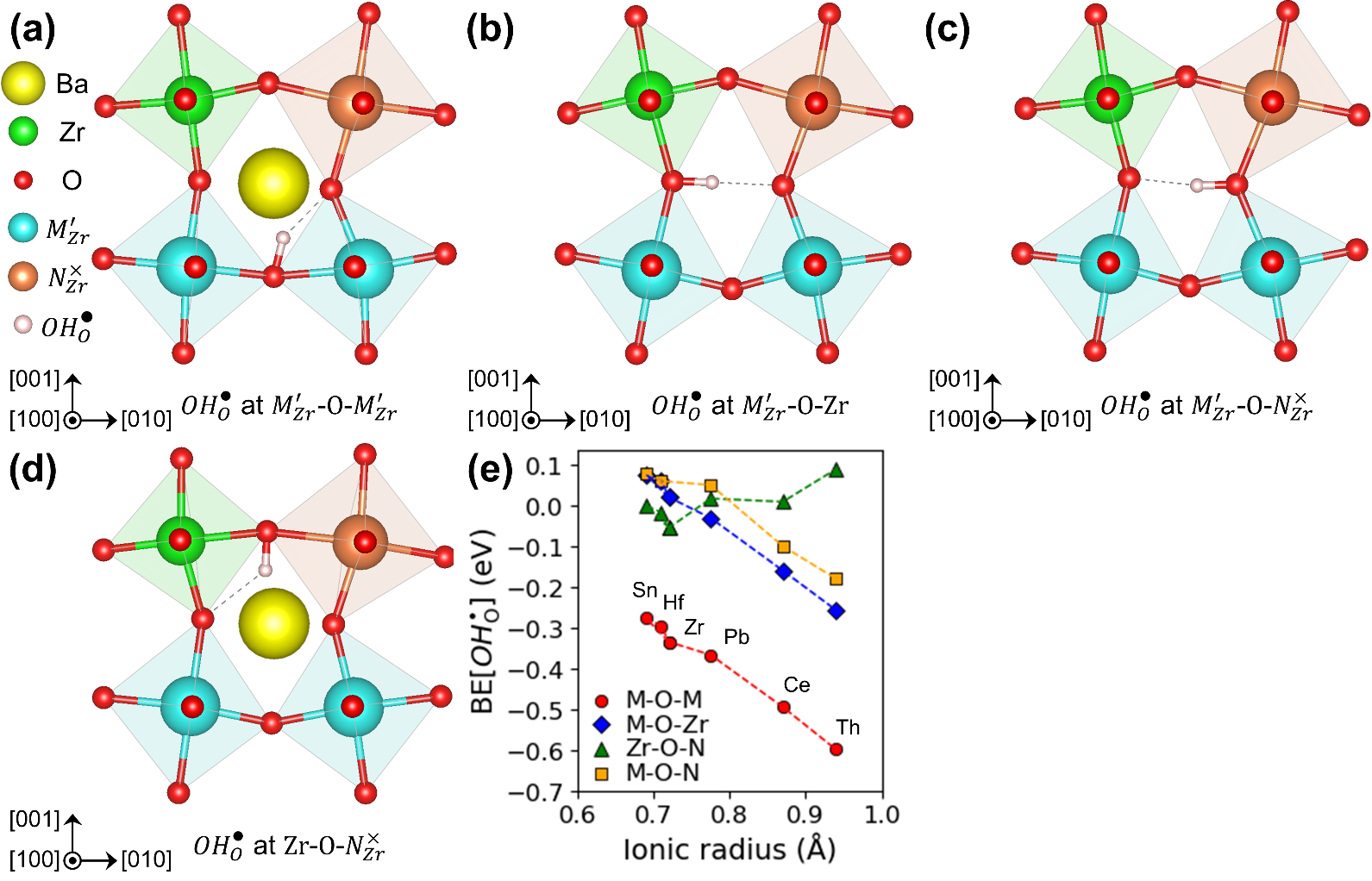


Figure S9. DFE-calculated structures of two $M_{Zr}^{'}$ and one $N_{Zr}^{\times}$ co-doped BZO (M,N-BZO) with four different binding sites of single ${OH}_{O}$: one ${OH}_{O}$ at (a) M-O-M, (b) M-O-Zr, (c) Zr-O-N, and (d) M-O-N. A-site cations omitted for clarity. (e) BE[${OH}_{O}$ ] as a function of $N_{Zr}^{\times}$ ionic radius in M,N-BZO ($M_{Zr}^{'}$=Yb and $N_{Zr}^{\times}$=Sn, Hf, Zr, Pb, Ce, Th)


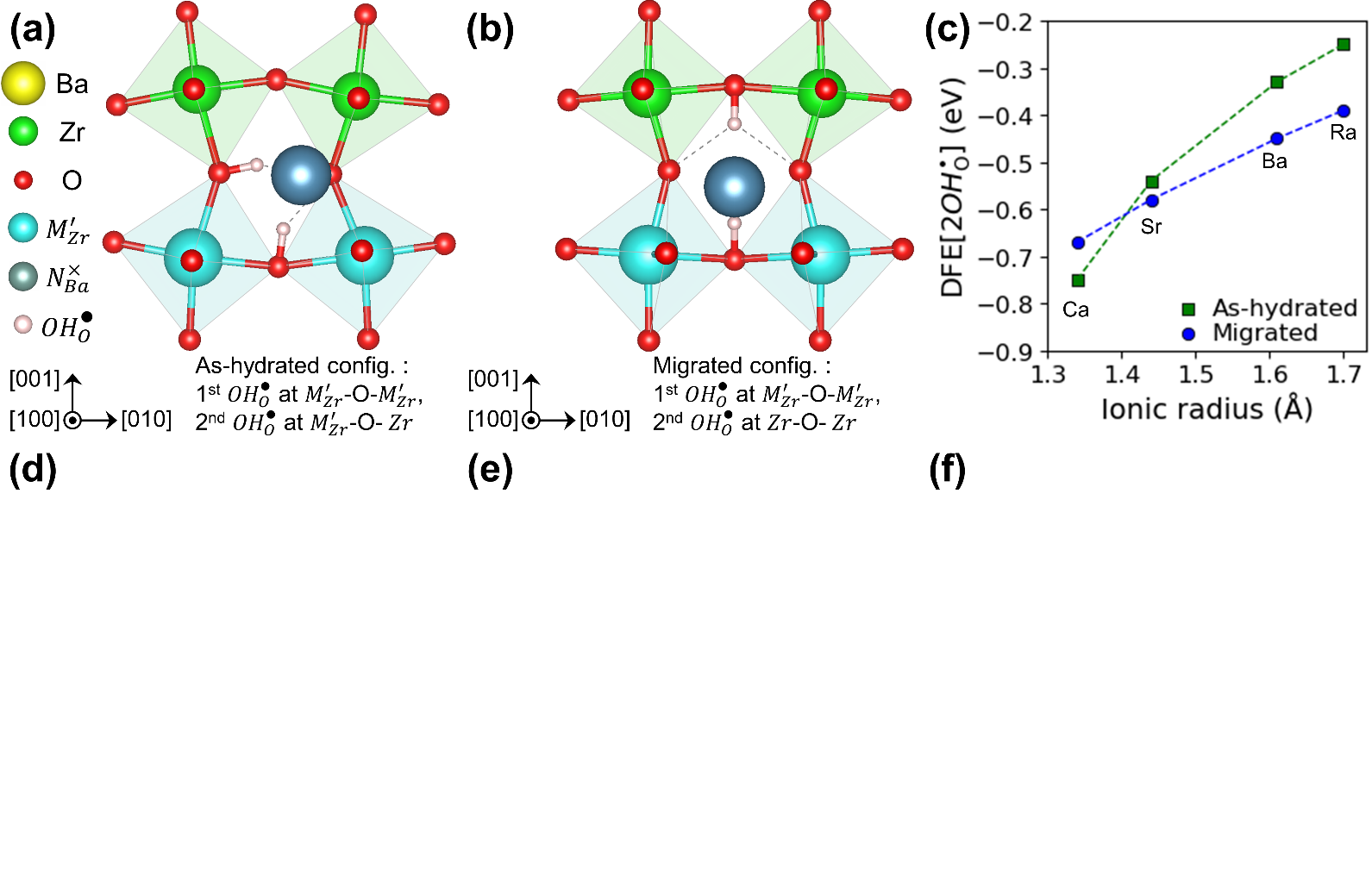


Figure S10. For M,N-BZO ($M_{Zr}^{'}$=Yb and $N_{Ba}^{\times}$=Ca, Sr, Ba, Ra), hydrated structures with two different configurations of 2${OH}_{O}$: one ${OH}_{O}$ at M-O-M, and the other ${OH}_{O}$ at (a) M-O-Zr (As-hydrated configuration) and (b) Zr-O-Zr (Migrated configuration). (c) DFE[2${OH}_{O}$] as a function of $N_{Ba}^{\times}$ ionic radius in M,N-BZO


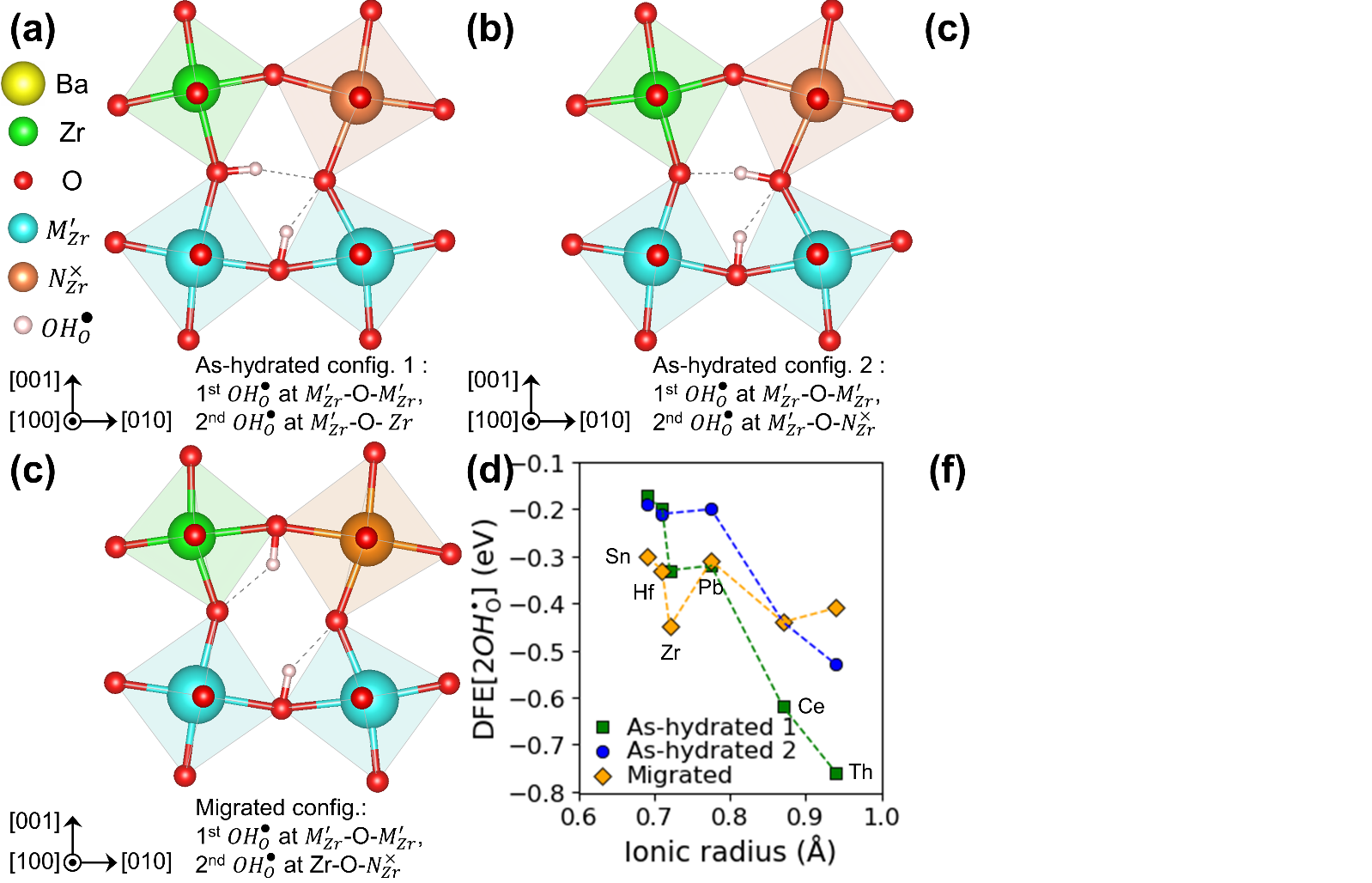


Figure S11. For M,N-BZO ($M_{Zr}^{'}$=Yb and $N_{Zr}^{\times}$=Sn, Hf, Zr, Pb, Ce, Th), hydrated structures with three different configurations of 2${OH}_{O}$: one ${OH}_{O}$ at M-O-M, and the other ${OH}_{O}$ at (a) M-O-Zr (As-hydrated configuration 1), (b) M-O-N (As-hydrated configuration 2), and (c) Zr-O-N (Migrated configuration). A-site cations omitted for clarity. (d) DFE[2${OH}_{O}$] as a function of $N_{Zr}^{\times}$ ionic radius in M,N-BZO


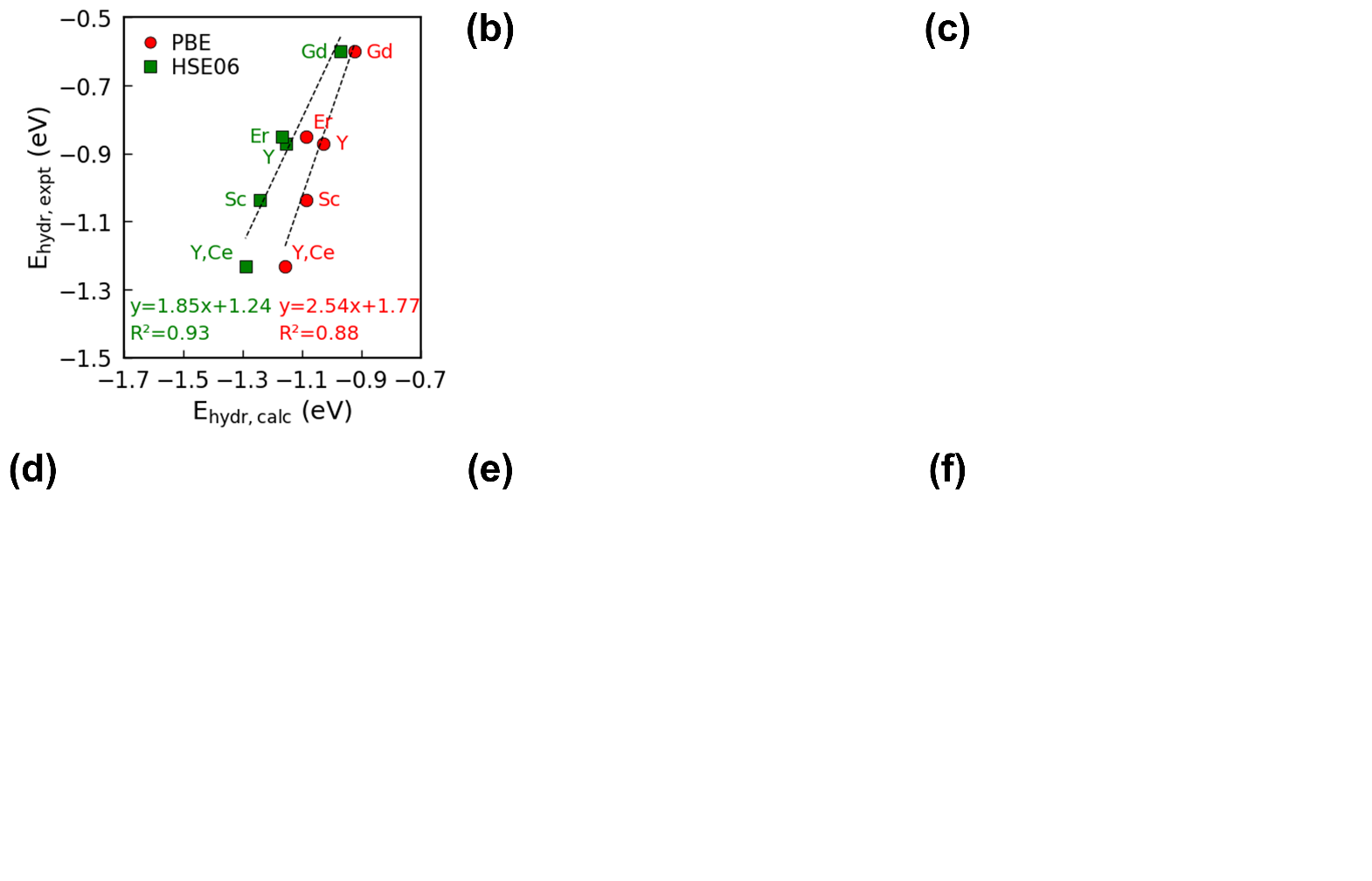


Figure S12. Comparison between calculated ($E_{hydr,calc}$) and experimental hydration energies ($E_{hydr, expt}$) for BaZr_1-x-y_M_x_N_y_O_3_. $E_{hydr,calc}$ were obtained using HSE06 (green squares, R² = 0.93) and PBE (red circles, R² = 0.88) functionals. $M_{Zr}^{'}$ = Gd, Er, Y, Sc with $N_{Zr}^{\times}$=Zr; $M_{Zr}^{'}$=Y with $N_{Zr}^{\times}$=Ce; x=0.0625, y=0.03125 for $E_{hydr,calc}$; x=0.2, y=0.2 for $E_{hydr, expt}$.


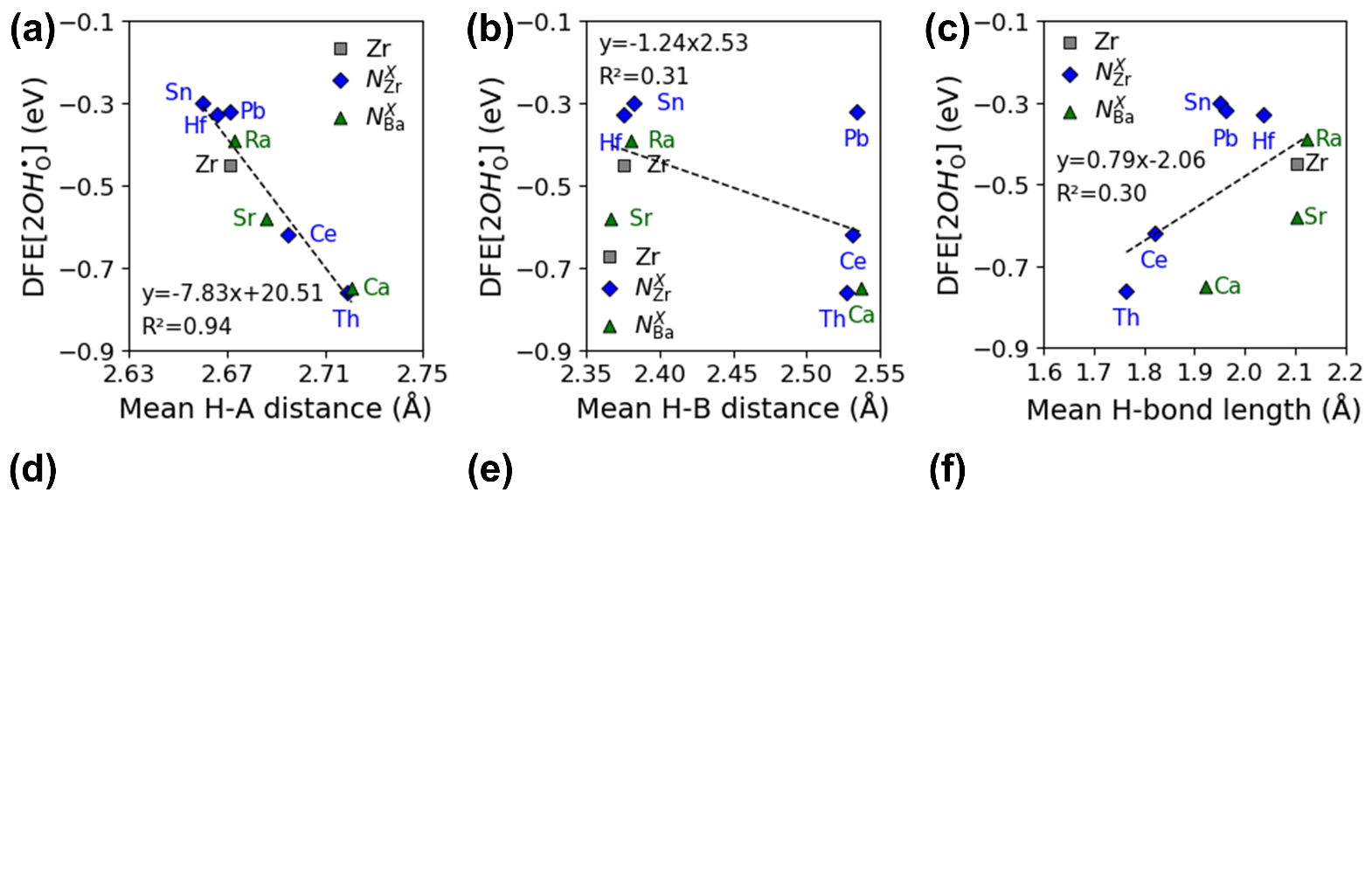


Figure S13. For M,N-BZO ($M_{Zr}^{'}$=Yb and $N_{Zr}^{\times}$= Sn, Hf, Zr, Pb, Ce, Th or $N_{Ba}^{\times}$= Ra, Sr, Ca), correlation between DFE[$2{OH}_{O}$] and (a) mean of four distances between $2{OH}_{O}$and their two nearest A-site cations (mean H-A distance, R² = 0.94), (b) mean of four distances between $2{OH}_{O}$and their two nearest $M_{Zr}^{'}$ (mean H-B distance, R² = 0.31), and (c) mean hydrogen bond of $2{OH}_{O}$ (mean H-bond length, R² = 0.30).


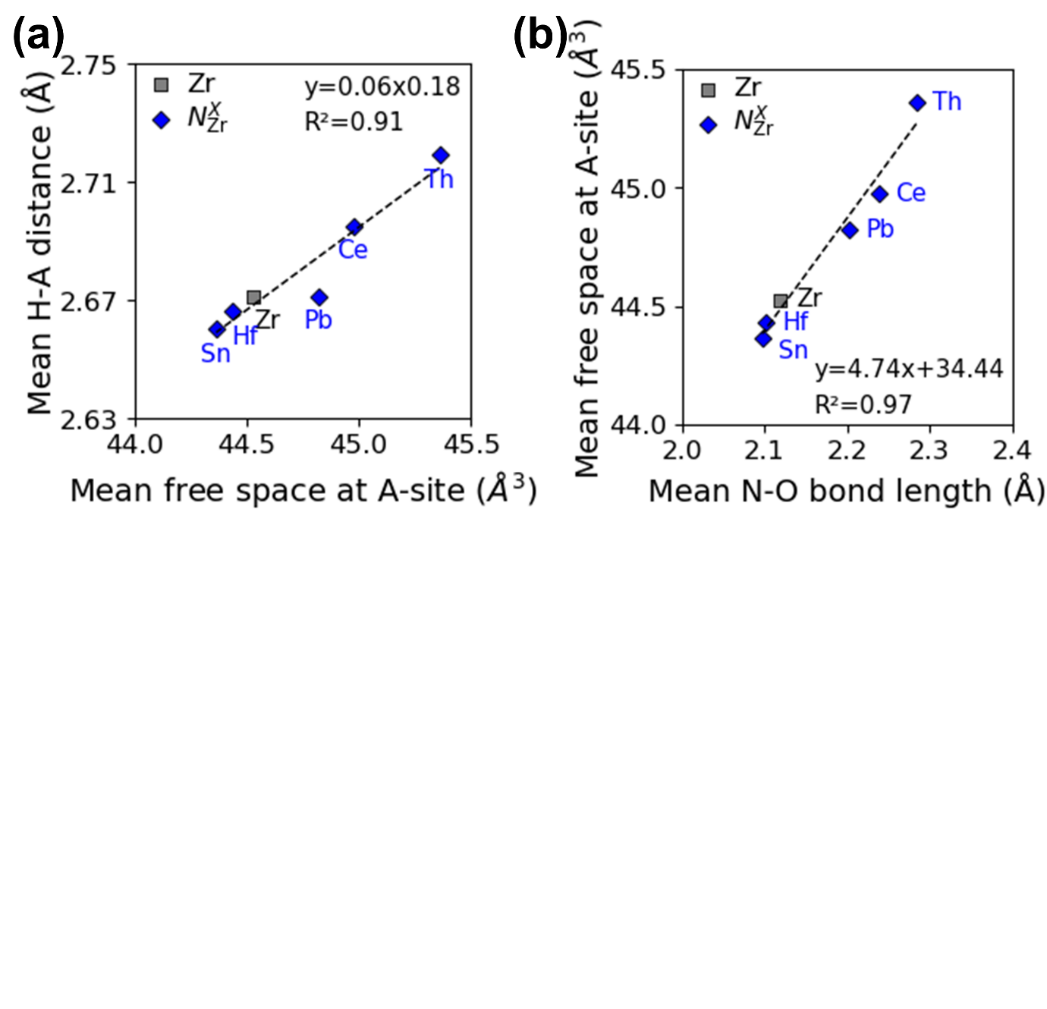


Figure S14. For M,N-BZO ($M_{Zr}^{'}$=Yb and $N_{Zr}^{\times}$= Sn, Hf, Zr, Pb, Ce, Th): (a) Correlation between mean H-A distance and mean free space at A-site. The mean free space is defined as the difference between the mean volume of two AO12 polyhedra nearest to the $2{OH}_{O}$ and the mean volume of two A-site cations, where each AO12 polyhedron is formed by an A-site cation and its twelve coordinating oxide ions. (b) Correlation between mean free space at A-site and mean N-O bond length, averaged over six N-O bond lengths.


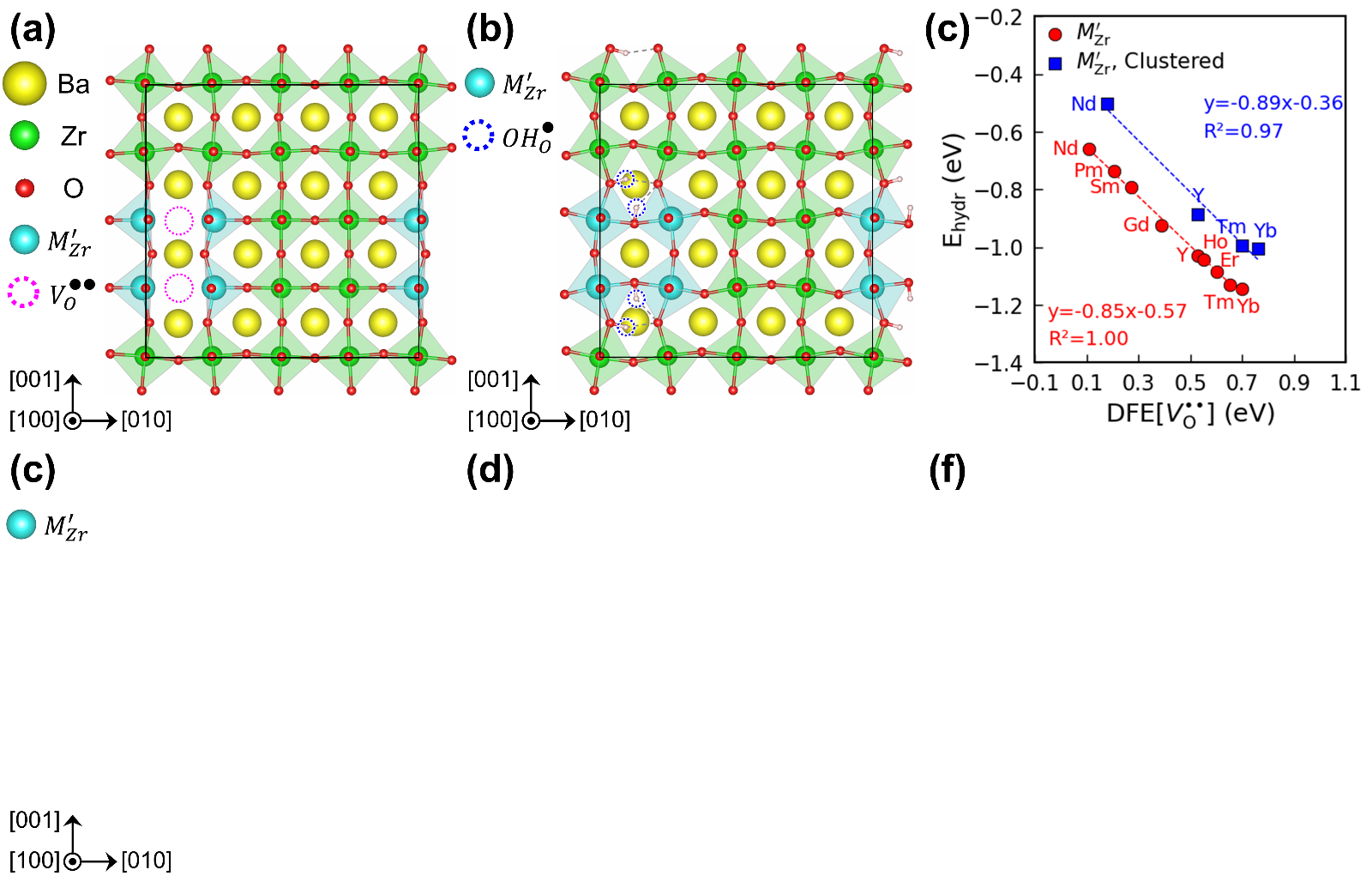


Figure S15. Clustered configurations of four acceptors in acceptor-doped BZO (BaZr_1-x_M_x_O_3_; $M_{Zr}^{'}$=Nd, Y, Tm, Yb; x=0.125). (a) Oxygen-deficient structure containing two $V_{O}$ (pink dashed circle), each located between two $M_{Zr}^{'}$, and (b) hydrated structure with four ${OH}_{O}$ (blue dashed circle). (c) Comparison of defect formation energy (DFE) of oxygen vacancy ($\text{V}_{\text{O}}$) and hydration energy ($E_{hydr}$) for x = 0.0625 (R^2^=1.00) and x = 0.125 (R^2^=0.97).


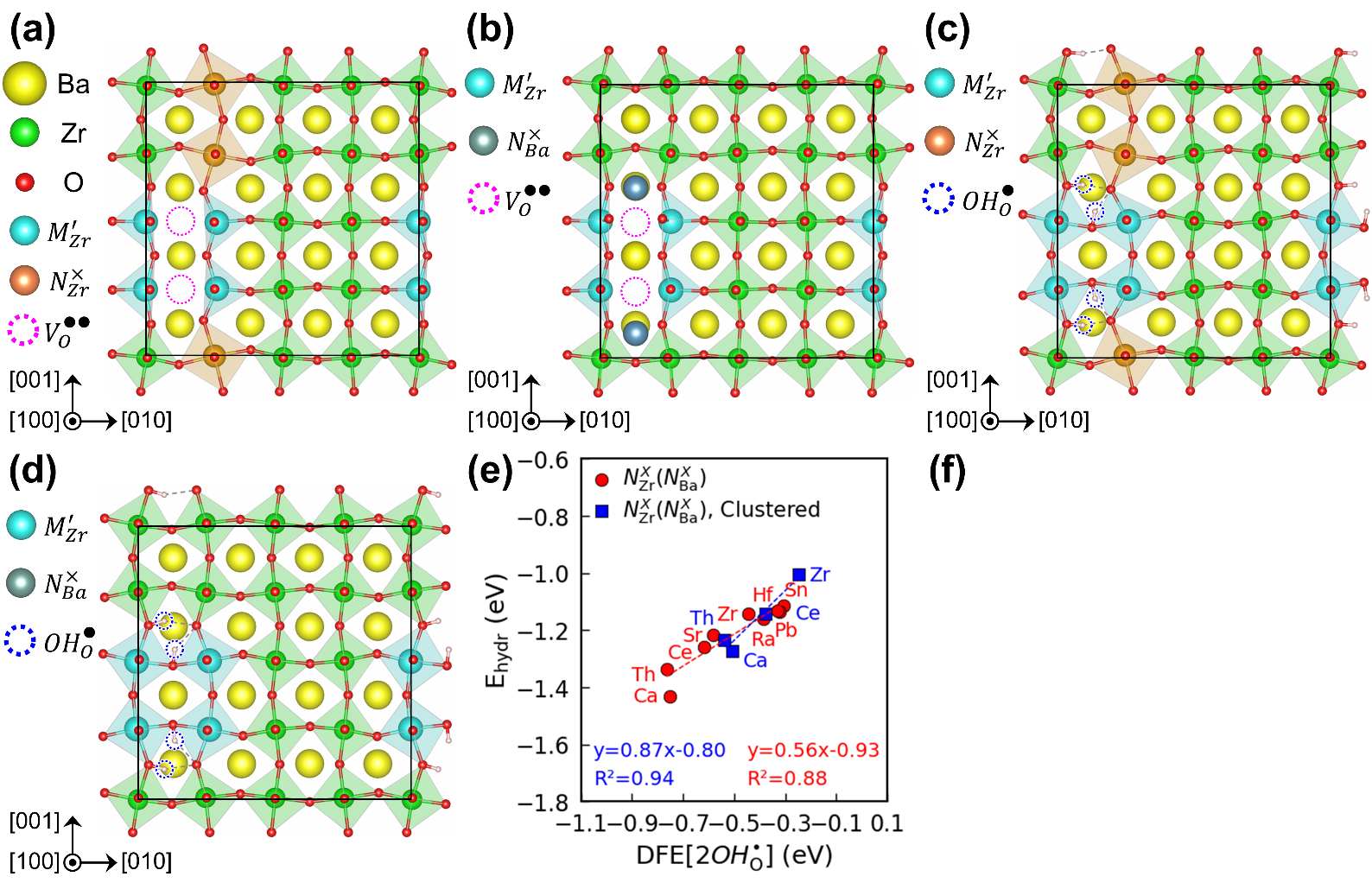


Figure S16. Clustered configurations of four acceptors in isovalent co-doped BZO at x = 0.125 and y = 0.0625. (a, b) Oxygen-deficient structure containing two $V_{O}$ (pink dashed circle), each located between two $M_{Zr}^{'}$, for (a) B-site isovalent co-doped BZO (BaZr_1-x-y_-N_y_M_x_O_3_; $M_{Zr}^{'}$=Yb; $N_{Zr}^{\times}$=Th, Ce; x=0.125; y=0.0625) and (b) A-site isovalent co-doped BZO (Ba_1-y_N_y_Zr_1-x_M_x_O_3_; $M_{Zr}^{'}$=Yb; $N_{Ba}^{\times}$=Ca; x=0.125; y=0.0625). (c, d) Corresponding hydrated structure with four ${OH}_{O}$ (blue dashed circle) for (c) B-site isovalent co-doped BZO and (d) A-site isovalent co-doped BZO. (e) Comparison of defect formation energy (DFE) of two protons ($2\text{OH}_{\text{O}}$) and hydration energy ($E_{hydr}$) at x = 0.0625, y=0.03125 (R^2^=0.88) and x = 0.125, y=0.0625 (R^2^=0.94).


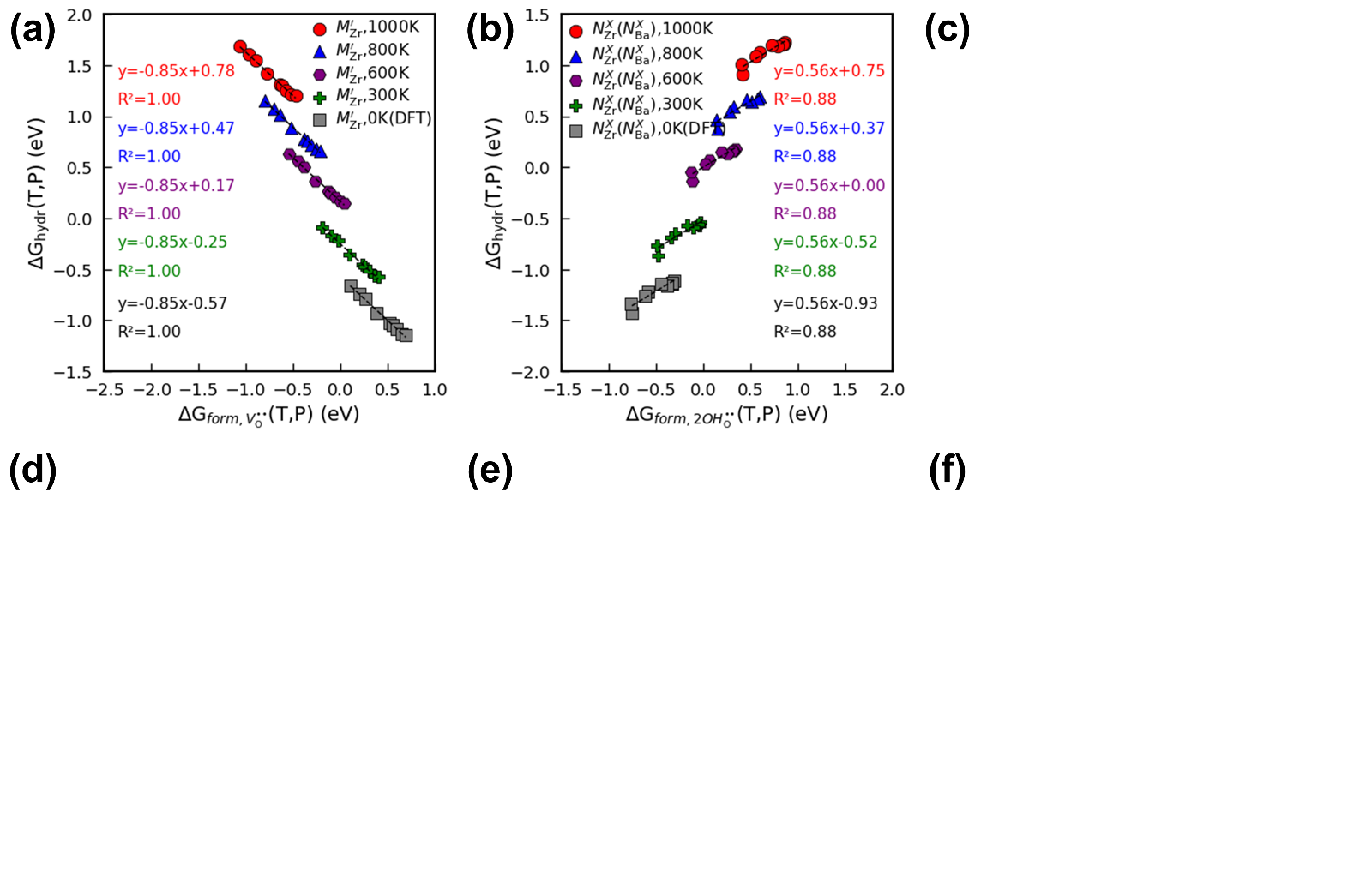


Figure S17. (a) For M-doped BZO ($M_{Zr}^{'}$= Nd, Pm, Sm, Gd, Y, Ho, Er, Tm, Yb), (a) correlation between oxygen vacancy ($V_{O}$) formation free energy ($\Delta G_{form, V_{O}}$) and hydration free energy ($\Delta G_{hydr}$) at 0K (R² = 1.00), 300K (R² = 1.00), 600K (R² = 1.00), 800K (R² = 1.00), and 1000K (R² = 1.00). (b) For Yb, N-BZO ($N_{Zr}^{\times}$= Zr, Sn, Pb, Hf, Ce, Th, and $N_{Ba}^{\times}$= Ra, Sr, Ca), correlation between protons (${OH}_{O}$) formation free energy ($\Delta G_{form,2{OH}_{O}}$) and hydration free energy ($\Delta G_{hydr}$) at 0K (R² = 0.88), 300K (R² = 0.88), 600K (R² = 0.88), 800K (R² = 0.88), and 1000K (R² = 0.88).


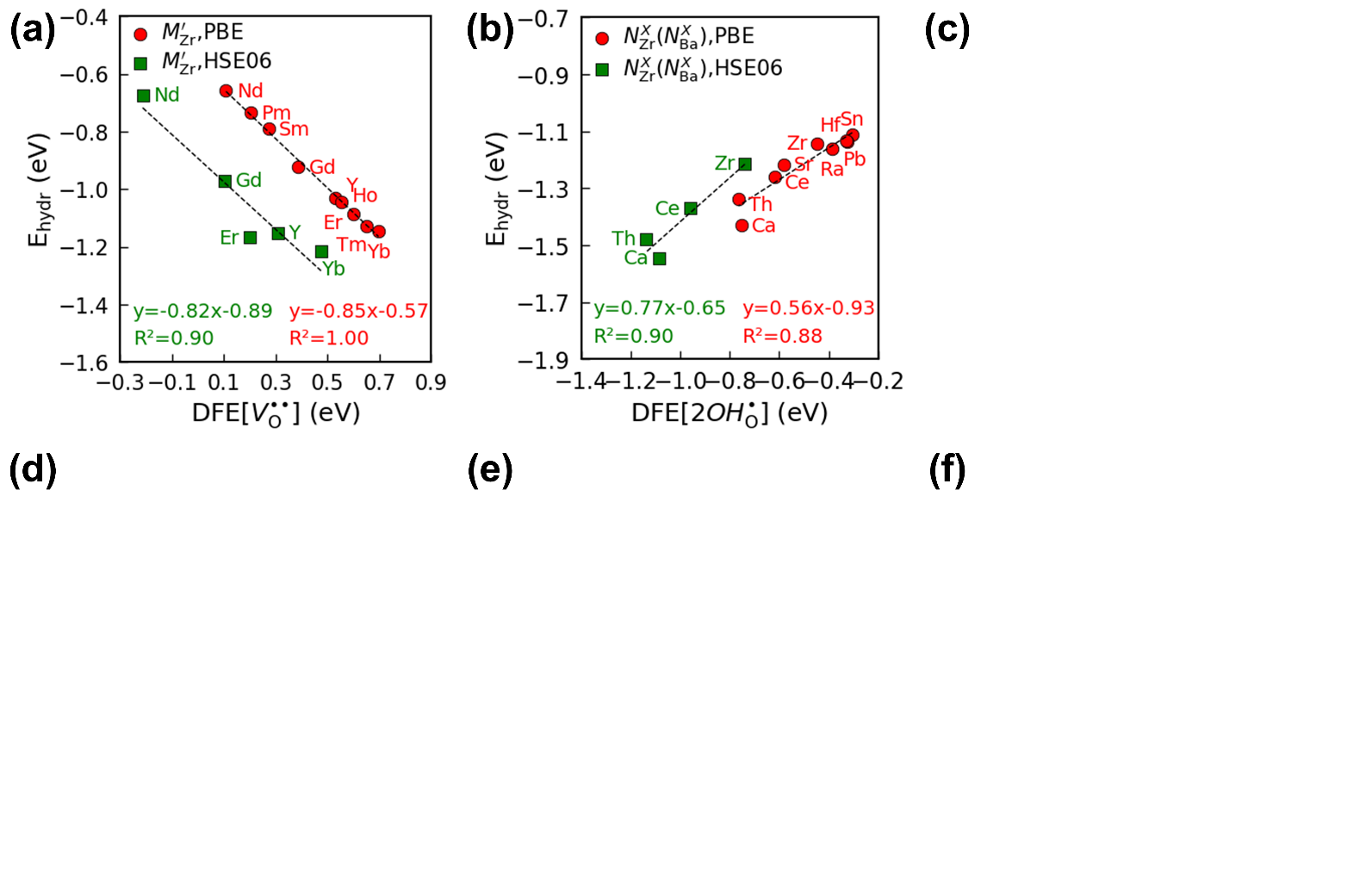


Figure S18. (a) For M-doped BZO ($M_{Zr}^{'}$= Nd, Pm, Sm, Gd, Y, Ho, Er, Tm, Yb), correlation between defect formation energy (DFE) of an oxygen vacancy ($V_{O}$) (DFE[$V_{O}$]) and hydration energy ($E_{hydr}$). PBE: red circles, R²=1.00; HSE: blue squares, R²=0.90. (b) For Yb, N-BZO ($N_{Zr}^{\times}$= Zr, Sn, Pb, Hf, Ce, Th, and $N_{Ba}^{\times}$= Ra, Sr, Ca), correlation between defect formation energy (DFE) of two protons ($2{OH}_{O}$) (DFE[$2{OH}_{O}$]) and $E_{hydr}$. PBE: red circles, R²=0.88; HSE: blue squares, R²=0.90


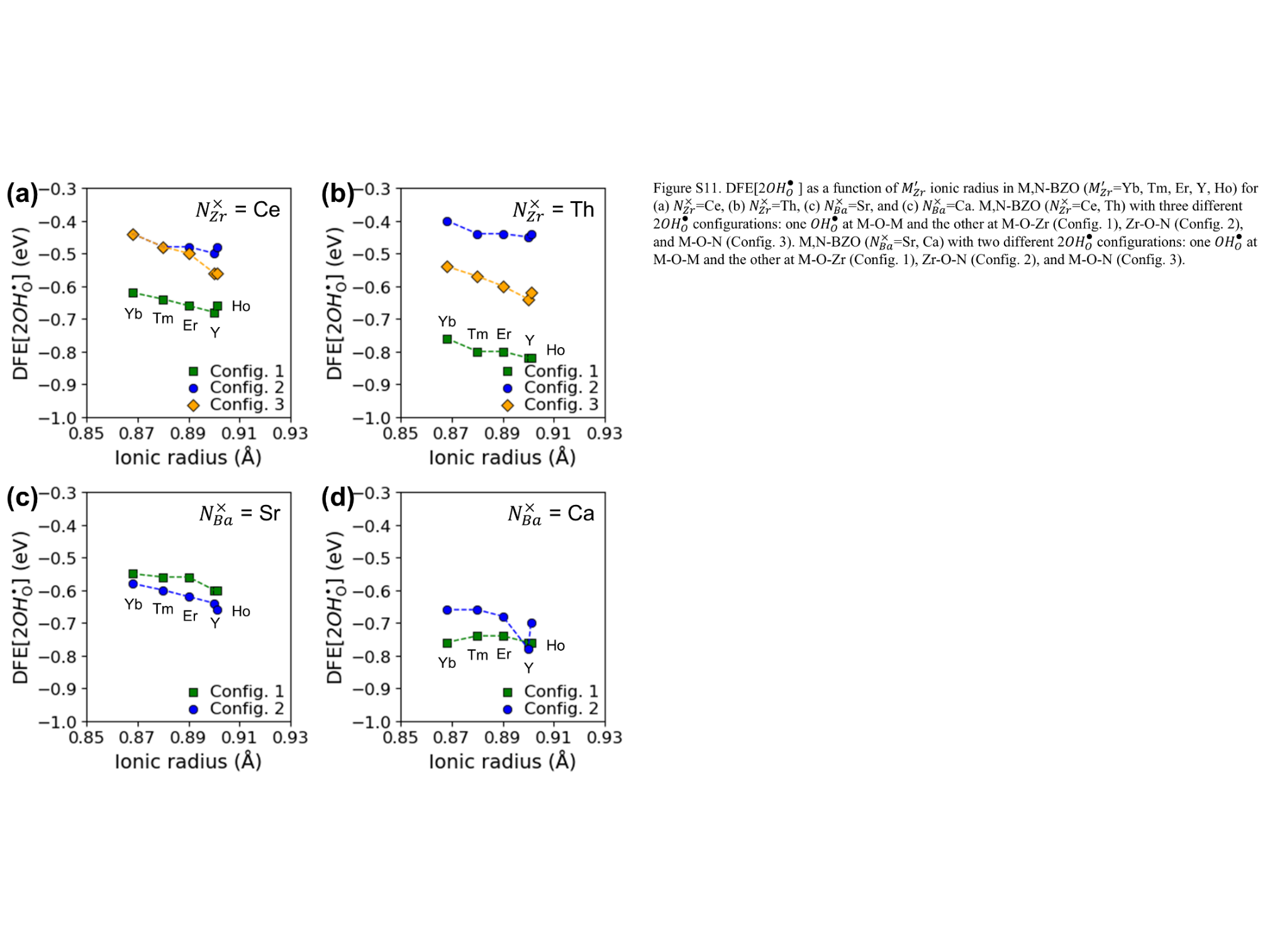


Figure S19. DFE[2${OH}_{O}$ ] as a function of $M_{Zr}^{'}$ ionic radius in M,N-BZO ($M_{Zr}^{'}$=Yb, Tm, Er, Y, Ho) for (a) $N_{Zr}^{\times}$=Ce, (b) $N_{Zr}^{\times}$=Th, (c) $N_{Ba}^{\times}$=Sr, and (c) $N_{Ba}^{\times}$=Ca. M,N-BZO ($N_{Zr}^{\times}$=Ce, Th) with three different 2${OH}_{O}$ configurations: one ${OH}_{O}$ at M-O-M and the other at M-O-Zr (Config. 1), Zr-O-N (Config. 2), and M-O-N (Config. 3). M,N-BZO ($N_{Ba}^{\times}$=Sr, Ca) with two different 2${OH}_{O}$ configurations: one ${OH}_{O}$ at M-O-M and the other at M-O-Zr (Config. 1), Zr-O-N (Config. 2), and M-O-N (Config. 3).


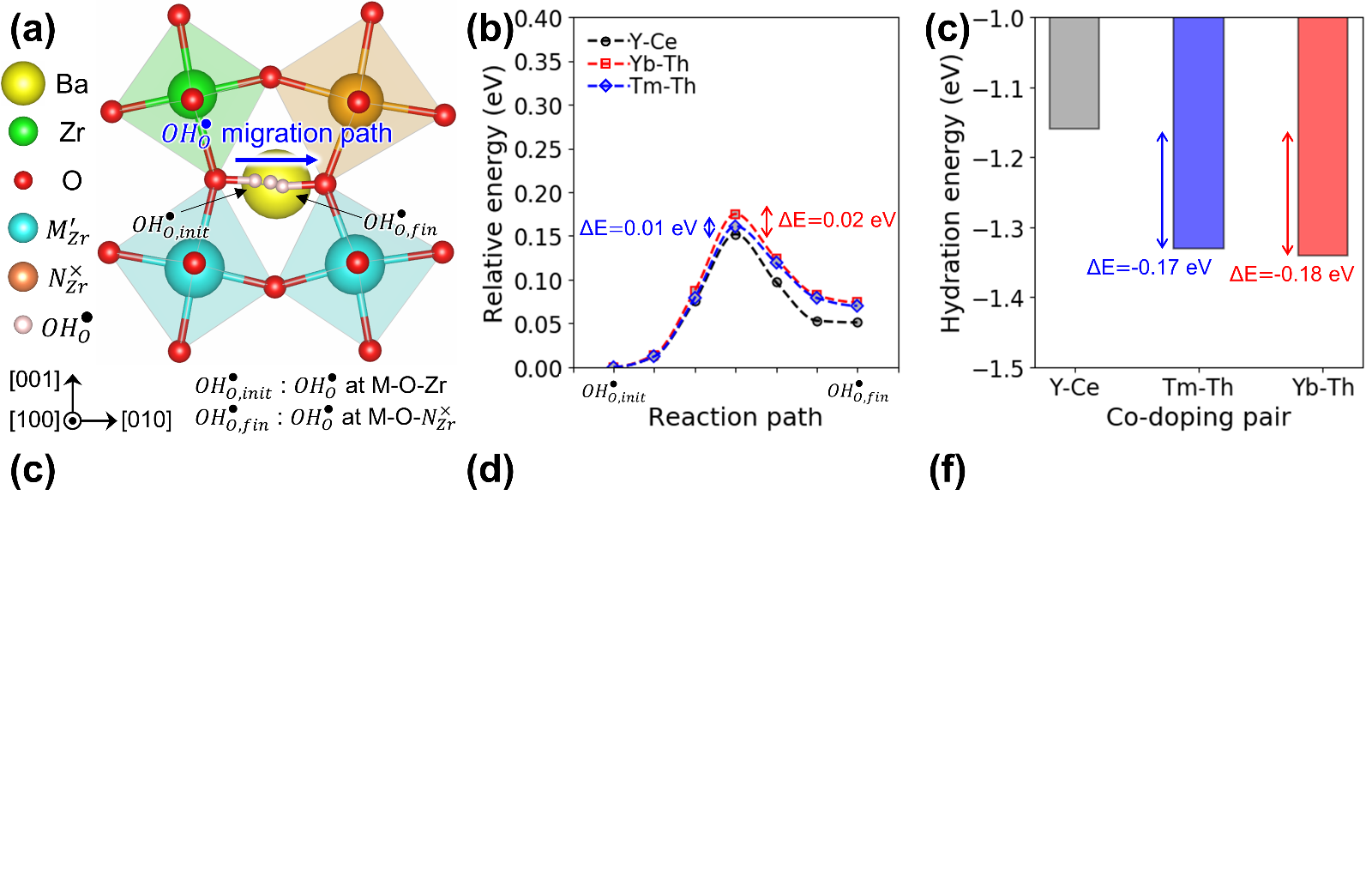


Figure S20. (a) Inter-octahedral migration path of proton (${OH}_{O}$) for the B-site isovalent ($N_{Zr}^{X}$) co-doping (Y-Ce, Yb-Th, and Tm-Th). (b) Migration barrier comparison between Y-Ce, Yb-Th, and Tm-Th. (c) Hydration energy comparison between Y-Ce, Yb-Th, and Tm-Th.


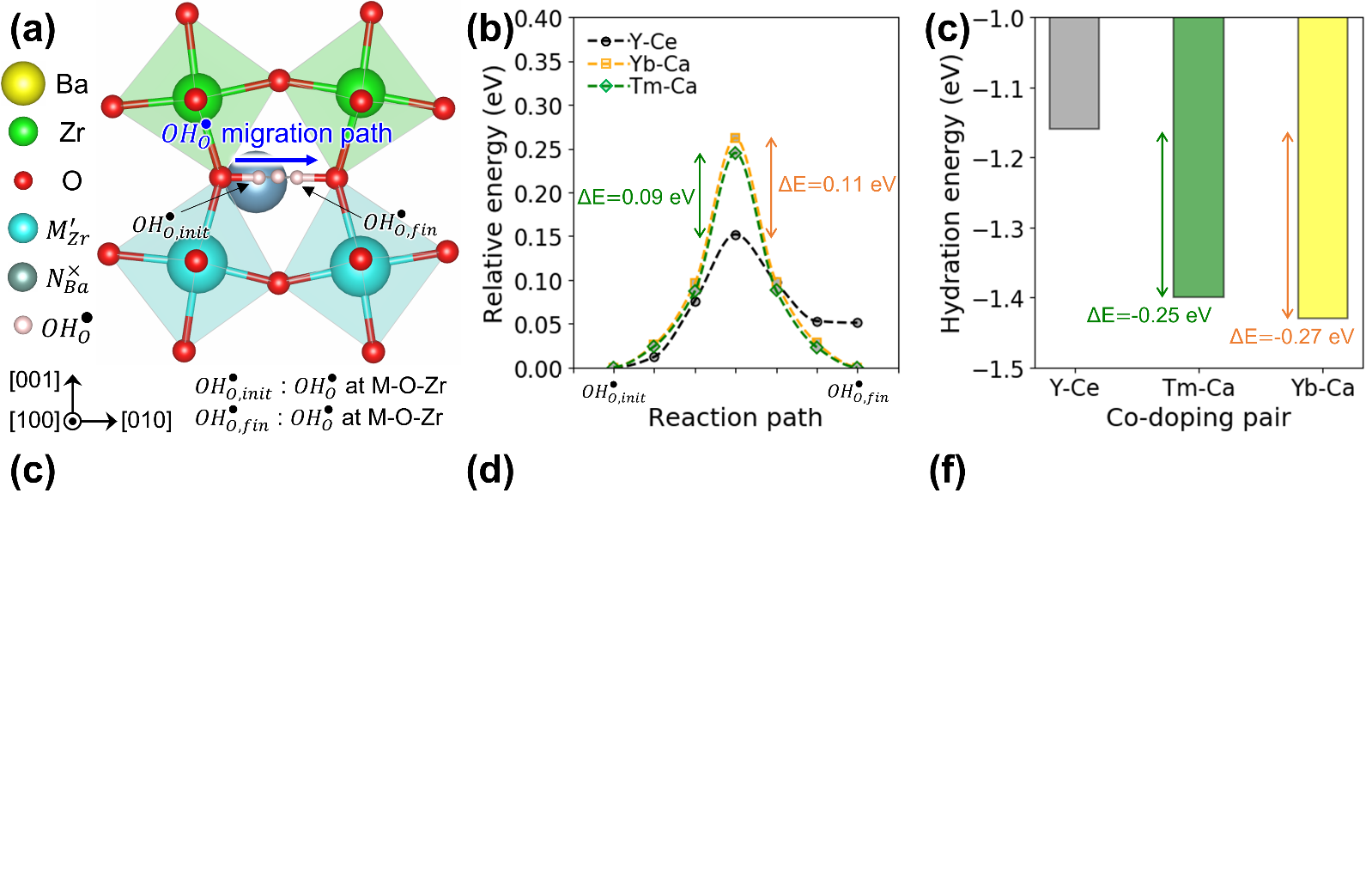


Figure S21. (a) Inter-octahedral migration path of proton (${OH}_{O}$) for the A-site isovalent ($N_{Ba}^{X}$) co-doping pairs (Yb-Ca and Tm-Ca). (b) Migration barrier comparison between Y-Ce, Yb-Ca and Tm-Ca. (c) Hydration energy comparison between Y-Ce, Yb-Ca and Tm-Ca.


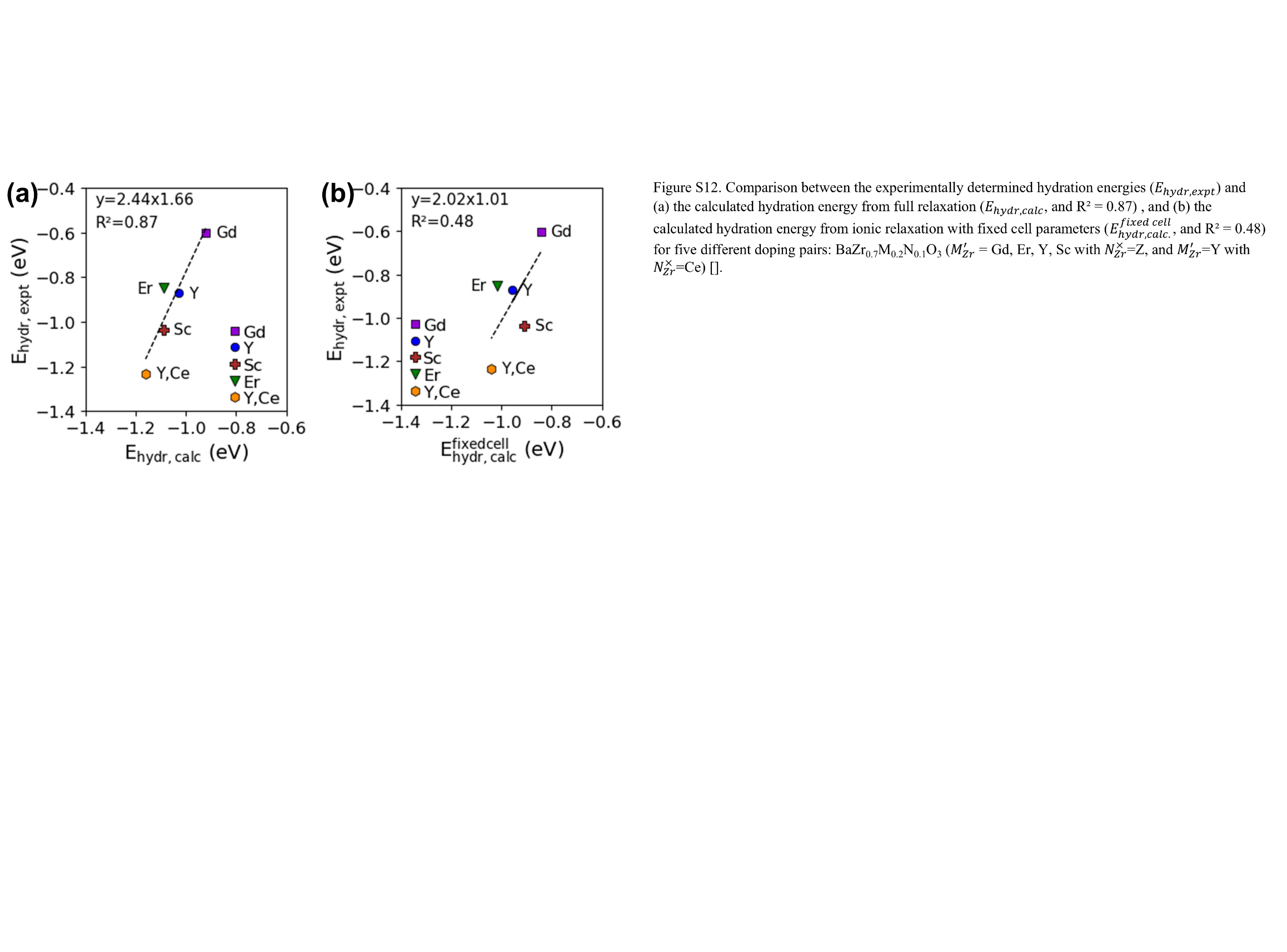


Figure S22. Comparison between the experimentally determined hydration energies ($E_{hydr, expt}$) and (a) the calculated hydration energy from full relaxation ($E_{hydr, calc}$, and R² = 0.87) , and (b) the calculated hydration energy from ionic relaxation with fixed cell parameters ($E_{hydr, calc.}^{fixed cell}$, and R² = 0.48) for five different doping pairs: BaZr0.7M0.2N0.1O3 ($M_{Zr}^{'}$ = Gd, Er, Y, Sc with $N_{Zr}^{\times}$=Z, and $M_{Zr}^{'}$=Y with $N_{Zr}^{\times}$=Ce).[11, 12]


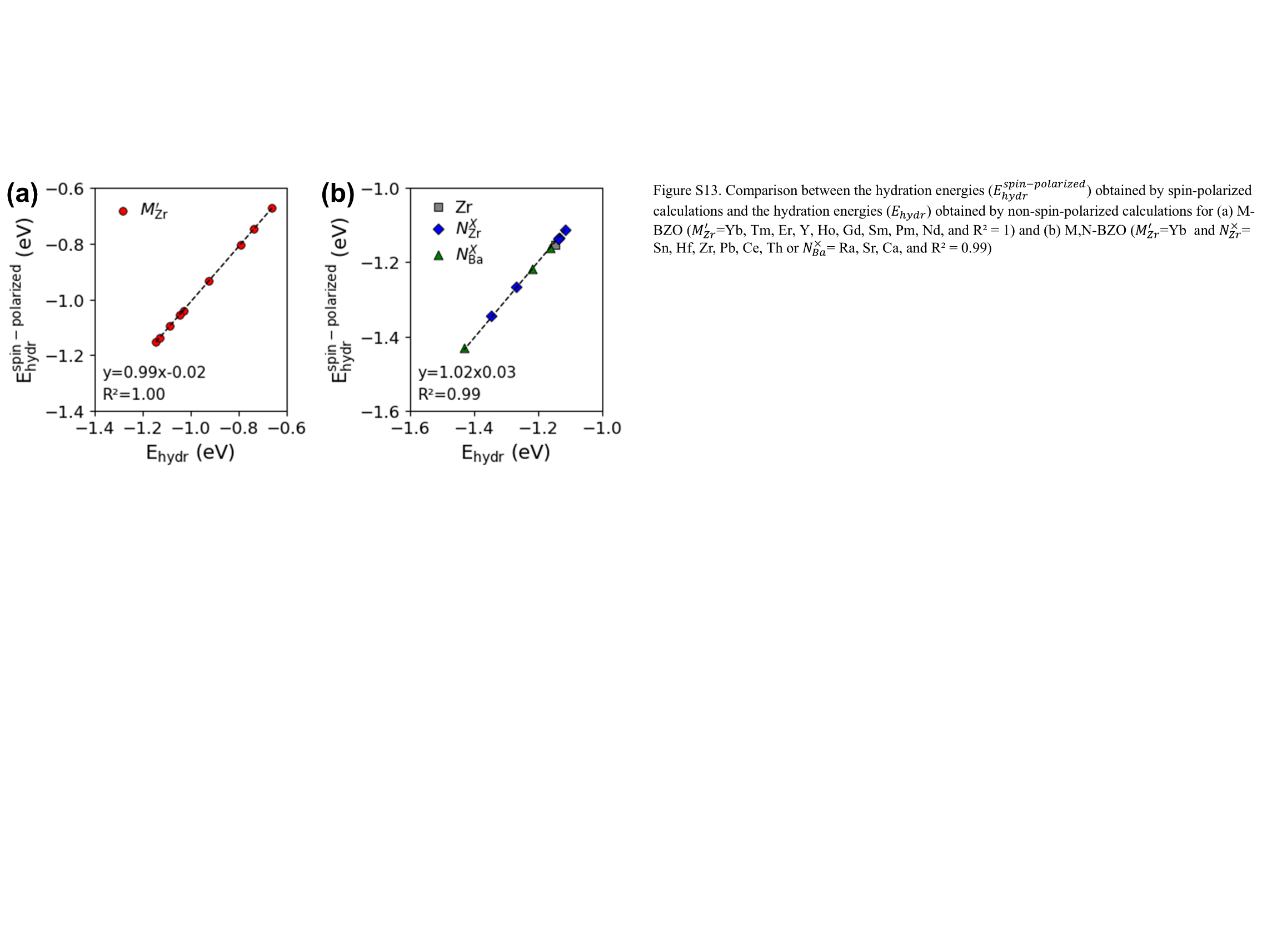


Figure S23. Comparison between the hydration energies ($E_{hydr}^{spin-polarized}$) obtained by spin-polarized calculations and the hydration energies ($E_{hydr}$) obtained by non-spin-polarized calculations for (a) M-BZO ($M_{Zr}^{'}$=Yb, Tm, Er, Y, Ho, Gd, Sm, Pm, Nd, and R² = 1) and (b) M,N-BZO ($M_{Zr}^{'}$=Yb and $N_{Zr}^{\times}$= Sn, Hf, Zr, Pb, Ce, Th or $N_{Ba}^{\times}$= Ra, Sr, Ca, and R² = 0.99)

| Composition | Atmosphere | Temperature(℃) | Phase | Ref. |
| --- | --- | --- | --- | --- |
| BaZr_1-x_M_x_O_3-δ_ (M=Yb, Tm, Er, Ho, and x=0.02, 0.05, 0.1, 0.15, 0.2, 0.25, 0.3) | Dry O_2_, wet O_2_ ($P_{H_{2}O}$ = 0.031atm) | 30 ~1000 | Cubic (Pm$\bar{3}$m) | 4 |
| BaZr_1-x_M_x_O_3-δ_ (M=In, Pr, Gd, Tb, Ho, Er, Tm, Yb; x=0.2) | Dry O_2_, wet O_2_ ($P_{H_{2}O}$ = 0.031atm) | 30 ~1000 | Cubic (Pm$\bar{3}$m) | 5 |
| BaZr_1-x_M_x_O_3-δ_  (M=Sc, Eu, Sm, Dy; x= 0.2) | Dry O_2_, wet O_2_ ($P_{H_{2}O}$ = 0.031atm) | 30 ~1000 | Cubic (Pm$\bar{3}$m) | 6 |
| BaZr_1-x-y_N_y_M_x_O_3-δ_ (M=Y; N=Ce; x=0.2; y=0-0.4) | Wet O_2_  ($P_{H_{2}O}$ = 0.031atm) | 30 ~1000 | Cubic (Pm$\bar{3}$m) | 7 |
| BaZr_1-x-y_N_y_M_x_O_3-δ_ (M=Y; N=Sn; x=0.20; y=0.10) | Air | Room temperature | Cubic (Pm$\bar{3}$m) | 8 |
| BaZr_1-x-y_N_y_M_x_O_3-δ_ (M=Y; N=Hf; x=0.20; y=0.05-0.25) | Air | Room temperature | Cubic (Pm$\bar{3}$m) | 9 |
| Ba_1-y_N_y_Zr_1-x_M_x_O_3-δ_ (M=Y; N=Sr; x=0.2; y=0.05-0.40) | Air | Room temperature | Cubic (Pm$\bar{3}$m) | 10 |
| Ba_1-y_N_y_Zr_1-x_M_x_O_3-δ_ (M=Y; N=Ca; x=0.2; y=0.05) | Air | Room temperature | Cubic (Pm$\bar{3}$m) | 10 |

Table S1. Structural phase of perovskite BZO doped with trivalent acceptors (BaZr1-xMxO3-δ) or co-doped with acceptors and isovalent elements (BaZr0.8-x-yMxNyO3-δ or Ba1-yNyZr0.8-xMxO3-δ) under various temperature and atmosphere.[4-10]

| Rare-earth dopant (M) | $E_{form}^{B-site}$ (eV/f.u.) | $E_{form}^{A-site}$ (eV/f.u.) | $\Delta E_{form} \left( E_{form}^{\boldsymbol{B}-site}-E_{form}^{A-site} \right)$ (eV/f.u.) |
| --- | --- | --- | --- |
| Yb | -1.080 | -0.942 | -0.138 |
| Tm | -1.078 | -0.947 | -0.131 |
| Y | -1.072 | -0.955 | -0.117 |
| Ho | -1.074 | -0.953 | -0.121 |
| Gd | -1.065 | -0.962 | -0.103 |
| Sm | -1.056 | -0.970 | -0.086 |
| Pm | -1.052 | -0.972 | -0.080 |
| Nd | -1.046 | -0.978 | -0.068 |

Table S2. DFT-calculated formation energies (eV/f.u.) of A-site doped Ba1-xMxZrO3 ($E_{form}^{A-site}$) and B-site doped BaZr1-xMxO3 ($E_{form}^{B-site}$), and their energy difference defined as $\Delta E_{form}$ = $E_{form}^{B-site}$ - $E_{form}^{B-site}$ for rare-earth dopants (M=Yb, Tm, Y, Ho, Gd, Sm, Pm, Nd; x=0.03125).

| $M_{Zr}^{'}$ | $N_{Zr}^{\times}$ | $N_{Ba}^{\times}$ | DFE[$V_{O}$] (eV) | | | DFE[$2{OH}_{O}$] (eV) | | |
| --- | --- | --- | --- | --- | --- | --- | --- | --- |
|  |  |  | PBE | HSE06 | \|ΔE\| | PBE | HSE06 | \|ΔE\| |
| Yb | Zr | Ba | 0.70 | 0.47 | 0.22 | -0.45 | -0.74 | 0.29 |
| Er | Zr | Ba | 0.60 | 0.20 | 0.40 | -0.48 | -0.97 | 0.48 |
| Y | Zr | Ba | 0.53 | 0.31 | 0.22 | -0.50 | -0.85 | 0.35 |
| Gd | Zr | Ba | 0.39 | 0.10 | 0.29 | -0.54 | -0.87 | 0.33 |
| Nd | Zr | Ba | 0.11 | -0.21 | 0.32 | -0.55 | -0.89 | 0.34 |
| Yb | Ce | Ba | 0.64 | 0.41 | 0.23 | -0.62 | -0.96 | 0.34 |
| Yb | Th | Ba | 0.57 | 0.34 | 0.23 | -0.77 | -1.14 | 0.37 |
| Yb | Zr | Ca | 0.68 | 0.46 | 0.26 | -0.75 | -1.08 | 0.33 |
| MAD | | | - | - | 0.28 | - | - | 0.36 |

Table S3. For BaZr_1-x_M_x_O_3_ ($M_{Zr}^{'}$=Yb, Er, Y, Gd, Nd), BaZr_1-x-y_M_x_N_y_O_3_ ($M_{Zr}^{'}$=Yb, $N_{Zr}^{\times}$= Ce, Th), and BaZr_1-x-y_M_x_N_y_O_3_ ($M_{Zr}^{'}$=Yb, $N_{Ba}^{\times}$=Ca), defect formation energy (DFE) of an oxygen vacancy ($V_{O}$) (DFE[$V_{O}$]) and DFE of two protons (2${OH}_{O}$) (DFE[$2{OH}_{O}$]) calculated using PBE and HSE06 functionals. |ΔE|: the absolute difference between PBE and HSE06 values; MAD mean absolute deviation of |ΔE|.

| Dopant | $E_{hydr}$ (eV) | Acceptor contribution (eV) | Isovalent contribution (eV) | Total improvement (eV) |
| --- | --- | --- | --- | --- |
| Y (ref.) | -1.03 | - | - | - |
| Yb | -1.14 | -0.11 | - | -0.11 |
| Tm | -1.13 | -0.10 | - | -0.10 |
| Yb-Th | -1.34 | -0.11 (35%) | -0.20 (65%) | -0.31 |
| Tm-Th | -1.33 | -0.10 (33%) | -0.20 (67%) | -0.30 |
| Yb-Ca | -1.43 | -0.11 (28%) | -0.29 (72%) | -0.40 |
| Tm-Ca | -1.40 | -0.10 (27%) | -0.27 (73%) | -0.37 |

Table S4. Quantitative decomposition of hydration energy ($E_{hydr}$) contributions for the proposed co-doping pairs (Yb-Th, Tm-Th, Yb-Ca, Tm-Ca)

| Composition | $\Delta E_{carbonation}$(eV) | $\Delta E_{carbonation}^{relative}$(eV) | $\Delta E_{hydroxylation}$(eV) | $\Delta E_{hydroxylation}^{relative}$(eV) |
| --- | --- | --- | --- | --- |
| BaCeO_3_ | -2.24 | 0 | -1.21 | 0 |
| BaZrO_3_ | -1.57 | 0.67 | -0.55 | 0.66 |
| BaZr_1-x-y_N_y_MxO_3_ (M=Yb; N=Th; x=0.0625; y=0.03125) | -1.70 | 0.54 | -0.68 | 0.53 |
| Ba_1-y_N_y_Zr_1-x_MxO_3_ (M=Yb; N=Ca; x=0.0625; y=0.03125) | -1.66 | 0.58 | -0.65 | 0.56 |
| BaZr_1-x-y_N_y_MxO_3_ (M=Tm; N=Th; x=0.0625; y=0.03125) | -1.71 | 0.53 | -0.68 | 0.53 |
| Ba_1-y_N_y_Zr_1-x_MxO_3_ (M=Tm; N=Ca; x=0.0625; y=0.03125) | -1.67 | 0.57 | -0.66 | 0.55 |

Table S5. DFT-calculated reaction energies for carbonation ($\Delta E_{carbonation}$) and hydroxylation ($\Delta E_{hydroxylation}$) of BaCeO_3_, BaZrO_3_, BaZr_1-x-y_N_y_MxO_3_ (M=Yb, Tm; N=Th; x=0.0625; y=0.03125), and Ba_1-y_N_y_Zr_1-x_MxO_3_ (M=Yb, Tm; N=Ca; x=0.0625; y=0.03125). $\Delta E_{carbonation}^{relative}$ and $\Delta E_{hydroxylation}^{relative}$ denote the differences in $\Delta E_{carbonation}$ and $\Delta E_{hydroxylation}$ relative to BaCeO_3_ with more positive values indicating greater stability.

| $M_{Zr}^{'}$ | $N_{Zr}^{\times}$ | $N_{Ba}^{\times}$ | DFE[$V_{O}$] (eV) | DFE  [$2{OH}_{O}$] for config.1 (eV) | DFE  [$2{OH}_{O}$] for config.2 (eV) | DFE  [$2{OH}_{O}$] for config.3 (eV) | $E_{hydr}$  (eV) |
| --- | --- | --- | --- | --- | --- | --- | --- |
| Yb | Zr | Ba | 0.70 | -0.32 | -0.45 | NaN | -1.14 |
| Tm | Zr | Ba | 0.65 | -0.36 | -0.48 | NaN | -1.13 |
| Er | Zr | Ba | 0.60 | -0.36 | -0.49 | NaN | -1.09 |
| Y | Zr | Ba | 0.53 | -0.36 | -0.50 | NaN | -1.03 |
| Ho | Zr | Ba | 0.55 | -0.36 | -0.49 | NaN | -1.04 |
| Gd | Zr | Ba | 0.39 | -0.40 | -0.54 | NaN | -0.92 |
| Sm | Zr | Ba | 0.27 | -0.38 | -0.52 | NaN | -0.79 |
| Pm | Zr | Ba | 0.20 | -0.40 | -0.53 | NaN | -0.74 |
| Nd | Zr | Ba | 0.11 | -0.42 | -0.55 | NaN | -0.66 |

Table S6. For M-BZO ($M_{Zr}^{'}$=Yb, Tm, Er, Y, Ho, Gd, Sm, Pm, Nd), Defect formation energy of $V_{O}$ (DFE[$V_{O}$]), DFE[2${OH}_{O}$ ] for config. 1-3, and $E_{hydr}$.

| $M_{Zr}^{'}$ | $N_{Zr}^{\times}$ | $N_{Ba}^{\times}$ | DFE[$V_{O}$] (eV) | DFE  [$2{OH}_{O}$] for config.1 (eV) | DFE  [$2{OH}_{O}$] for config.2 (eV) | DFE  [$2{OH}_{O}$] for config.3 (eV) | $E_{hydr}$  (eV) |
| --- | --- | --- | --- | --- | --- | --- | --- |
| Yb | Zr | Ba | 0.70 | -0.32 | -0.45 | NaN | -1.14 |
| Yb | Sn | Ba | 0.81 | -0.16 | -0.31 | -0.18 | -1.11 |
| Yb | Hf | Ba | 0.80 | -0.20 | -0.33 | -0.22 | -1.14 |
| Yb | Pb | Ba | 0.81 | -0.32 | -0.30 | -0.20 | -1.14 |
| Yb | Ce | Ba | 0.64 | -0.62 | -0.44 | -0.44 | -1.26 |
| Yb | Th | Ba | 0.57 | -0.77 | -0.40 | -0.54 | -1.34 |
| Yb | Zr | Ca | 0.68 | -0.75 | -0.66 | NaN | -1.43 |
| Yb | Zr | Sr | 0.64 | -0.55 | -0.58 | NaN | -1.22 |
| Yb | Zr | Ra | 0.77 | -0.24 | -0.39 | NaN | -1.16 |

Table S7. For M,N-BZO ($M_{Zr}^{'}$=Yb and $N_{Zr}^{\times}$= Sn, Hf, Zr, Pb, Ce, Th or $N_{Ba}^{\times}$= Ra, Sr, Ca), DFE[$V_{O}$], DFE[2${OH}_{O}$ ] for config. 1-3, and $E_{hydr}$.

| $M_{Zr}^{'}$ | $N_{Zr}^{\times}$ | $N_{Ba}^{\times}$ | DFE[$V_{O}$] (eV) | DFE  [$2{OH}_{O}$] for config.1 (eV) | DFE  [$2{OH}_{O}$] for config.2 (eV) | DFE  [$2{OH}_{O}$] for config.3 (eV) | $E_{hydr}$  (eV) |
| --- | --- | --- | --- | --- | --- | --- | --- |
| Yb | Ce | Ba | 0.64 | -0.62 | -0.44 | -0.44 | -1.26 |
| Tm | Ce | Ba | 0.60 | -0.64 | -0.48 | -0.48 | -1.25 |
| Er | Ce | Ba | 0.55 | -0.66 | -0.48 | -0.50 | -1.21 |
| Y | Ce | Ba | 0.48 | -0.68 | -0.50 | -0.56 | -1.16 |
| Ho | Ce | Ba | 0.50 | -0.66 | -0.48 | -0.56 | -1.17 |
| Yb | Th | Ba | 0.57 | -0.76 | -0.40 | -0.54 | -1.34 |
| Tm | Th | Ba | 0.54 | -0.80 | -0.44 | -0.57 | -1.33 |
| Er | Th | Ba | 0.48 | -0.80 | -0.44 | -0.60 | -1.29 |
| Y | Th | Ba | 0.42 | -0.82 | -0.45 | -0.64 | -1.24 |
| Ho | Th | Ba | 0.45 | -0.82 | -0.44 | -0.62 | -1.26 |

Table S8. For M,N-BZO ($M_{Zr}^{'}$=Yb, Tm, Er, Y, Ho and $N_{Zr}^{\times}$= Ce, Th), DFE[$V_{O}$], DFE[2${OH}_{O}$ ] for config. 1-3, and $E_{hydr}$.

| $M_{Zr}^{'}$ | $N_{Zr}^{\times}$ | $N_{Ba}^{\times}$ | DFE[$V_{O}$] (eV) | DFE  [$2{OH}_{O}$] for config.1 (eV) | DFE  [$2{OH}_{O}$] for config.2 (eV) | DFE  [$2{OH}_{O}$] for config.3 (eV) | $E_{hydr}$  (eV) |
| --- | --- | --- | --- | --- | --- | --- | --- |
| Yb | Zr | Sr | 0.64 | -0.55 | -0.58 | NaN | -1.22 |
| Tm | Zr | Sr | 0.60 | -0.56 | -0.60 | NaN | -1.20 |
| Er | Zr | Sr | 0.55 | -0.56 | -0.62 | NaN | -1.16 |
| Y | Zr | Sr | 0.45 | -0.60 | -0.64 | NaN | -1.10 |
| Ho | Zr | Sr | 0.47 | -0.60 | -0.66 | NaN | -1.12 |
| Yb | Zr | Ca | 0.68 | -0.75 | -0.66 | NaN | -1.43 |
| Tm | Zr | Ca | 0.66 | -0.74 | -0.66 | NaN | -1.40 |
| Er | Zr | Ca | 0.60 | -0.74 | -0.68 | NaN | -1.35 |
| Y | Zr | Ca | 0.45 | -0.76 | -0.78 | NaN | -1.22 |
| Ho | Zr | Ca | 0.56 | -0.76 | -0.70 | NaN | -1.30 |

Table S9. For M,N-BZO ($M_{Zr}^{'}$=Yb, Tm, Er, Y, Ho and $N_{Ba}^{\times}$= Sr, Ca), DFE[$V_{O}$], DFE[2${OH}_{O}$ ] for config. 1-3, and $E_{hydr}$.

Reference

| 1 | G. Kresse and J. Furthmüller, *Phys. Rev. B*, **1996**, 54, 11169. |
| --- | --- |
| 2 | P. E. Blöchl, *Phys. Rev. B*, **1994**, 50, 17953. |
| 3 | S. Grimme, J. Antony, S. Ehrlich and H. Krieg, *J. Chem. Phys.*, **2010**, 132, 154104 |
| 4 | D. Han, N. Hatada and T. Uda, *J. Electrochem. Soc.*, **2016**, 163, F470. |
| 5 | D. Han, K. Shinoda, S. Sato, M. Majima and T. Uda, *J. Mater. Chem. A*, **2015**, 3, 1243. |
| 6 | D. Han, K. Shinoda and T. Uda, *J. Am. Ceram. Soc.*, **2014**, 97, 643. |
| 7 | D. Han, X. Liu, T. S. Bjørheim and T. Uda, *Adv. Energy Mater.*, **2021**, 11, 2003149 |
| 8 | W. Sun, M. Liu and W. Liu, *Adv. Energy Mater.*, **2013**, 3, 1041. |
| 9 | W. Yang, L. Wang, Y. Li, H. Zhou, Z. He, C. Han and L. Dai, *Ceram. Int.*, **2021**, 47, 9273. |
| 10 | K. Kato, D. Han and T. Uda, *J. Am. Ceram. Soc.*, **2019**, 102, 1201. |
| 11 | J. Hyodo, K. Tsujikawa, M. Shiga, Y. Okuyama and Y. Yamazaki, *ACS Energy Lett.*, **2021**, 6, 2985. |
| 12 | K. Hoshino, S. Kasamatsu, J. Hyodo, K. Yamamoto, H. Setoyama, T. Okajima and Y. Yamazaki, *Chem. Mater.*, **2023**, 35, 2289. |
| 13 | J. H. Al Shuhaib, I. J. Ferrer, J. R. Ares, S. Cianci, F. Tuzi, E. Blundo, A. Polimeni, A. Benayas, R. Marin, F. Leardini, *J. Mater. Chem. C* **2025**, *13*, 2238. |
| 14 | P. G. Sundell, M. E. Björketun, G. Wahnström, *Phys. Rev. B* **2006**, *73*. |
| 15 | J. Ding, J. Balachandran, X. Sang, W. Guo, J. S. Anchell, G. M. Veith, C. A. Bridges, Y. Cheng, C. M. Rouleau, J. D. Poplawsky, N. Bassiri-Gharb, R. R. Unocic, P. Ganesh, *Chem. Mater.* **2018**, *30*, 4919. |
| 16 | J. Nisar, C. Århammar, E. Jämstorp, R. Ahuja, *Phys. Rev. B* **2011**, *84*. |
| 17 | J. L. Lyons, C. G. Van de Walle, *Npj Comput. Mater.* **2017**, *3*, 12. |
| 18 | A. Fluri, A. Marcolongo, V. Roddatis, A. Wokaun, D. Pergolesi, N. Marzari, T. Lippert, *Adv. Sci.* **2017**, *4*, 1700467. |
| 19 | Y.-C. Jeong, B.-K. Kim, Y.-C. Kim, *Solid State Ion.* **2014**, *259*, 1. |
| 20 | A. K. Azad, J. T. S. Irvine, *Chem. Mater.* **2009**, *21*, 215. |
| 21 | M. Saiful Islam, *J. Mater. Chem.* **2000**, *10*, 1027. |
| 22 | D. Han, X. Liu, T. S. Bjørheim, T. Uda, *Adv. Energy Mater.* **2021**, *11*, 2003149. |
| 23 | D. Han, K. Shinoda, S. Sato, M. Majima, T. Uda, *J. Mater. Chem. A* **2015**, *3*, 1243. |
| 24 | F. M. Draber, C. Ader, J. P. Arnold, S. Eisele, S. Grieshammer, S. Yamaguchi, M. Martin, *Nat. Mater.* **2020**, *19*, 338. |
| 25 | C. Y. Regalado Vera, H. Ding, J. Urban-Klaehn, M. Li, Z. Zhao, F. Stewart, H. Tian, X. Liu, Y. Dong, J. Li, M. Zhou, H. Luo, D. Ding, *Chem. Mater.* **2023**, *35*, 5341. |
